# Studying the dynamics of mandibular growth spurts in individuals with Class I and Class II skeletal growth patterns using the Bayesian superimposition by translation and rotation (SITAR) model

**DOI:** 10.1093/ejo/cjaf088

**Published:** 2025-11-11

**Authors:** Satpal S Sandhu, George Leckie, Kate Tilling, Rachael A Hughes

**Affiliations:** Centre for Multilevel Modelling, University of Bristol, 35 Berkeley Square, Clifton, Bristol BS8 1JA, United Kingdom; MRC Integrative Epidemiology Unit, University of Bristol, Oakfield House, Oakfield Grove, Clifton, Bristol BS8 2BN, United Kingdom; Centre for Multilevel Modelling, University of Bristol, 35 Berkeley Square, Clifton, Bristol BS8 1JA, United Kingdom; MRC Integrative Epidemiology Unit, University of Bristol, Oakfield House, Oakfield Grove, Clifton, Bristol BS8 2BN, United Kingdom; MRC Integrative Epidemiology Unit, University of Bristol, Oakfield House, Oakfield Grove, Clifton, Bristol BS8 2BN, United Kingdom

**Keywords:** mandibular growth spurt, Bayesian growth modeling, Superimposition by Translation and Rotation (SITAR) model

## Abstract

**Background:**

Comparing the mandibular growth patterns of Class I and Class II children offer valuable insights into adolescent growth dynamics that are crucial for planning growth modification procedures.

**Aim:**

To test the null hypothesis of no class differences (Class I vs. Class II) in mandibular growth spurt parameters (size, timing, intensity) versus clinically relevant differences defined as ≥ 5 mm for size, ≥ 1 year for timing, and ≥ 1 mm/year for intensity

**Subjects and methods:**

Data (Condyle–Pogonion distance) available from the American Association of Orthodontists Foundation Craniofacial Growth Legacy Collection is analyzed. The dataset includes 160 children, 40 each in Class I and Class II groups for both sexes. A three-level Bayesian SITAR model is fitted, incorporating individual- and study-level random effects for all three growth spurt parameters.

**Results:**

The average mandibular size is greater in Class I than in Class II for both males [5.0 mm, 95% CI (3.0, 7.0)] and females [4.4 mm, 95% CI (3.2, 5.7)]. The growth spurt occurs later for Class I than Class II across both sexes [males: 0.6 years, 95% CI (0.3, 0.9); females: 0.6 years, 95% CI (0.2, 0.9)], and the intensity of the growth spurt is slightly greater in Class I than in Class II [males: 0.2 mm/year, 95% CI (0.0, 0.4); females: 0.2 mm/year, 95% CI (0.0, 0.4)]. The results show individual-level variation in size, timing, and intensity parameters across both Class II and Class I.

**Conclusions:**

No evidence found for clinically meaningful class differences in the timing or intensity of the adolescent growth spurt dynamics for either sex. The mandibular deficiency observed in Class II children is established early, during the pre-adolescent growth period. The cumulative deficiency in mandibular size for Class II children worsens with age, which leads to a more pronounced skeletal discrepancy in adulthood. The clinical implications for treatment planning are discussed.

**Limitations:**

Considering the retrospective nature of the historical data analyzed, caution is warranted when generalizing our findings to contemporary populations.

## Introduction

Class II skeletal malocclusion is a commonly observed clinical issue in orthodontic practice [1]. Mandibular deficiency is the most prevalent feature of Class II skeletal malocclusion [2], and growth modification is the treatment of choice for correcting it during periods of active growth in children [2–5]. Growth modification procedures are most effective when treatment timing is carefully aligned with the timing of the adolescent growth spurt [2–5].

While researchers have used different approached to determine the timing of the adolescent growth spurt (such as sexual maturation assessment, chronological and dental age, and radiograph-based methods) [2, 6, 7], methods based on longitudinal growth data are the most accurate in determining the skeletal maturation [8, 9]. The peak growth velocity is a biological maturity indicator that reflects the maximum skeletal growth during adolescence [9]. The age at peak growth velocity is considered the gold standard for objectively assessing the timing of the growth spurt during adolescence [9]. Estimation of the peak growth velocity and the age at peak growth velocity involves fitting an appropriate statistical model to longitudinal growth data [9]. Mixed-effects models (also known as multilevel models) are popular as they can easily handle many complexities of longitudinal data such as variation in the number of growth measurements per child and in the timing of those measurements [9, 10]. Importantly, mixed-effects models allow the estimation of individual-level variability in the growth parameters [9, 10].

The Superimposition by Translation and Rotation (SITAR) model is a nonlinear mixed-effects model and has been used extensively to analyze human growth data such as height [11]. The SITAR model has been successfully applied to analyze height data [12, 13]. The SITAR model fits a natural cubic spline-based mean curve to the data and aligns individual-specific growth trajectories to this mean curve via a set of three random effects [11]: (i) size (vertical shift of the mean curve, up or down), (ii) timing of the adolescent growth spurt (horizontal shift of the mean curve, left or right), and (iii) the intensity of the adolescent growth spurt (horizontal stretching or shrinking of the mean curve). Thus, whereas the mean curve estimates the average (i.e. mean across children) adolescent growth spurt parameters (size, timing, and intensity), the random effects capture each child’s deviation from these average values. A recent study [17] applied the SITAR model to mandibular growth data from children with normal Class I growth patterns and demonstrated its suitability for analyzing radiographically derived mandibular size measurements. However, the full model with all random effects failed to converge [14]. This is perhaps due to the small sample size (18 males, 21 females) analyzed in that study [14].

While mixed-effects models can be estimated using Bayesian and frequentist (e.g. maximum likelihood) approaches [15], the Bayesian approach offers distinct advantages. It allows for the integration of prior information with data and provides inferences that are conditional on the data without relying on asymptotic approximations. This capability enables the fitting of complex models even with relatively small sample sizes [16]. From a decision-making perspective, Bayesian analysis provides clinically meaningful interpretations, such as the statement that ‘the true parameter has a 0.95 probability of falling within a 95% credible interval’.

In this article, a novel Bayesian SITAR model is applied to study and compare mandibular growth between children having Class I and Class II skeletal growth patterns. The aim is to describe mandibular growth from early childhood through adulthood for Class I and Class II growth patterns with an emphasis on understanding class differences in the timing and intensity of the adolescent growth spurt.

The primary objective is to compare skeletal growth between Class I and Class II children at the population level and test null hypotheses of no class differences (Class I vs. Class II) in mandibular growth spurt parameters (size, timing, intensity) versus clinically relevant differences (size ≥ 5 mm, timing ≥ 1 year, intensity ≥ 1 mm/year). Evidence suggests that 5 mm of growth modification to correct Class II malocclusion is the maximum that should be anticipated [17]. A difference of 1.0 mm/year in average growth velocity between Class I and Class II implies a cumulative size difference of 5.0 mm over a period of 5 years.

The secondary objective addresses the between-individual variability in these parameters, with a corresponding hypothesis that variability differences in size, timing, and intensity between Class I and Class II are clinically non relevant. For size and timing, we test class differences in the SD parameters that summarize the variability in the size and timing random effects. For intensity, we test class differences in terms of percentage (10%). Since the intensity parameter is modeled as an exponential, the SD of intensity is defined as a fractional multiplier (or as percentage, SD × 100).

## Materials and methods

The mandibular growth data [Condyle to Pogonion distance (COPOD)] available from the American Association of Orthodontists Foundation (AAOF) Craniofacial Growth Legacy Collection database [18] is analyzed in this study. The AAOF Craniofacial Growth Legacy Collection comprises data pooled from nine historical growth studies (Bolton, Burlington, Denver, Fels, Forsyth, Iowa, Mathews, Michigan, and Oregon) conducted between 1930 and 1985 [18]. Children included in these studies were predominantly Anglo-Saxon or Caucasian [18].

The data included in the AAOF Craniofacial Growth Legacy Collection were obtained from children with no known history of orthodontic treatment, serving as a valuable resource of longitudinal craniofacial growth records in untreated children [18]. The AAOF Craniofacial Growth Legacy Collection provides the methodological consistency essential for mitigating systematic variability when pooling data across multiple growth studies. This standard is ensured by rigorous imaging protocols, including standardized digitization and magnification error correction for all lateral cephalograms and their derived measurements [19].

A subset of data originally used in a previous study [19] was analyzed in this article. The previous study [19] provides a thorough account of the methods for measuring mandibular length on lateral cephalograms, including validation of measurement reliability through assessments of both systematic and random errors. The data that met the following inclusion criteria were incorporated into this analysis: (i) age range between 6 and 20 years, (ii) data available for both Class I and Class II skeletal malocclusions, (iii) data available for at least two children in each growth study, and (iv) a minimum of four repeated measurements per child and at least one measurement in each of the following growth periods: from 6 years to 10 years, from 10 years up to 15 years, and between 15 and 20 years. The Fels growth study provides data for only one child who met the inclusion criteria and, therefore, is excluded from the analysis. We also excluded the Forsyth and Iowa studies because data were available only for Class I children. Therefore, the data included in this study is drawn from the six growth studies (Bolton, Burlington, Denver, Mathews, Michigan, and Oregon). The specific data utilized for this analysis were collected from 1930 to 1967.

Data are analyzed separately for males and females using the Bayesian SITAR model. Supplementary file (Section S1) provides a detailed description of the SITAR model, accompanied by a schematic diagram (Supplementary Figure S1) that illustrates how the growth parameters (size, timing, and intensity) collectively shape the growth trajectory. Section S2 of the Supplementary file describes the data used in the analysis. A three-level SITAR model is fit to fully account for the hierarchical dependency in the three-level data structure in which repeated jaw measurements on children are further nested within growth studies (see Supplementary file, Section S3). To estimate class-specific population average growth trajectories, we include Class as a covariate in the fixed effect structure of the model. The model is fit with unconstrained variance–covariance structures at both individual- and study levels thereby allowing growth curves to vary freely across children and studies. At the individual-level, we estimate variance–covariance parameters separately for each class thereby avoiding the unrealistic assumption that Class I and Class II children follow the same growth pattern. The residual variance is estimated separately for Class I and Class II. We use normal distribution-based priors for all parameters. For the standard deviation of group-level random effects (children and growth studies), we use half-normal priors. Details on priors used, and the plausible values covered by the prior distribution (such as the 95% probability mass) for each parameter, are provided in the supplementary file (Section S3).

As part of the model optimization [20], we fit Bayesian SITAR models to untransformed data (i.e. age in years) and log-transformed age measures and also vary the degrees of freedom for the natural cubic spline. The approximate leave-one-out cross-validation (LOO) and Watanabe information criterion (WAIC) are used to compare model fit [21]. We perform posterior predictive checks (PPC) to ascertain whether the model can explain the data-generative mechanism of the observed data [22]. We use quantile–quantile (QQ) plots to check the normality assumption of the residuals and fitted-versus-residual plots to examine any potential violations of the homoscedasticity assumption.

For hypothesis testing, we use the Bayesian null-hypothesis testing framework that includes the Probability of Direction (PD) and the Region of Practical Equivalence, ROPE [23]. The PD quantifies the probability (in percentage) of the existence of the effect (e.g. class difference in size is greater than zero). The ROPE, which defines the range of clinically meaningful differences in parameters, is typically used in decision-making processes [23]. If the high-density interval (HDI) of a parameter estimate is completely outside the ROPE limits (e.g. 95%), there is evidence of a clinically meaningful difference. If the ROPE entirely covers the HDI, the null hypothesis is accepted, and there is no clinically meaningful difference. In the case that the ROPE partially covers the HDI, it is ‘undecided’ whether to accept or reject the null hypothesis [23].

We use the recently developed ‘*bsitar*’ package, version 0.2.1 [24] within R, version 4.0.2 [25] to fit the Bayesian SITAR model. The ‘*bsitar*’ package uses the ‘*brms*’ package, version 2.22.0 [26] which calls Stan’s Hamiltonian Monte Carlo (HMC) algorithm, and its extension, the no-U-turn sampler (NUTS), for sampling [27]. We fit the Bayesian SITAR model using four Markov chains with 2000 iterations per chain, with 1000 iterations from each chain discarded as warm-up. Each chain is initiated using random initial values (default setting of Stan). The convergence of Markov Chain Monte Carlo (MCMC) chains is assessed using diagnostic plots (density and trace plots), as well as by evaluating the R-hat values and the bulk and tail effective sample sizes of the posterior draws [28]. The ‘*priorsense*’ package (version 1.0.4) is used to perform prior sensitivity analysis which implements power-scaling perturbations of the prior distribution to diagnose any ‘prior-data’ or ‘prior-likelihood’ conflicts [29].

## Results

Table 1 summarizes the data analyzed in this study. The data include 160 children, comprising 80 males and 80 females, with 40 participants in each of the Class I and Class II groups. The characteristics of the Class I and Class II groups are similar for both males and females. Supplementary file (Supplementary Table S1) provides a detailed description of class- and study-specific sample characteristics for both sexes and shows the observed growth trajectories (Supplementary Figure S2).

**Table 1.** Summary of the data analyzed in this study, including sample size and demographic characteristics for Class I and Class II groups, stratified by sex.

Sex	Class	*N* ^a^	Obs^b^	Age (years)			Measurements		
				Mean	SD^c^	Range^d^	Median	IQR^e^	Range^e^
Male	Class I	40	365	12.1	3.6	6 to 20	10	8 to 11	4 to 14
	Class II	40	352	12.0	3.3	6 to 20	9	8 to 10	5 to 14
Female	Class I	40	345	12.1	3.4	6 to 20	9	8 to 11	4 to 14
	Class II	40	345	12.1	3.4	6 to 20	9	8 to 10	5 to 13

^a^Number of individuals.

^b^Total number of observations.

^c^Standard deviation.

^d^Range: minimum to maximum.

^e^IQR, interquartile range: Q1 to Q3.

The results (LOO and WAIC) show that models with 5 and 4 degrees of freedom fit best to the male and female data, respectively. For both sexes, the model applied to the original data (i.e. age in years) provided a better fit than the log-transformed age data. The prior sensitivity analysis showed no ‘prior-data’ or ‘prior-likelihood’ conflicts (see Supplementary File, Section S4). For both sexes, the PPC plots show good model fit to data (see Supplementary File, Section S5), and the residual plots show no violation of the normality or homoscedasticity assumptions of the residuals (see Supplementary File, Section S6). The MCMC diagnostic plots (see Supplementary File, Section S7) show good convergence of chains for all model parameters. For each parameter, the R-hat is below 1.01, and the bulk- and tail-effective sample sizes are above 1000.

### Growth curves

**Figure 1.**
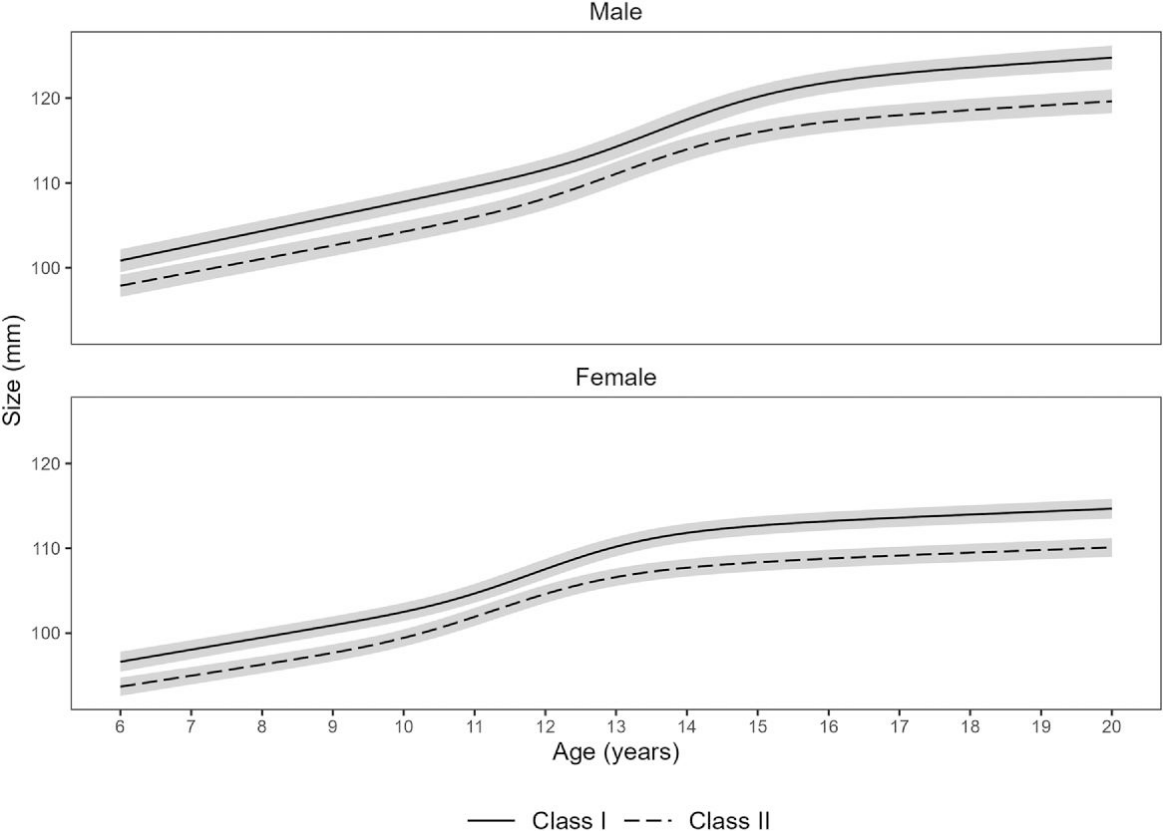
Class-specific mandibular growth trajectories for male and females. The mean cumulative curves illustrate that while both Class I and Class II groups follow a similar growth pattern, the gain in mandibular size is consistently greater for Class I than Class II. These findings suggest that the characteristic mandibular deficiency in Class II malocclusion is established early and persists throughout the growth period.

**Figure 2.**
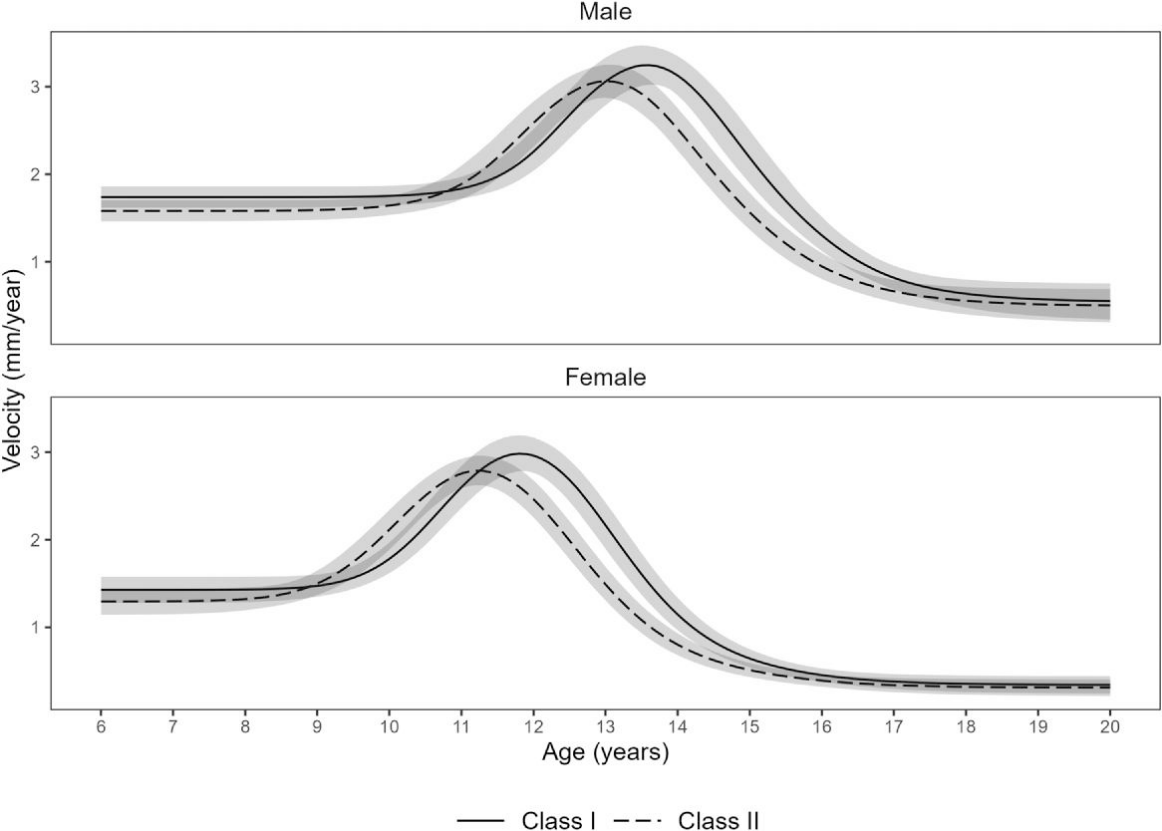
Class-specific mean growth velocity curves, stratified by sex. While both Class I and Class II groups exhibit a distinct adolescent growth spurt, the peak of the velocity curve for Class II is shifted to the left (indicating an earlier spurt) and is vertically lower (indicating a less intense spurt) compared to Class I.

Class-specific growth trajectories (marginalized over random effects) for males and females are shown in Fig. 1. For both sexes, mandibular size is smaller for Class II than for Class I throughout the growth period studied. Growth velocity curves (Fig. 2) show a distinct adolescent growth spurt for both Class I and Class II. For both sexes, the growth spurt is less intense and occurs earlier for Class II than for Class I.

**Figure 3.**
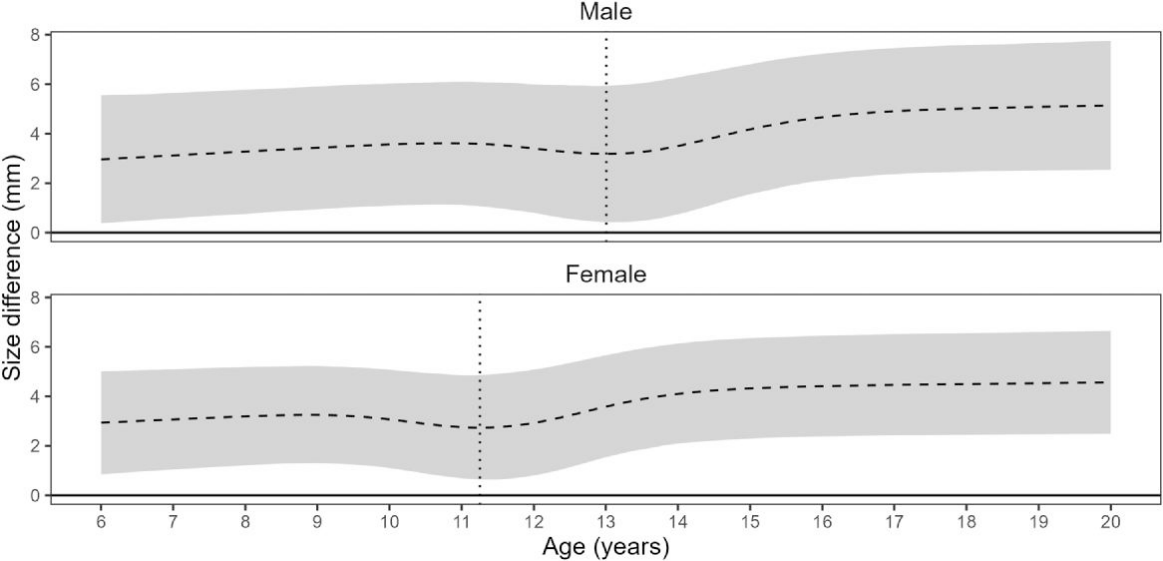
Mean difference in mandibular size (Class I − Class II) for male and females. The plot illustrates the dynamic nature of the class differences in the mandibular size over time: it first narrows during a pre-adolescent ‘catch-up’ phase, then rapidly widens during the adolescent growth spurt. This divergence during adolescence quickly worsens the mandibular deficiency in Class II children.

Figure 3 shows the mean difference (and the 95% CI) in mandibular length between Class I and Class II from 6 to 20 years of age. The results indicate that, due to differences in the dynamics of the growth spurt, the difference in mandibular size (Class I–Class II) first decreases and then increases rapidly during the adolescent growth period. Since the adolescent growth spurt occurs earlier for males than for females, this transition (a decrease followed by an increase in the class difference in mandibular size) occurs at the age of 13.0 years [95% CI (12.4, 13.3)] for males, and 11.2 years [95% CI (10.7, 11.6)] for females.

### Growth parameters

On average, children with Class I growth pattern consistently exhibit a larger mandible (4–5 mm) than their Class II counterparts across both sexes (Table 2). The adolescent growth spurt for the Class I group is characterized by a slightly later onset (0.6 years) and a marginally higher peak intensity (0.2 mm/year) than Class II group. However, while these inter-class differences are statistically robust (Probability of Direction > 95%), ROPE analysis suggests they are not clinically meaningful. The mean differences for growth timing and intensity fall within our predefined ROPE intervals whereas the evidence regarding the overall size difference remains inconclusive (Table 2).

**Table 2.** Class differences (Class I − Class II) in population average growth parameters (size, timing, and intensity) and the corresponding test results for probability of direction (PD) and region of practical equivalence (ROPE) for males and females.

Sex	Parameter^a^	Class	Estimate	Difference	PD^d^ (%)	ROPE^e^ (%)	ROPE
			(95% CI)^b^	(95% CI)^c^			Decision^f^
Male	Size	Class I	115.6 (112.6, 118.9)	5.0 (3.0, 7.0)	100	49.2	Undecided
		Class II	110.6 (107.7, 113.7)				
	Timing	Class I	13.6 (13.3, 13.9)	0.6 (0.3, 0.9)	100	100	Accepted
		Class II	13.0 (12.7, 13.3)				
	Intensity	Class I	3.3 (3.1, 3.5)	0.2 (0.0, 0.4)	98.3	100	Accepted
		Class II	3.1 (2.9, 3.2)				
Female	Size	Class I	106.8 (104.1, 109.6)	4.4 (3.2, 5.7)	100	82.4	Undecided
		Class II	102.3 (99.7, 105.1)				
	Timing	Class I	11.8 (11.6, 12.1)	0.6 (0.2, 0.9)	99.9	100	Accepted
		Class II	11.2 (11.0, 11.5)				
	Intensity	Class I	3.0 (2.8, 3.2)	0.2 (−0.0, 0.4)	96.4	100	Accepted

^a^Parameter:

Size—jaw length (COPOD).

Timing—age at peak growth velocity.

Intensity—peak growth velocity.

^b^Estimates (95% CI): Mean and the 95% credible intervals.

^c^Difference (95% CI): Mean difference and the 95% credible intervals.

^d^PD: Probability of Direction (PD) for difference defines the certainty (in percentage) of strictly positive or negative direction of estimates (NULL = 0).

^e^ROPE: Region of Practical Equivalence (ROPE) for difference defines the probability of distribution (in percentage) within the ROPE Range (NULL = ROPE Range i.e. size ± 5.0, timing ± 1.0, intensity ± 1.0)

^f^ROPE Decision:

Accepted—The posterior distribution is completely inside the ROPE.

Rejected—The posterior distribution is completely outside the ROPE.

Undecided—The posterior distribution is partially inside/outside the ROPE.

### Individual-level variability in growth parameters

The individual-level variability measured using the standard deviation of random effects (Table 3) reveals that the Class I group exhibits marginally greater heterogeneity for all three growth parameters (size, timing, and intensity) than the Class II group across both sexes. This suggests a wider range of growth pattern expressions among children with Class I growth pattern. However, these class differences in individual-level variability are minor, lack statistical robustness (Probability of Direction < 95%), and fall entirely within our predefined ROPE intervals. This indicates that the overall consistency of growth patterns does not differ in a clinically meaningful way between Class I and Class II groups.

**Table 3.** Class differences (Class I–Class II) in the individual-level variability (standard deviation of random effects) of growth parameters (size, timing, and intensity) and the corresponding test results for probability of direction (PD) and region of practical equivalence (ROPE) for males and females.

Sex	Parameter^a^	Class	Estimate	Difference	PD^d^ (%)	ROPE^e^ (%)	ROPE
			(95% CI)^b^	(95% CI)^c^			Decision^f^
Male	Size	Class I	3.6 (2.8, 4.5)	0.3 (−0.8, 1.5)	69.8	100	Accepted
		Class II	3.3 (2.5, 4.2)				
	Timing	Class I	1.1 (0.9, 1.4)	0.2 (−0.2, 0.6)	82.5	100	Accepted
		Class II	0.9 (0.7, 1.2)				
	Intensity	Class I	0.2 (0.2, 0.3)	0.1 (−0.0, 0.1)	79.9	100	Accepted
		Class II	0.1 (0.1, 0.2)				
Female	Size	Class I	2.4 (1.9, 3.1)	0.3 (−0.5, 1.1)	76.2	100	Accepted
		Class II	2.1 (1.7, 2.7)				
	Timing	Class I	0.9 (0.7, 1.1)	0.2 (−0.1, 0.5)	85.0	100	Accepted
		Class II	0.7 (0.6, 1.0)				
	Intensity	Class I	0.2 (0.2, 0.3)	0.1 (−0.0, 0.1)	79.0	100	Accepted

^a^Parameter:

Size—jaw length (COPOD).

Timing—age at peak growth.

Intensity—peak growth velocity.

^b^Estimates (95% CI): Standard deviation along with the 95% credible intervals.

^c^Difference (95% CI): Standard deviation difference along with the 95% credible intervals.

^d^PD: Probability of Direction (PD) for difference defines the certainty (in percentage) of strictly positive or negative direction of estimates (NULL = 0).

^e^ROPE: Region of Practical Equivalence (ROPE) for difference defines the probability of distribution (in percentage) within the ROPE Range (NULL = ROPE Range i.e. size ± 5.0, timing ± 1.0, intensity ± 0.1).

^f^ROPE Decision:

Accepted—The posterior distribution is completely inside the ROPE.

Rejected—The posterior distribution is completely outside the ROPE.

Undecided—The posterior distribution is partially inside/outside the ROPE.

### Correlation between individual-level growth parameters

Finally, model-estimated correlations between growth parameters (Table 4) show a consistent negative correlation between the timing and intensity of the adolescent growth spurt for both classes and sexes. This indicates that children who mature early tend to experience a more intense growth spurt and vice versa. In contrast, correlations involving the size parameter are consistently weak and associated with considerable uncertainty, suggesting that the adulthood mandibular size is largely independent of the timing or intensity of their adolescent growth spurt. These findings suggest that the interplay between timing and intensity of the growth spurt serves as the primary natural compensatory mechanism that minimizes the size difference between early- and late-maturing children. For both sexes, a weaker correlation between timing and intensity for Class II than Class I indicates that this natural compensation is somewhat compromised, leading to a smaller mandibular size in Class II malocclusion (Table 4).

**Table 4.** Class-specific (Class I and Class II) correlation estimates of random effects (size, timing, and intensity) for males and females.

Sex	Class		Correlation (95% CI)^a^		
			Size	Timing	Intensity
Male	Class I	Size	1		
		Timing	0.2 (−0.1, 0.5)^b^	1	
		Intensity	0.2 (−0.1, 0.5)^c^	−0.6 (−0.8, −0.3)^d^	1
	Class II	Size	1		
		Timing	0.1 (−0.2, 0.4)^b^	1	
		Intensity	0.2 (−0.1, 0.5)^c^	−0.5 (−0.7, −0.2)^d^	1
Female	Class I	Size	1		
		Timing	0.3 (−0.0, 0.6)^b^	1	
		Intensity	0.1 (−0.2, 0.4)^c^	−0.5 (−0.7, −0.2)^d^	1
	Class II	Size	1		
		Timing	0.3 (−0.1, 0.6)^b^	1	
		Intensity	0.2 (−0.2, 0.5)^c^	−0.2 (−0.5, 0.2)^d^	1

^a^Correlation (95% CI): Correlation coefficient along with the 95% credible intervals for random effect parameters:

Size—jaw length (COPOD).

Timing—age at peak growth velocity.

Intensity—peak growth velocity.

^b^Correlation between size and timing parameters.

^c^Correlation between size and intensity parameters.

^d^Correlation between timing and intensity parameters.

## Discussion

Effective treatment planning for skeletal discrepancies like Class II malocclusion relies on a precise understanding of growth dynamics. This study applied a novel Bayesian SITAR model to characterize Class II mandibular development relative to the normative growth pattern observed in Class I children. The primary contribution of this work is its detailed analysis of the distinct roles timing and intensity of the adolescent growth spurt play in determining the adulthood mandibular size and their effect on the mandibular deficiency observed in Class II children. Furthermore, our use of a Bayesian framework allows for direct probabilistic statements about the clinical meaningfulness of these differences, a methodological advancement over prior research in this area.

It is well-established that the mandibular deficiency in Class II children originates early during the pre-adolescent phase [30, 31], and this initial deficit is then compounded by subsequent growth, leading to a more pronounced skeletal discrepancy in adulthood [30–36]. However, the precise mechanisms driving this discrepancy during the adolescent growth spurt are less understood. The present study addresses this gap by providing a dynamic characterization of the spurt itself. By modeling and comparing class differences in growth timing (age at peak velocity) and intensity (peak velocity), we explain how and when the final skeletal discrepancy is magnified during adolescence.

A key insight from our analysis is the varying strength of the negative correlation between the timing and intensity of the adolescent growth spurt. For Class I, the correlation is strong, suggesting a natural compensatory mechanism: children who mature earlier experience a less intense peak, while late maturing undergo a more intense spurt. This interplay appears to minimize size discrepancies arising from maturational variations. Crucially, this compensatory mechanism is substantially weaker in children with Class II malocclusion. These findings point to a potential etiological factor for the mandibular deficiency characteristic of Class II malocclusion. Consequently, a less robust natural growth response could make clinical intervention via growth modification more critical for achieving a normal skeletal outcome.

Further investigation reveals that the mandibular size discrepancy between Class I and Class II children does not progress linearly—a finding with important clinical implications. Although the overall mandibular deficiency in Class II children worsens with time, it temporarily improves during the pre-adolescent period before rapidly increasing again during the adolescent growth spurt. This complex growth dynamic offers a potential explanation for the inconsistent treatment outcomes and unpredictable relapse patterns reported for growth modification procedures [2, 37]. We hypothesize that the temporary ‘catch-up’ growth phase could correspond to periods where treatment appears highly effective, while the subsequent divergence corresponds to periods of apparent relapse. It is important to note that this is an interpretive extrapolation; validating this hypothesis would require prospective clinical trials directly linking our model's growth phases to treatment outcomes.

Lastly, this investigation highlights a key methodological consideration for the analysis of longitudinal growth data. The application of Bayesian framework enabled the successful estimation of the SITAR model with simultaneous inclusion of all three random effects—a crucial feature given the strong correlation observed between timing and intensity parameters. The exclusion of either random effect, a simplification sometimes necessitated by the convergence challenges of frequentist models [14], would likely result in biased estimates for the remaining correlated parameters [9, 38, 39]. The Bayesian paradigm, therefore, presents a robust and flexible alternative to frequentist models, particularly when convergence issues or limited data might otherwise compel the omission of random effects [40].

### Limitations

We acknowledge that our study is based on historical data from the AAOF Craniofacial Growth Legacy Collection (1930–85), and several considerations related to the use of such data warrant discussion [41]. First, the dataset comprises subset of children who participated in different growth studies. To mitigate this, we employed a three-level hierarchical model with study-level random effects to account for inter-study variation and reduce potential bias. Second, the cephalometric records span multiple centers, technologies, and operators over several decades. While mixed-effects modeling helps account for study-level variability, residual measurement heterogeneity may still influence outcomes. Third, the dataset predominantly includes children of European ancestry from North American populations, which limits the generalizability of our findings to more diverse contemporary populations. Lastly, previous research [42] has documented secular trends in craniofacial growth—such as earlier pubertal timing and changes in growth magnitude—which may further constrain the applicability of our results to present-day cohorts. Accordingly, clinical interpretations should be made cautiously, and future validation using modern, ethnically diverse longitudinal datasets will be essential to confirm the robustness and relevance of our findings.

We recognize that power analysis is a key element of prospective study design. However, given the retrospective design of our study, *a priori* power analysis was not possible. We avoided *post hoc* power calculations which are widely recognized as conceptually invalid and analytically misleading as they create a circular argument based on the observed effect size [43, 44]. Instead, we report Bayesian credible intervals for all estimates, as they provide a direct and more meaningful quantification of parameter uncertainty.

Another limitation of this study is that the sample sizes for the sex-specific subgroups were based on data availability, which precluded a formal *a priori* power analysis. Consequently, while our results on dimorphic growth are valuable, the findings should be interpreted with caution. To address this, future prospectively designed studies with larger, adequately powered cohorts are needed to validate these results.

Lastly, the study is limited by its use of a 2D metric (Co-Pg), which simplifies the complex, 3D reality of mandibular growth. Future research should therefore apply the SITAR model to 3D imaging data such as cone-beam computed tomography (CBCT) to provide a more comprehensive analysis of the spatial dynamics of sex- and class-specific growth patterns.

## Conclusion

The Class II growth pattern, characterized by a smaller mandibular size compared to Class I, is established early in development. However, due to differences in the timing and intensity of the growth spurt, the mandibular size discrepancy between Class I and Class II increases with age. For both sexes, the growth spurt is more intense and occurs later for Class I than for Class II, although the evidence remains inconclusive. Individual-level variability in growth dynamics highlights the importance of clinicians not relying solely on guidelines based on population averages. Instead, they should assess whether a patient's growth pattern is delayed, advanced, or within the normal range of variation. This approach enables more personalized treatment plans tailored to each patient's unique growth pattern.

## Supplementary Material

cjaf088_Supplementary_Data

## Data Availability

The AAOF Craniofacial Growth Legacy Collection is openly available in a public repository (www.aaoflegacycollection.org).
