# Supplementary material for: Studying the dynamics of mandibular growth spurts in individuals with Class I and Class II skeletal growth patterns using the Bayesian superimposition by translation and rotation (SITAR) model
Source: Eur J Orthod. 2025 Nov 11;47(6):cjaf088. doi: 10.1093/ejo/cjaf088 (PMC12603614; doi:10.1093/ejo/cjaf088)
Supplement: cjaf088_Supplementary_Data [file cjaf088_supplementary_data.pdf]

Supplementary File

"Studying the dynamics of mandibular growth spurts in individuals with Class I and Class II skeletal growth patterns using the Bayesian superimposition by translation and rotation (SITAR) model"

Section 1. Superimposition by Translation and Rotation (SITAR) model

The SITAR growth curve model fits a mean curve to the data, with the assumption that individual-specific growth patterns vary from the mean in three key ways (1): the size, which reflects a vertical shift relative to the mean curve; the timing of the adolescent growth spurt, indicated by a horizontal shift relative to the average age at peak growth velocity; and the intensity of growth velocity, which refers to the rate at which individuals grow during the adolescent growth spurt compared to the mean growth intensity (horizontal stretch). A schematic diagram showing the effect of size, timing and intensity on growth curve is shown in Figure S1.

We fit a three-level SITAR model that includes 'Class' as a covariate in the fixed effects to capture Class-specific growth parameters (size, timing, and intensity). The model incorporates individual-level (level 2) and study-level (level 3) random effects to account for both individual and study-specific differences in these growth parameters. To model distinct variance-covariance parameters for each group, the individual-specific random effects are estimated separately for Class I and Class II. Additionally, to address potential heteroskedasticity between the Class I and Class II groups, 'Class' is included as a covariate in the residual variance parameter.

A detailed description of the model follows below, outlining the likelihood, mean structure, and residuals in a logical sequence. Priors used to fit SITAR model to male and female data are shown in Section 2.

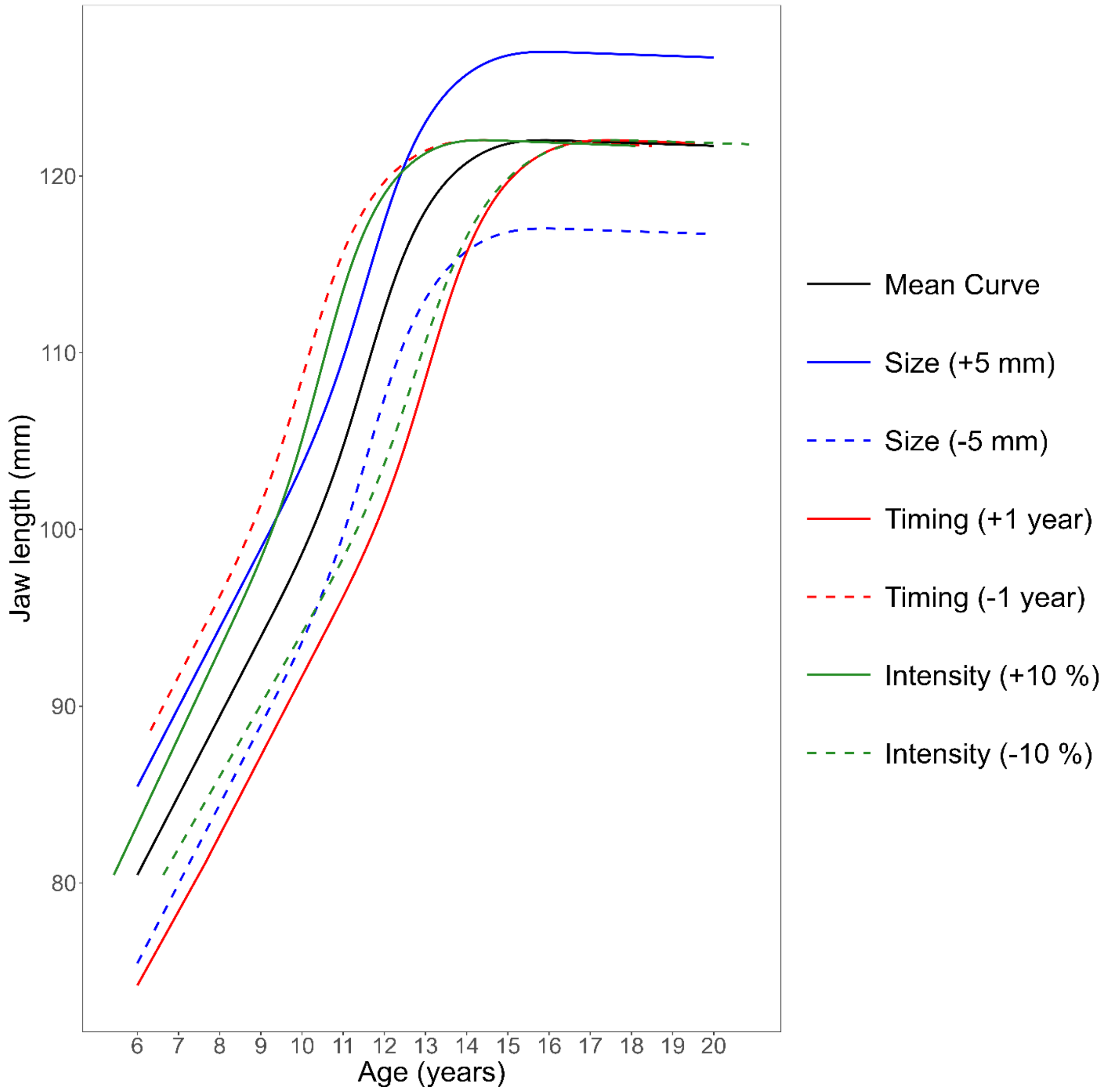

Figure S1 A schematic illustration depicting the effects of the SITAR model parameters on the growth curve. The black solid line represents the population mean curve. The colored lines show how growth curves are transformed by adjusting for size (vertical shift, blue), timing (horizontal shift, red), and intensity (stretching/shrinking of the age axis, green). Dashed lines indicate negative parameter values, whereas solid lines indicate positive values.

Likelihood

A three-level SITAR model is represented as follows:

$$y_{ijk} \sim Normal(\mu_{ijk}, \sigma_{ijk}) \qquad (1)$$

where y_{ijk} denotes the jaw growth measurement collected at occasion i (i = 1, 2, 3, …, n_i) for individual j (j = 1, 2, 3, …, J) belonging to the k-th growth study (k = 1, 2, 3, …, K). The $Normal(\mu_{ijk}, \sigma_{ijk})$ represents the likelihood, which states that the outcome is normally distributed with mean μ_{ijk} and residuals, σ_{ijk}. The σ_{ijk} denotes the residual variation not accounted for by the mean structure and can vary across time, individuals, and growth studies.

Mean structure (μ), including priors

The μ is a function of spline coefficients and growth parameters (size, timing, and intensity), including the covariate effects, and is expressed as follows:

$$\mu_{ijk} = \alpha_0 + X_{ijk}\alpha_{ClassII} + \alpha_j + \alpha_k + \sum_{r=1}^{p-1} \beta_r \mathbf{Spl}\left(\frac{x_{ijk} - \overline{x_{ijk}} - (\zeta_0 + X_{ijk}\zeta_{ClassII} + \zeta_j + \zeta_k)}{e^{-(\gamma_0 + X_{ijk}\gamma_{ClassII} + \gamma_j + \gamma_k)}}\right) \qquad (2)$$

where **Spl(.)** is the natural cubic spline function that generates the spline design matrix for a given number of knots, p. The x_{ijk} represents the age in years at the i-th occasion for individual j in the k-th growth study, and is mean-centered by subtracting the mean age, denoted as $\overline{x_{ijk}}$ where $\overline{x_{ijk}} = \frac{1}{n}\sum_{i=1}^{n} x_i$. The mean-centered predictor variable indicates that the origin of the timing parameter is set at the mean value of the predictor. The X_{ijk} is a dummy variable defined as:

$$X_{ijk} = \begin{cases} 1, & Class = Class\ II \\ 0, & \text{otherwise} \end{cases}$$

The X_{ijk} captures Class-specific differences in the growth parameters. Class I is used as the reference category, meaning the intercept corresponds to the Class I coefficients. Since 'Class' is a factor variable with two levels (Class I and Class II), only one dummy variable is created that captures the difference between the Class I (i.e., intercept) and Class II groups.

Population parameters

The population parameters include the spline regression coefficients $\beta_1, \dots, \beta_r$, which define the mean growth curve, as well as the growth coefficients for Class I: size (α_0), timing (ζ_0), and intensity (γ_0). The differences in growth coefficients between Class I and Class II are represented by $\alpha_{ClassII}$, $\zeta_{ClassII}$, and $\gamma_{ClassII}$ for size, timing, and intensity, respectively. All population parameters are assigned normal distribution-based priors, as shown below:

$$\beta_1,..,\beta_r \sim Normal\left(\beta_{r\_lm}, \boldsymbol{X}_{Spl} \cdot 2.5\right)$$

$$\alpha_0 \sim Normal(y_{mean}, y_{sd} \cdot 2.5)$$

$$\zeta_0 \sim Normal(0, 2.0)$$

$$\gamma_0 \sim Normal(0, 1.0)$$

$$\alpha_{ClassII} \sim Normal(0, 5.0)$$

$$\zeta_{ClassII} \sim Normal(0, 1.0)$$

$$\gamma_{ClassII} \sim Normal(0, 0.2)$$

Where y_{mean} is the mean of the outcome variable (jaw length), y_{sd} is the standard deviation of the outcome variable (jaw length), and β_{r_lm} denotes the vector of spline coefficient ($\beta_1, \dots, \beta_{r\_lm},$) obtained from the linear model fit to the data, as shown below in Eq (3):

$$y_{ijk} = \alpha_{0\_lm} + X_{ijk}\alpha_{ClassII\_lm} + \sum_{r=1}^{p-1} \beta_{r\_lm} \mathbf{Spl}\left(x_{ijk} - \overline{x_{ijk}}\right) \quad (3)$$

The X_{Spl} is $\frac{y_{sd}}{Spl_{sd}}$ where Spl_{sd} is the standard deviation of the spline design matrix constructed during the linear model fit to the data. The approach used here to scale priors for regression coefficients i.e., $y_{sd} \cdot 2.5$ and $X_{Spl} \cdot 2.5$ is same as followed in *bsitar* (2) and popular Bayesian packages such as *rstanarm (3)* and *brms (4)*.

Random effects parameter

The individual-specific random effects for size (α_j), timing (ζ_j), and intensity (γ_j) describe how an individual's growth trajectory differs from the mean growth curve. Similarly, growth study-specific random effects for size (α_k), timing (ζ_k), and intensity (γ_k) capture study-specific deviations in the growth trajectory. The random effects are assumed to follow a multivariate normal distribution with zero means and an unstructured variance-covariance matrix (i.e., distinct variances and co-variance between random effects) for both individual-specific ($\boldsymbol{\Sigma_{ID}}$) and study-specific ($\boldsymbol{\Sigma_{study}}$) random effects.

Individual-specific random effects

The individual-specific variance-covariance parameters are estimated separately for Class I and Class II, as shown below. The unstructured variance-covariance matrix $\mathbf{\Sigma_{ID}}$ is constructed using the separation strategy. In this approach, which is followed by Stan (5) and implemented in *bsitar* (2) and other popular packages *(3, 4)*, the variance-covariance matrix **S** (composed of the standard deviation diagonal vector), and a correlation matrix, C. The priors for the standard deviation parameters are specified using a half-normal distribution ($Normal_{half}$) with lower bounds at zero, ensuring that the standard deviations are positive. The prior for the correlations follows the Lewandowski-Kurowicka-Joe (LKJ) distribution, specified via a single parameter, η. When $\eta = 1$ (the default), all correlation matrices are equally likely a priori. If $\eta > 1$, extreme correlations

become less likely, whereas if $0 < \eta < 1$, higher probabilities are assigned to extreme correlations.

$$\begin{bmatrix} \alpha_j^{\text{ClassI}} \\ \zeta_j^{\text{ClassI}} \\ \gamma_j^{\text{ClassI}} \\ \alpha_j^{\text{ClassII}} \\ \zeta_j^{\text{ClassII}} \\ \gamma_j^{\text{ClassII}} \end{bmatrix} \sim \text{MVNormal}\left(\begin{bmatrix} 0 \\ 0 \\ 0 \\ 0 \\ 0 \\ 0 \end{bmatrix}, \mathbf{\Sigma_{ID}}\right)$$

$$\mathbf{\Sigma_{ID}} = \text{diag}(\mathbf{S}_{ID})\mathbf{C}_{ID}diag(\mathbf{S}_{ID})$$

$$\mathbf{S}_{ID} = \begin{bmatrix} \alpha_j^{\text{ClassI}} & 0 & 0 & 0 & 0 & 0 \\ 0 & \zeta_j^{\text{ClassI}} & 0 & 0 & 0 & 0 \\ 0 & 0 & \gamma_j^{\text{ClassI}} & 0 & 0 & 0 \\ 0 & 0 & 0 & \alpha_j^{\text{ClassII}} & 0 & 0 \\ 0 & 0 & 0 & 0 & \zeta_j^{\text{ClassII}} & 0 \\ 0 & 0 & 0 & 0 & 0 & \gamma_j^{\text{ClassII}} \end{bmatrix}$$

$$\mathbf{C}_{ID} = \begin{bmatrix} 1 & \rho_{\alpha_j^{\text{ClassI}}\zeta_j^{\text{ClassI}}} & \rho_{\alpha_j^{\text{ClassI}}\gamma_j^{\text{ClassI}}} & 0 & 0 & 0 \\ \rho_{\zeta_j^{\text{ClassI}}\alpha_j^{\text{ClassI}}} & 1 & \rho_{\zeta_j^{\text{ClassI}}\gamma_j^{\text{ClassI}}} & 0 & 0 & 0 \\ \rho_{\gamma_j^{\text{ClassI}}\alpha_j^{\text{ClassI}}} & \rho_{\gamma_j^{\text{ClassI}}\zeta_j^{\text{ClassI}}} & 1 & 0 & 0 & 0 \\ 0 & 0 & 0 & 1 & \rho_{\alpha_j^{\text{ClassII}}\zeta_j^{\text{ClassII}}} & \rho_{\alpha_j^{\text{ClassII}}\gamma_j^{\text{ClassII}}} \\ 0 & 0 & 0 & \rho_{\zeta_j^{\text{ClassII}}\alpha_j^{\text{ClassII}}} & 1 & \rho_{\zeta_j^{\text{ClassII}}\gamma_j^{\text{ClassII}}} \\ 0 & 0 & 0 & \rho_{\gamma_j^{\text{ClassII}}\alpha_j^{\text{ClassII}}} & \rho_{\gamma_j^{\text{ClassII}}\zeta_j^{\text{ClassII}}} & 1 \end{bmatrix}$$

$$\alpha_j^{\text{ClassI}} \sim normal_{half}(0,\ y_{sd})$$

$$\zeta_j^{\text{ClassI}} \sim normal_{half}(0,\ 2.0)$$

$$\gamma_j^{\text{ClassI}} \sim normal_{half}(0,\ 1.0)$$

$$\alpha_j^{\text{ClassII}} \sim normal_{half}(0,\ y_{sd})$$

$$\zeta_j^{\text{ClassII}} \sim normal_{half}(0,\ 2.0)$$

$$\gamma_j^{\text{ClassII}} \sim normal_{half}(0,\ 1.0)$$

$$C_{ID} \sim LKJ(1)$$

Study-specific random effects

Like the individual-specific variance-covariance matrix (see Section on Individual-specific random effects), the study-specific unstructured variance-covariance matrix $\boldsymbol{\Sigma}_{\mathbf{study}}$ is also constructed using the separation strategy. The priors for the standard deviation parameters are specified using a half-normal distribution with lower bounds at zero, while the LKJ prior is assigned to the correlation parameters.

$$\begin{bmatrix} \alpha_k \\ \zeta_k \\ \gamma_k \end{bmatrix} \sim MVNormal\left(\begin{bmatrix} 0 \\ 0 \\ 0 \end{bmatrix}, \boldsymbol{\Sigma}_{study} \right)$$

$$\boldsymbol{\Sigma}_{study} = diag(\boldsymbol{S}_{study})\boldsymbol{C}_{study}diag(S_{study})$$

$$S_{study} = \begin{bmatrix} \sigma_{\alpha_k} & 0 & 0 \\ 0 & \sigma_{\zeta_k} & 0 \\ 0 & 0 & \sigma_{\gamma_k} \end{bmatrix}$$

$$C_{study} = \begin{bmatrix} 1 & \rho_{\alpha_k\zeta_k} & \rho_{\alpha_k\gamma_k} \\ \rho_{\zeta_k\alpha_k} & 1 & \rho_{\zeta_k\gamma_k} \\ \rho_{\gamma_k\alpha_k} & \rho_{\gamma_k\zeta_k} & 1 \end{bmatrix}$$

$$\alpha_k \sim normal_{half}(0,\ 5.0)$$

$$\zeta_k \sim normal_{half}(0,\ 0.5)$$

$$\gamma_k \sim normal_{half}(0,\ 0.2)$$

$$C_{study} \sim LKJ(1)$$

Residual structure (σ), including priors

$$\log(\sigma_{ijk}) = \nu_0 + X_{ijk}\nu_{ClassII} \tag{4}$$

where $\log(\sigma_{ijk})$ shows that residual variance is modeled as a log-transformation, ensuring that the variance remains positive. The X_{ijk} is a dummy variable defined as:

$$X_{ijk} = \begin{cases} 1, & Class\ =\ Class\ II \\ 0, & \text{otherwise} \end{cases}$$

The variable X_{ijk} is included for the Class variable, capturing Class differences in the residual variance parameters. Equation (4) shows that the residual variance is now decomposed into two components: the Class I variance, denoted by the intercept ν_0, and the Class II variance, represented by $\nu_{ClassII}$. Class I is used as the reference category. The priors for these parameters are as follows:

$$\nu_0 \sim Normal(0,\ 1.0)$$

$$\nu_{ClassII} \sim Normal(0,\ 1.0)$$

Note that the priors are specified on the log scale.

Section 2. Data

We analyze the mandibular growth data (Condyle to Pogonion distance, COPOD) available from the American Association of Orthodontists Foundation (AAOF) Craniofacial Growth Legacy Collection database. The AAOF Craniofacial Growth Legacy Collection comprises data pooled from nine historical growth studies (Bolton, Burlington, Denver, Fels, Forsyth, Iowa, Mathews, Michigan, and Oregon) conducted between 1930 and 1985 (18). Children included in these studies were predominantly Anglo-Saxon or Caucasian. For further details, please refer to the Methodology Section of the main text.

Below we show growth trajectories for Class I and Class II individuals across both sexes (Figure S2). A detailed description of class- and study-specific sample characteristics for both sexes is provided in Table S1.

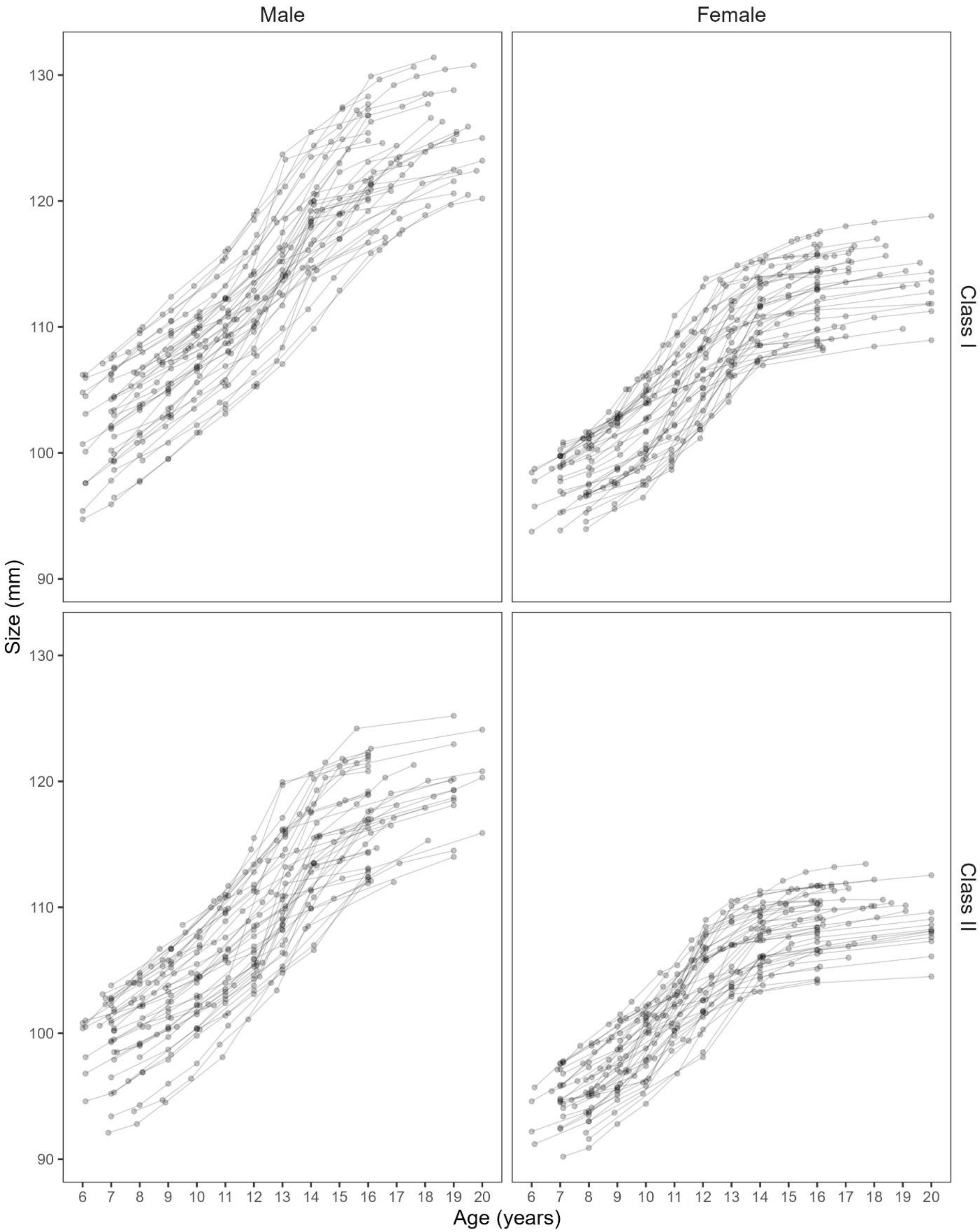

Figure S2 Observed growth trajectories for males and females.

Tables S1 Growth study specific summary of data analyzed in the study.

Sex	Study	Class	N [a]	Obs [b]	Age (mean) [c]	Age (SD) [d]	Age (range) [e]
Male	Bolton	Class I	6	46	11.3	3.2	7 to 19
	Bolton	Class II	9	74	12.1	3.3	7 to 20
	Burlington	Class I	5	38	12.2	3.8	6 to 20
	Burlington	Class II	8	68	11.6	3.3	6 to 20
	Denver	Class I	6	48	12.4	3.5	7 to 20
	Denver	Class II	5	45	12.1	3.3	7 to 19
	Mathews	Class I	9	78	12.4	3.4	6 to 20
	Mathews	Class II	3	29	11.4	3.1	7 to 16
	Michigan	Class I	10	101	11.7	3.6	6 to 20
	Michigan	Class II	7	69	11.6	3.4	6 to 19
	Oregon	Class I	4	54	12.8	4.0	6 to 20
	Oregon	Class II	8	67	12.2	3.5	6 to 20
Female	Bolton	Class I	5	42	12.2	3.4	7 to 20
	Bolton	Class II	5	46	11.7	3.5	7 to 20
	Burlington	Class I	6	50	11.6	3.5	6 to 20
	Burlington	Class II	9	70	12.2	3.8	6 to 20
	Denver	Class I	18	129	12.4	3.3	7 to 20
	Denver	Class II	9	69	12.0	3.3	7 to 20
	Mathews	Class I	4	39	11.7	3.0	7 to 16
	Mathews	Class II	7	70	12.4	3.2	7 to 19
	Michigan	Class I	3	35	12.0	3.8	6 to 20
	Michigan	Class II	4	42	12.1	3.6	6 to 18
	Oregon	Class I	4	50	12.3	3.8	6 to 20
	Oregon	Class II	6	48	12.4	3.3	7 to 20

[a] Number of Individuals, [b] Observations, [c] Mean age, [d] Standard deviation of age. [e] Age range

Section 3. Priors

Tables S2 A summary of priors used to fit the Bayesian SITAR model for males.

Class [a]	Parameter [b]	Coefficient [c]	Prior distribution [d]	95% CI [e]	99.99% CI [f]
b	a	Intercept	normal(113.5, 20.4)	73.52, 153.48	34.13, 192.87
	a	Class II	normal(0, 5)	-9.80, 9.80	-19.45, 19.45
	b	Intercept	normal(0, 2)	-3.92, 3.92	-7.78, 7.78
	b	Class II	normal(0, 1)	-1.96, 1.96	-3.89, 3.89
	c	Intercept	normal(0, 1)	-1.96, 1.96	-3.89, 3.89
	c	Class II	normal(0, 0.2)	-0.39, 0.39	-0.78, 0.78
	s1	Intercept	normal(1.5, 1.2)	-0.85, 3.85	-3.17, 6.17
	s2	Intercept	normal(0.1, 1.5)	-2.84, 3.04	-5.74, 5.94
	s3	Intercept	normal(12.4, 3.7)	5.15, 19.65	-2.00, 26.80
	s4	Intercept	normal(-28.5, 8.7)	-45.55, -11.45	-62.35, 5.35
	s5	Intercept	normal(4.4, 27)	-48.52, 57.32	-100.65, 109.45
sd	a	Intercept (id)	normal(0, 20.4)[0, Inf]	0.64, 45.72	0.00, 82.73
	b	Intercept (id)	normal(0, 2)[0, Inf]	0.06, 4.48	0.00, 8.11
	c	Intercept (id)	normal(0, 1)[0, Inf]	0.03, 2.24	0.00, 4.06
	a	Intercept (study)	normal(0, 5)[0, Inf]	0.16, 11.21	0.00, 20.28
	b	Intercept (study)	normal(0, 0.5)[0, Inf]	0.02, 1.12	0.00, 2.03
	c	Intercept (study)	normal(0, 0.2)[0, Inf]	0.01, 0.45	0.00, 0.81
rsd	sigma	Class I	normal(0, 1)	-1.96, 1.96	-3.89, 3.89
	sigma	Class II	normal(0, 1)	-1.96, 1.96	-3.89, 3.89

[a] Class: b - regression parameters; sd - standard deviation for random effects; rsd - residual standard deviation

[b] Parameter: a - size; b - timing; c - intensity; sigma - within individual variability

[c] Coefficient: For Class b, the Intercept represents Class I estimate whereas Class II denotes the difference between Class I and Class II. The s parameters (s1, s2,..) are splines coefficients; For Class sd, the Intercept is standard deviation of random effects for the group enclosed in the parentheses (id/study); For Class rsd, the Class I and Class II denote the within individual standard deviation estimates

[d] Prior distribution: Each coefficient is assigned normal distribution with mean and standard deviation specified in the parentheses. The square brackets with [0, Inf] indicates that distribution is half normal. For parameter 'c', the coefficient is exponentiated (see 95% CI mass below).

[e] 95% credible intervals mass for the estimates. For parameter 'c', the coefficient is exponentiated. For example, prior specified as 'normal (0, 1)' with 95% CI mass -1.96 between 1.96 is translated into exp(-1.96, 1.96) i.e., 0.14, 7.09

[f] 99.99% credible intervals mass for the estimates. See also 95% CI above)

Tables S3 A summary of priors used to fit the Bayesian SITAR model for females.

Class [a]	Parameter [b]	Coefficient [c]	Prior distribution [d]	95% CI [e]	99.99% CI [f]
b	a	Intercept	normal(101.2, 15.5)	70.82, 131.58	40.90, 161.50
	a	Class II	normal(0, 5)	-9.80, 9.80	-19.45, 19.45
	b	Intercept	normal(0, 2)	-3.92, 3.92	-7.78, 7.78
	b	Class II	normal(0, 1)	-1.96, 1.96	-3.89, 3.89
	c	Intercept	normal(0, 1)	-1.96, 1.96	-3.89, 3.89
	c	Class II	normal(0, 0.2)	-0.39, 0.39	-0.78, 0.78
	s1	Intercept	normal(0.6, 0.9)	-1.16, 2.36	-2.90, 4.10
	s2	Intercept	normal(6.6, 1.1)	4.44, 8.76	2.32, 10.88
	s3	Intercept	normal(-18.8, 3.2)	-25.07, -12.53	-31.25, -6.35
	s4	Intercept	normal(8.6, 11.8)	-14.53, 31.73	-37.31, 54.51
sd	a	Intercept (id)	normal(0, 15.5)[0, Inf]	0.49, 34.74	0.00, 62.86
	b	Intercept (id)	normal(0, 2)[0, Inf]	0.06, 4.48	0.00, 8.11
	c	Intercept (id)	normal(0, 1)[0, Inf]	0.03, 2.24	0.00, 4.06
	a	Intercept (study)	normal(0, 5)[0, Inf]	0.16, 11.21	0.00, 20.28
	b	Intercept (study)	normal(0, 0.5)[0, Inf]	0.02, 1.12	0.00, 2.03
	c	Intercept (study)	normal(0, 0.2)[0, Inf]	0.01, 0.45	0.00, 0.81
rsd	sigma	Class I	normal(0, 1)	-1.96, 1.96	-3.89, 3.89
	sigma	Class II	normal(0, 1)	-1.96, 1.96	-3.89, 3.89

[a] Class: b - regression parameters; sd - standard deviation for random effects; rsd - residual standard deviation

[b] Parameter: a - size; b - timing; c - intensity; sigma - within individual variability

[c] Coefficient: For Class b, the Intercept represents Class I estimate whereas Class II denotes the difference between Class I and Class II. The s parameters (s1, s2,..) are splines coefficients; For Class sd, the Intercept is standard deviation of random effects for the group enclosed in the parentheses (id/study); For Class rsd, the Class I and Class II denote the within individual standard deviation estimates

[d] Prior distribution: Each coefficient is assigned normal distribution with mean and standard deviation specified in the parentheses. The square brackets with [0, Inf] indicates that distribution is half normal. For parameter 'c', the coefficient is exponentiated (see 95% CI mass below).

[e] 95% credible intervals mass for the estimates. For parameter 'c', the coefficient is exponentiated. For example, prior specified as 'normal (0, 1)' with 95% CI mass -1.96 between 1.96 is translated into exp(-1.96, 1.96) i.e., 0.14, 7.09

[f] 99.99% credible intervals mass for the estimates. See also 95% CI above)

Section 4. Prior sensitivity analysis

Table S3 Prior sensitivity analysis results for males.

Class [a]	Parameter [b]	Coefficient [c]	Prior [d]	Likelihood [e]	Diagnosis [f]
b	a	Intercept	0.02	0.10	no-conflict
	a	Class II	0.02	0.08	no-conflict
	b	Intercept	0.04	0.07	no-conflict
	b	Class II	0.01	0.10	no-conflict
	c	Intercept	0.04	0.14	no-conflict
	c	Class II	0.01	0.09	no-conflict
	s1	Intercept	0.02	0.17	no-conflict
	s2	Intercept	0.04	0.07	no-conflict
	s3	Intercept	0.03	0.08	no-conflict
	s4	Intercept	0.04	0.08	no-conflict
	s5	Intercept	0.04	0.14	no-conflict
sd	a	Intercept (id)	0.01	0.09	no-conflict
	b	Intercept (id)	0.02	0.10	no-conflict
	c	Intercept (id)	0.02	0.09	no-conflict
	a	Intercept (study)	0.03	0.12	no-conflict
	b	Intercept (study)	0.04	0.08	no-conflict
	c	Intercept (study)	0.03	0.08	no-conflict
rsd	sigma	Class I	0.01	0.44	no-conflict
	sigma	Class II	0.00	0.49	no-conflict

[a] Class: b - regression parameters; sd - standard deviation for random effects; rsd - residual standard deviation

[b] Parameter: a - size; b - timing; c - intensity; sigma - within individual variability

[c] Coefficient: For Class b, the Intercept represents Class I estimate whereas Class II denotes the difference between Class I and Class II. The s parameters (s1, s2,..) are splines coefficients; For Class sd, the Intercept is standard deviation of random effects for the group enclosed in the parentheses (id/study); For Class rsd, the Class I and Class II denote the within individual standard deviation estimates

[d] Prior: prior variability resulting from the power-scaling perturbation

[e] Likelihood: likelihood informativity after power-scaling perturbation

[f] Diagnosis: Diagnostic test can indicate the presence of prior-data conflict and/or the prior-likelihood conflict at a given threshold (0.05).The diagnostic result is presented as a string as shown follows:

'no conflict' indicates no 'prior-data' or 'prior-likelihood' conflict

'prior-data conflict' indicates that the prior probability varies more than the threshold (Prior > 0.05)

'prior-likelihood conflict' shows that likelihood informativity drops below the threshold (Likelihood < 0.05)

Table S4 Prior sensitivity analysis results for females.

Class [a]	Parameter [b]	Coefficient [c]	Prior [d]	Likelihood [e]	Diagnosis [f]
b	a	Intercept	0.01	0.07	no-conflict
	a	Class II	0.01	0.13	no-conflict
	b	Intercept	0.01	0.05	no-conflict
	b	Class II	0.01	0.08	no-conflict
	c	Intercept	0.03	0.17	no-conflict
	c	Class II	0.01	0.04	no-conflict
	s1	Intercept	0.02	0.17	no-conflict
	s2	Intercept	0.04	0.12	no-conflict
	s3	Intercept	0.04	0.11	no-conflict
	s4	Intercept	0.04	0.10	no-conflict
sd	a	Intercept (id)	0.01	0.08	no-conflict
	b	Intercept (id)	0.01	0.09	no-conflict
	c	Intercept (id)	0.00	0.09	no-conflict
	a	Intercept (study)	0.03	0.11	no-conflict
	b	Intercept (study)	0.03	0.12	no-conflict
	c	Intercept (study)	0.03	0.30	no-conflict
rsd	sigma	Class I	0.01	0.50	no-conflict
	sigma	Class II	0.00	0.49	no-conflict

[a] Class: b - regression parameters; sd - standard deviation for random effects; rsd - residual standard deviation

[b] Parameter: a - size; b - timing; c - intensity; sigma - within individual variability

[c] Coefficient: For Class b, the Intercept represents Class I estimate whereas Class II denotes the difference between Class I and Class II. The s parameters (s1, s2,..) are splines coefficients; For Class sd, the Intercept is standard deviation of random effects for the group enclosed in the parentheses (id/study); For Class rsd, the Class I and Class II denote the within individual standard deviation estimates

[d] Prior: prior variability resulting from the power-scaling perturbation

[e] Likelihood: likelihood informativity after power-scaling perturbation

[f] Diagnosis: Diagnostic test can indicate the presence of prior-data conflict and/or the prior-likelihood conflict at a given threshold (0.05).The diagnostic result is presented as a string as shown follows:

'no conflict' indicates no 'prior-data' or 'prior-likelihood' conflict

'prior-data conflict' indicates that the prior probability varies more than the threshold (Prior > 0.05)

'prior-likelihood conflict' shows that likelihood informativity drops below the threshold (Likelihood < 0.05)

Section 5. Posterior predictive check

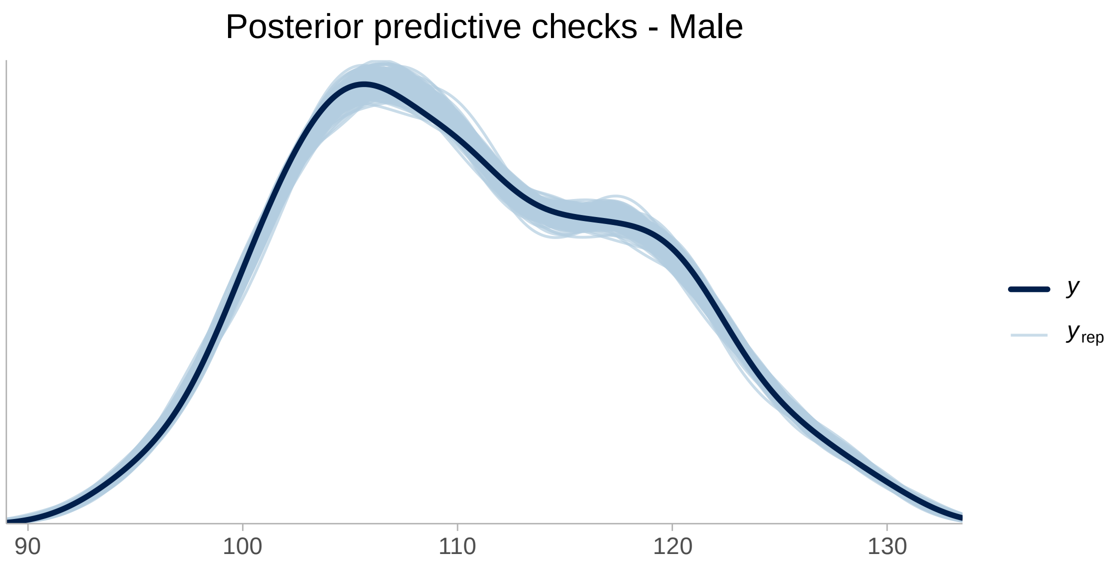

Figure S3 Posterior predictive check for male data.

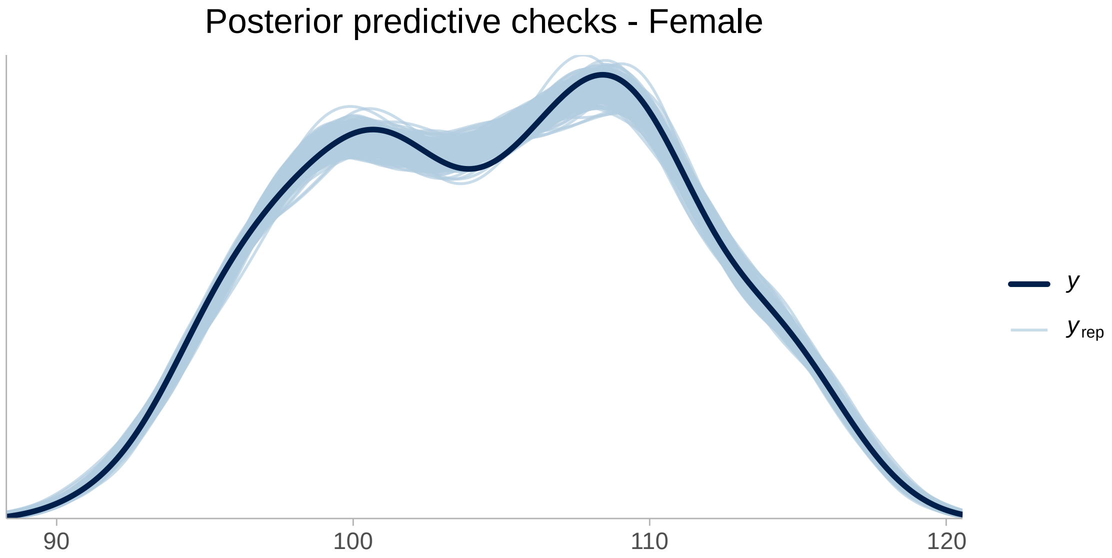

Figure S4 Posterior predictive check for female data.

Section 6. Residual plots

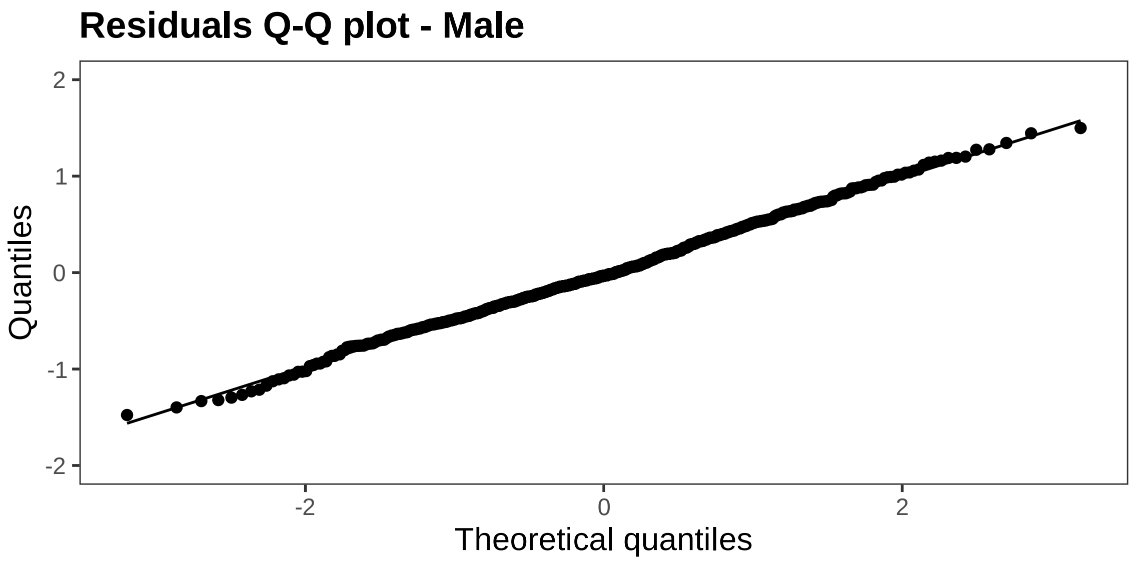

Figure S5 Residual plot to assess normality assumption for male data.

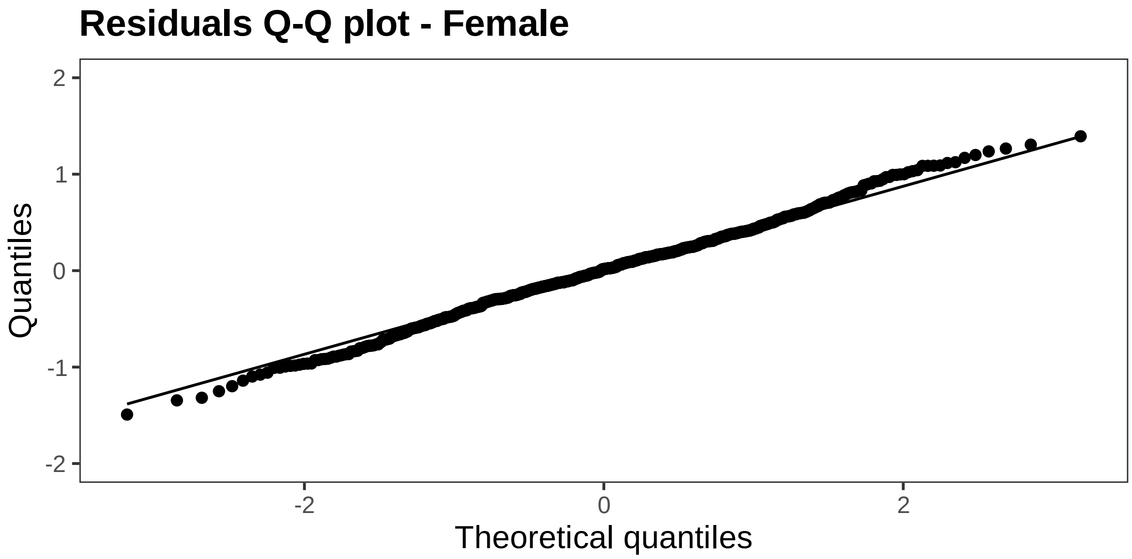

Figure S6 Residual plot to assess normality assumption for female data.

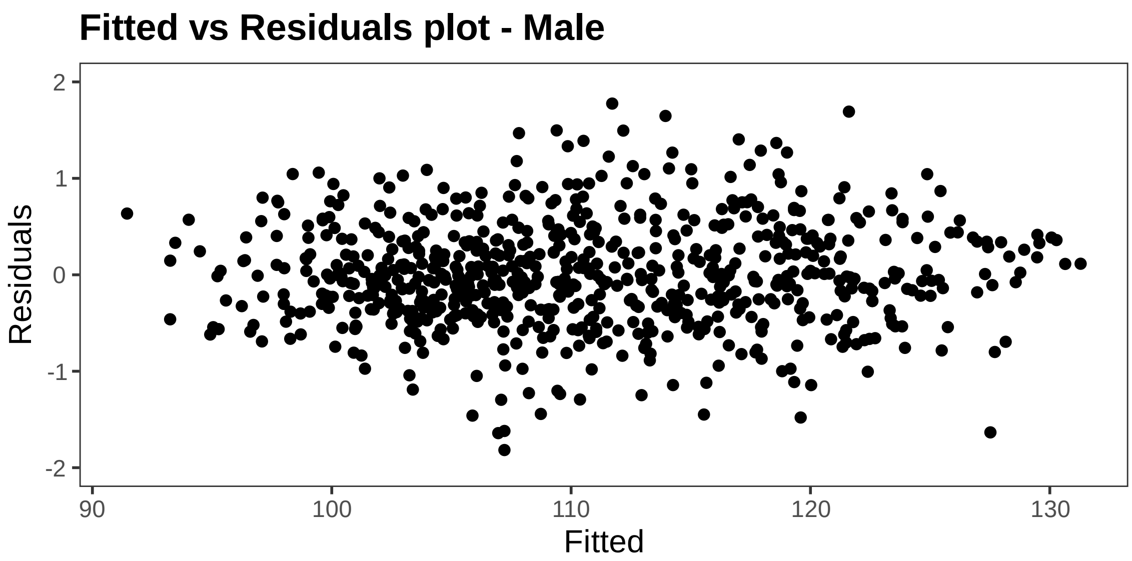

Figure S7 Residual versus fitted plot for male data.

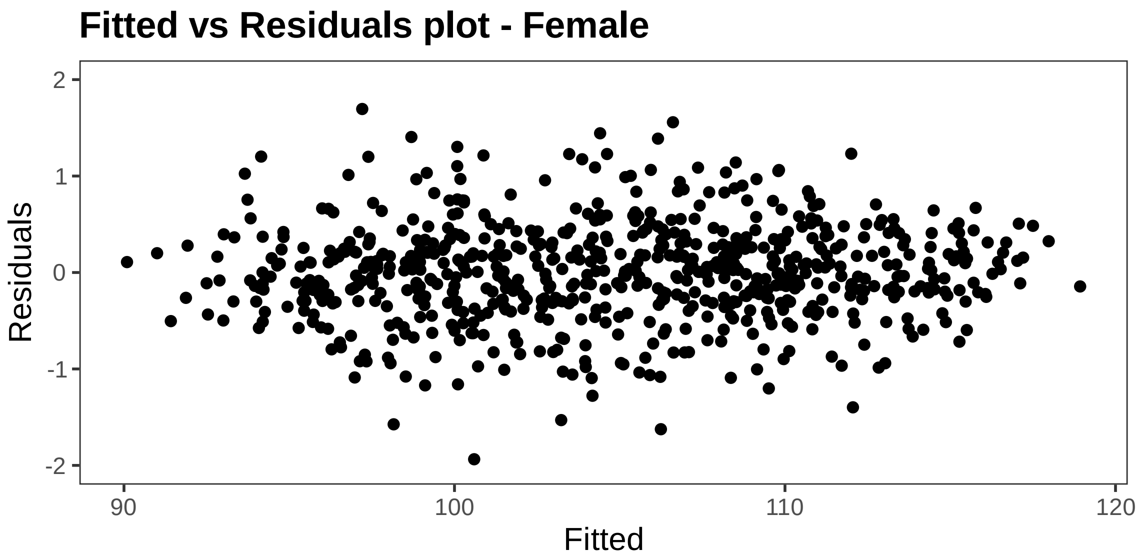

Figure

Figure S8 Residual versus fitted plot for female data.

Section 7. MCMC diagnostic plots

MCMC diagnostic plots for males

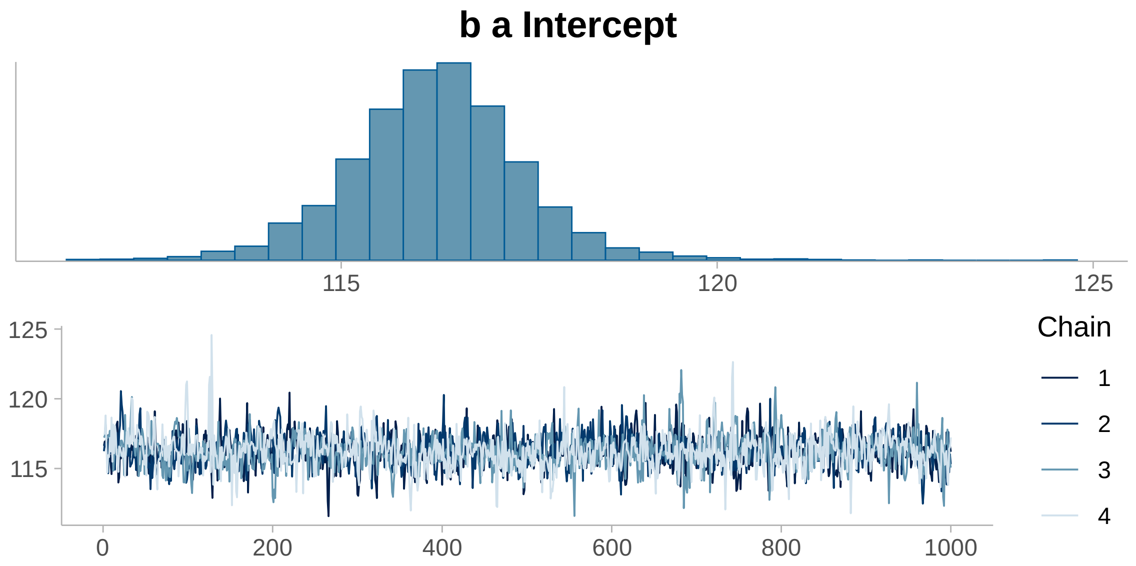

b a Class II

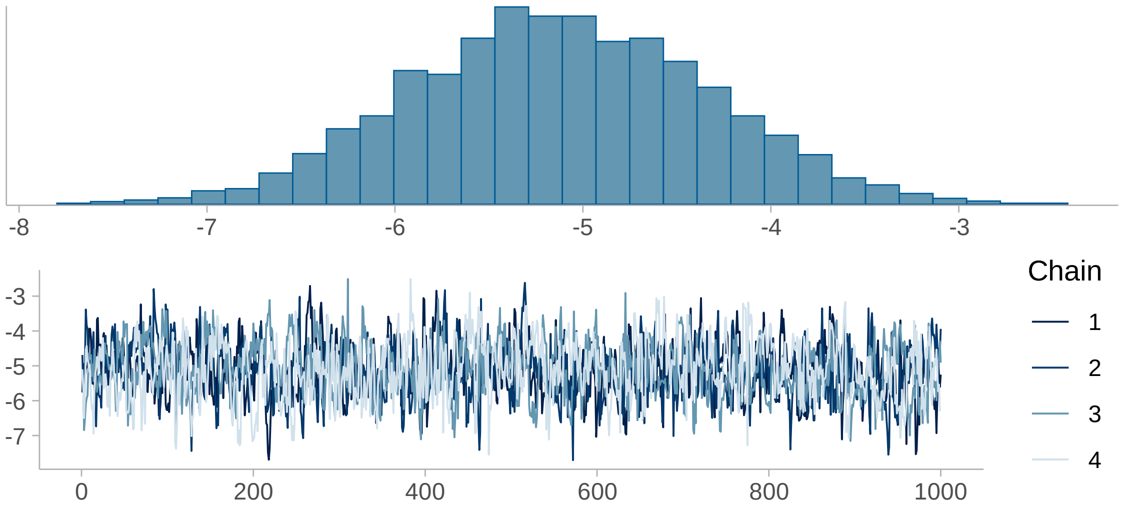

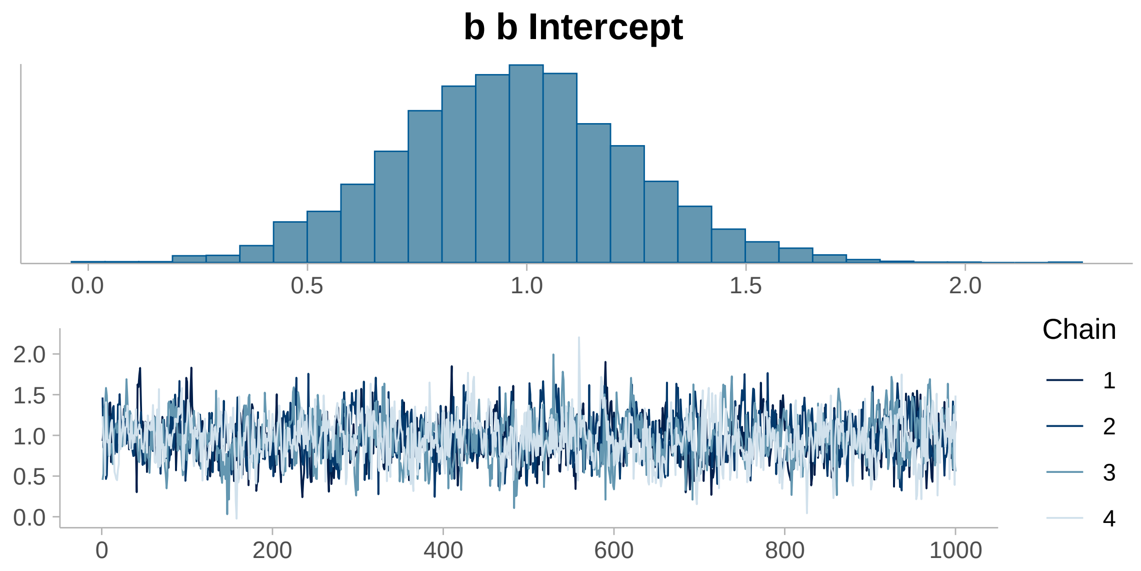
b b Intercept
0.0
0.5
1.0
1.5
2.0
Chain
1
2
3
4
0
200
400
600
800
1000

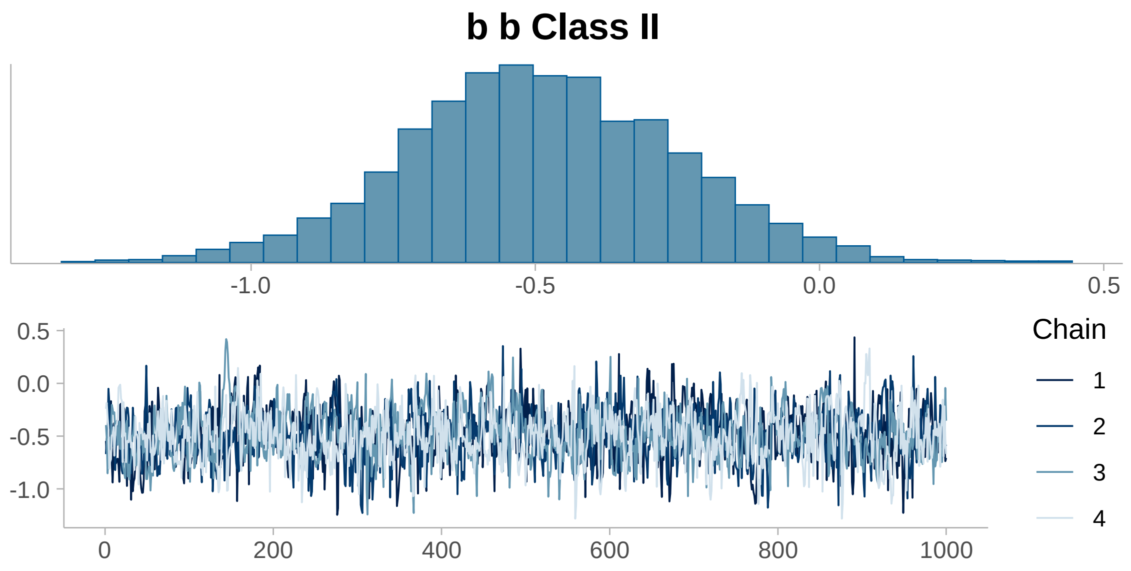
b b Class II
-1.0
-0.5
0.0
0.5
Chain
1
2
3
4
0
200
400
600
800
1000

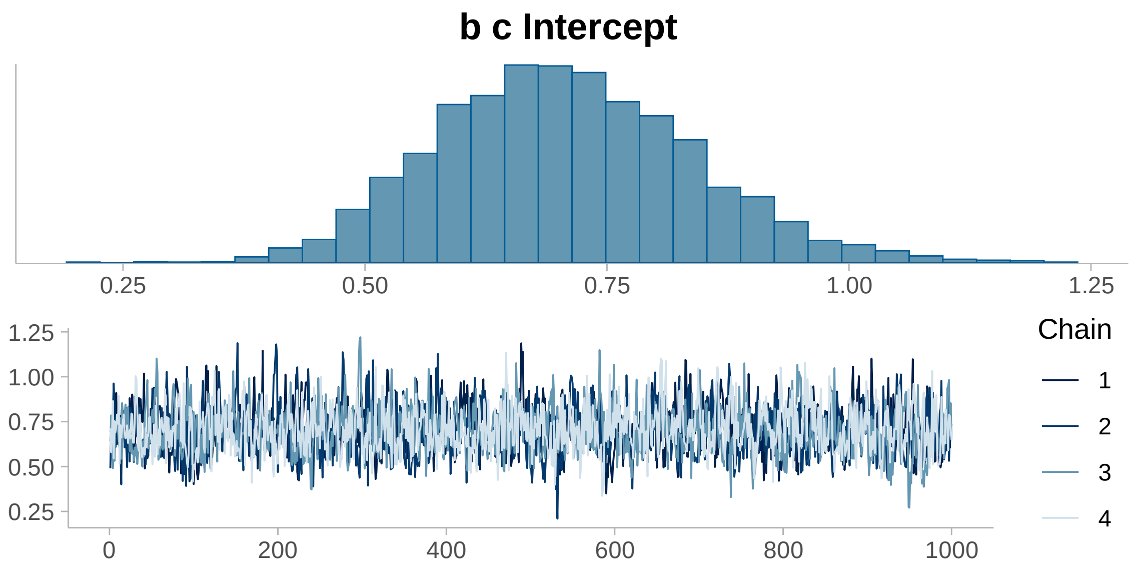
b c Intercept
0.25
0.50
0.75
1.00
1.25
Chain
1
2
3
4
0
200
400
600
800
1000

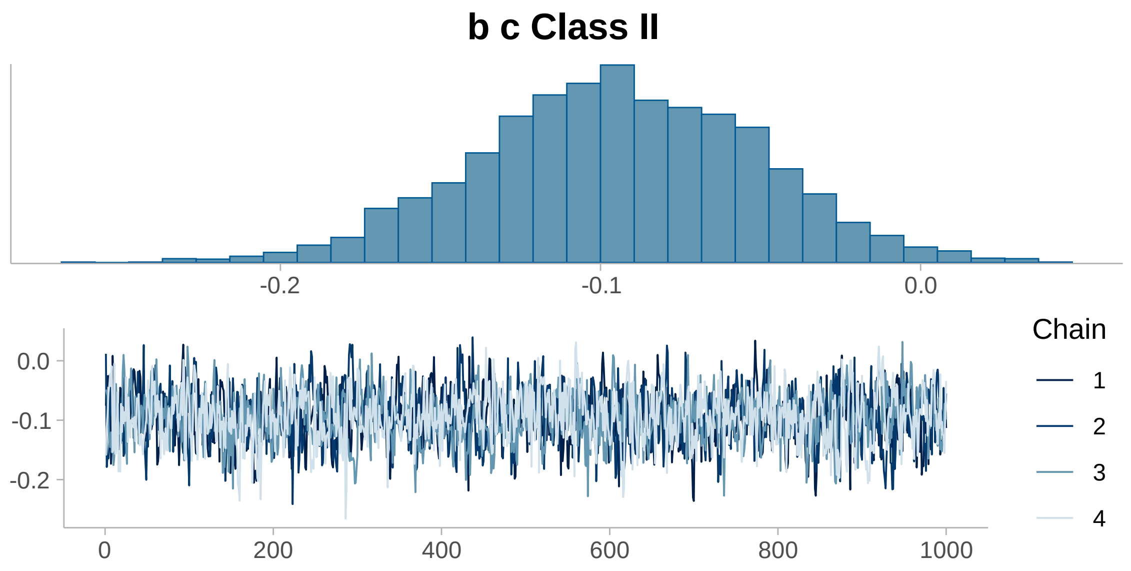
b c Class II
-0.2
-0.1
0.0
0.0
-0.1
-0.2
0
200
400
600
800
1000
Chain
1
2
3
4

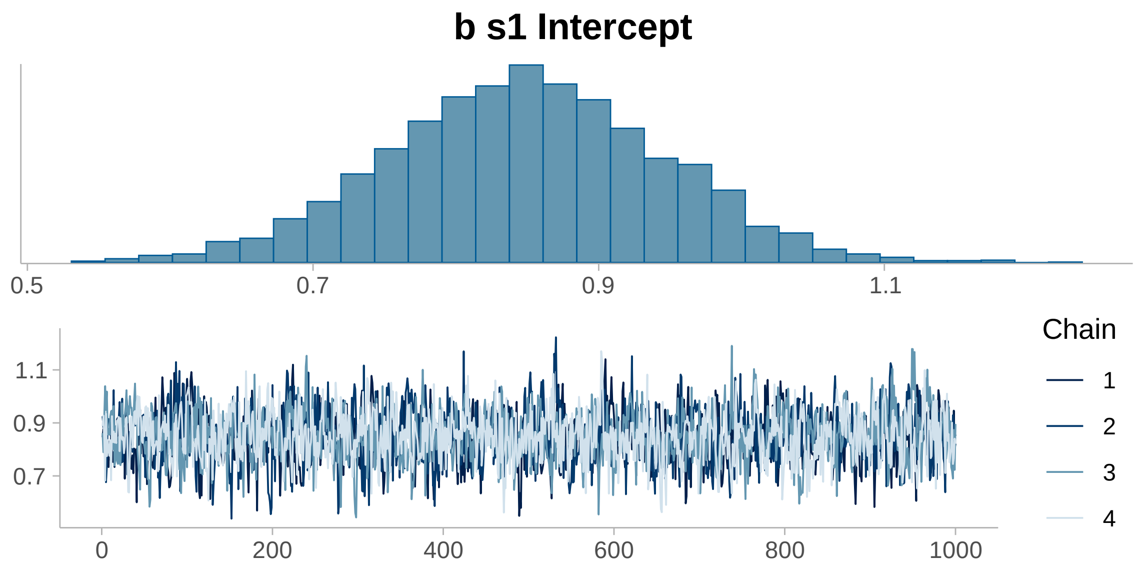

b s1 Intercept
0.5
0.7
0.9
1.1
1.1
0.9
0.7
0
200
400
600
800
1000
Chain
1
2
3
4

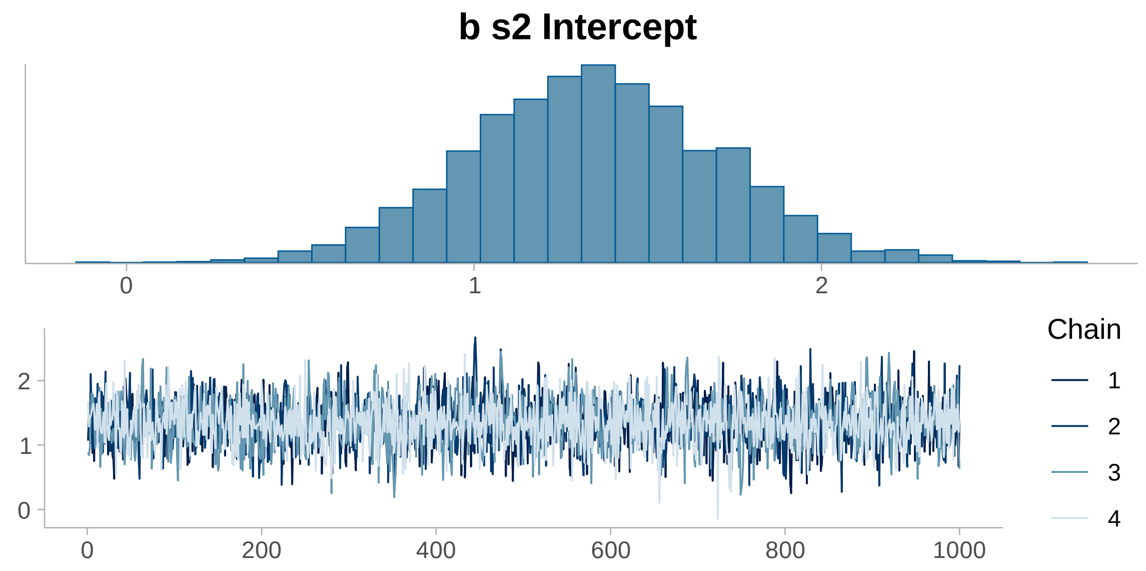
b s2 Intercept
0
1
2
Chain
1
2
3
4
0
200
400
600
800
1000

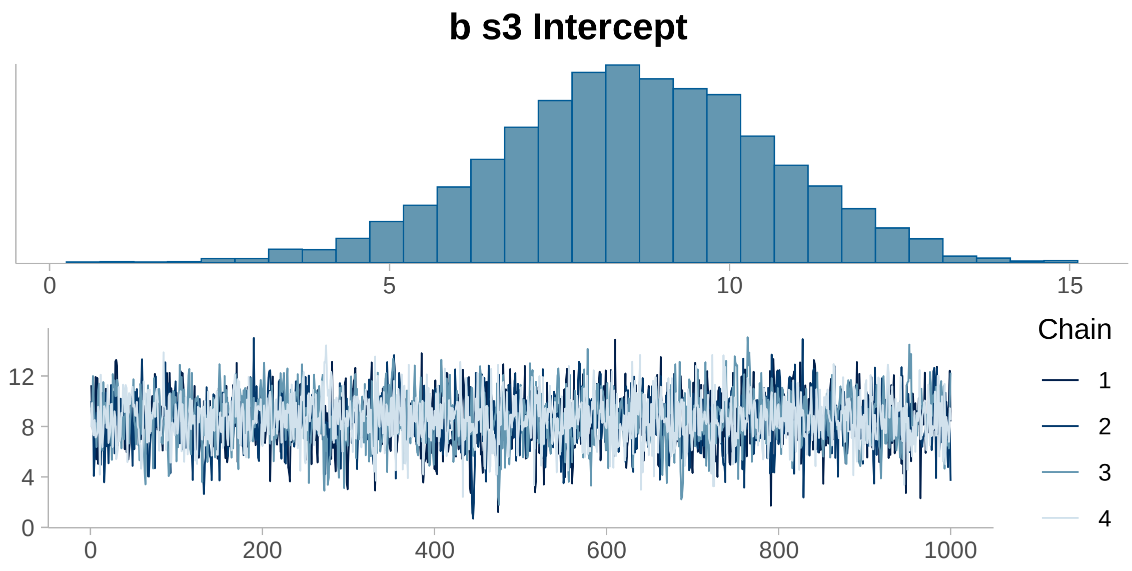
b s3 Intercept
0
5
10
15
Chain
1
2
3
4
12
8
4
0
0
200
400
600
800
1000

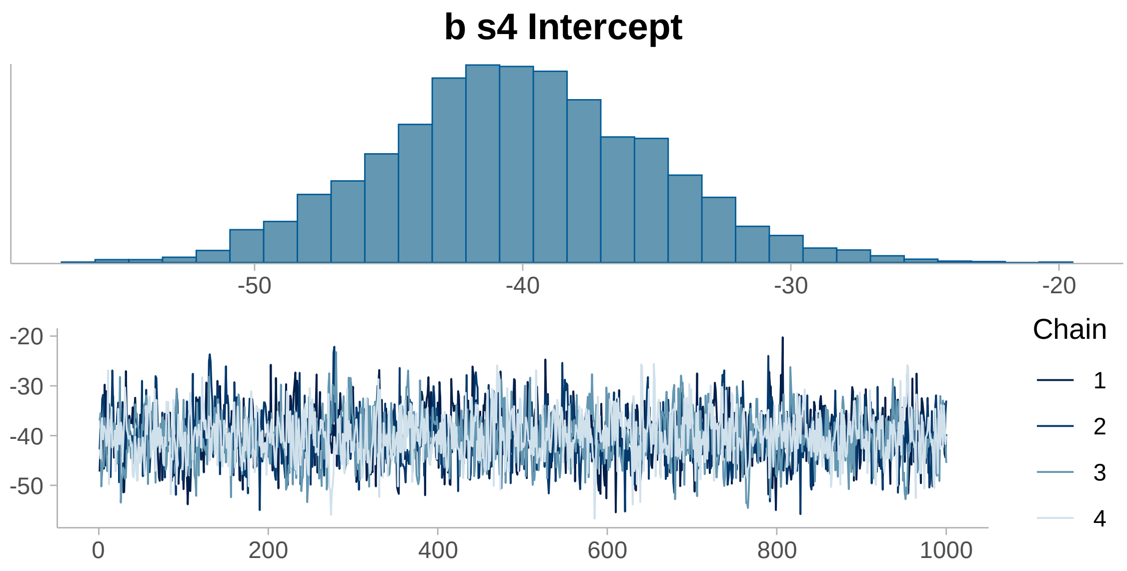
b s4 Intercept
-50
-40
-30
-20
-20
-30
-40
-50
0
200
400
600
800
1000
Chain
1
2
3
4

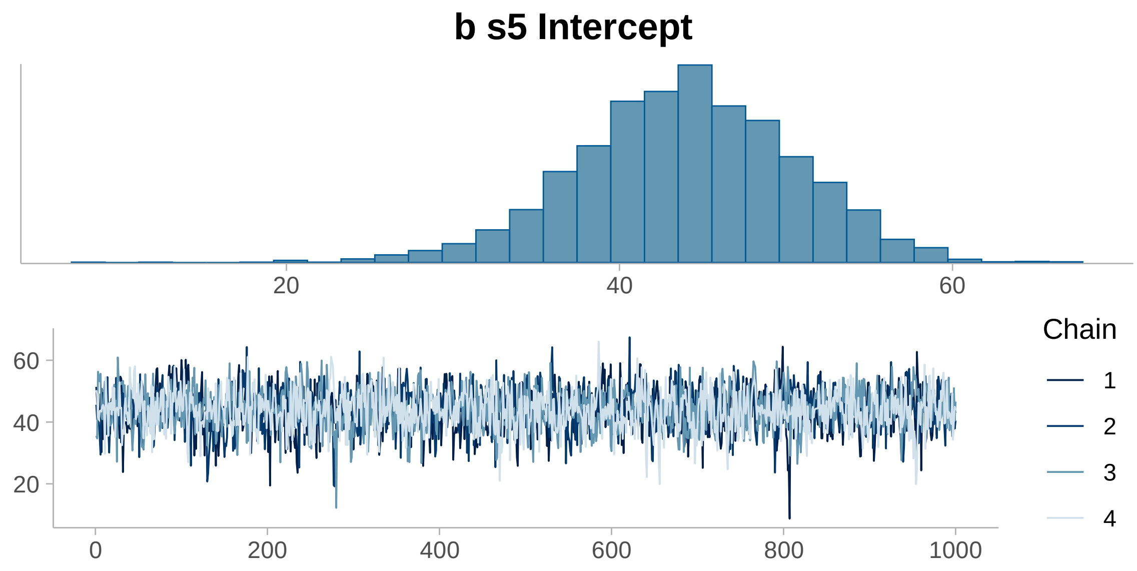
b s5 Intercept
20
40
60
60
40
20
0
200
400
600
800
1000
Chain
1
2
3
4

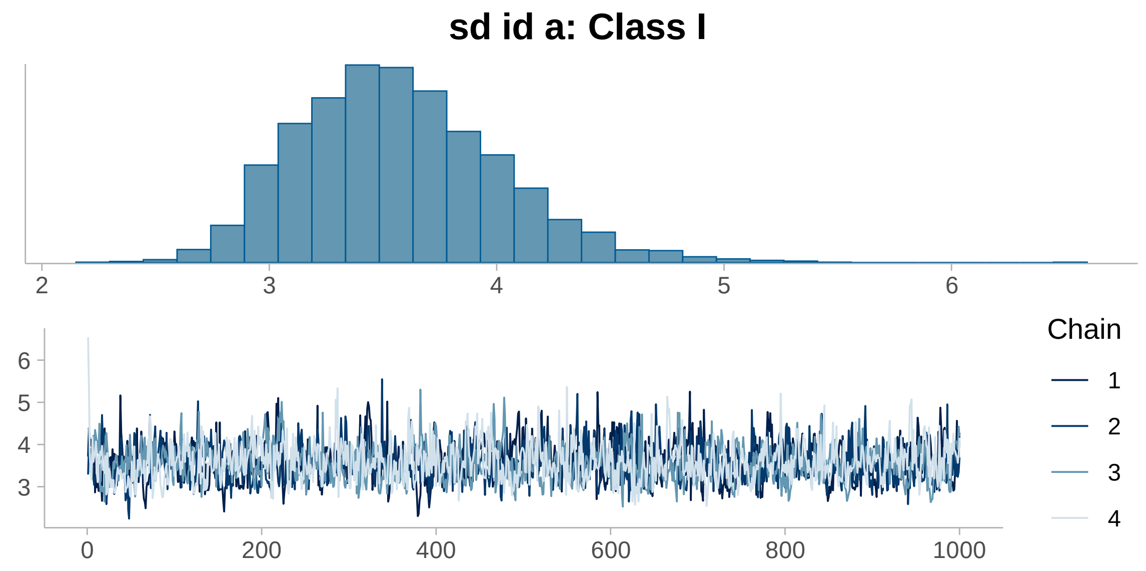
sd id a: Class I
2
3
4
5
6
6
5
4
3
0
200
400
600
800
1000
Chain
1
2
3
4

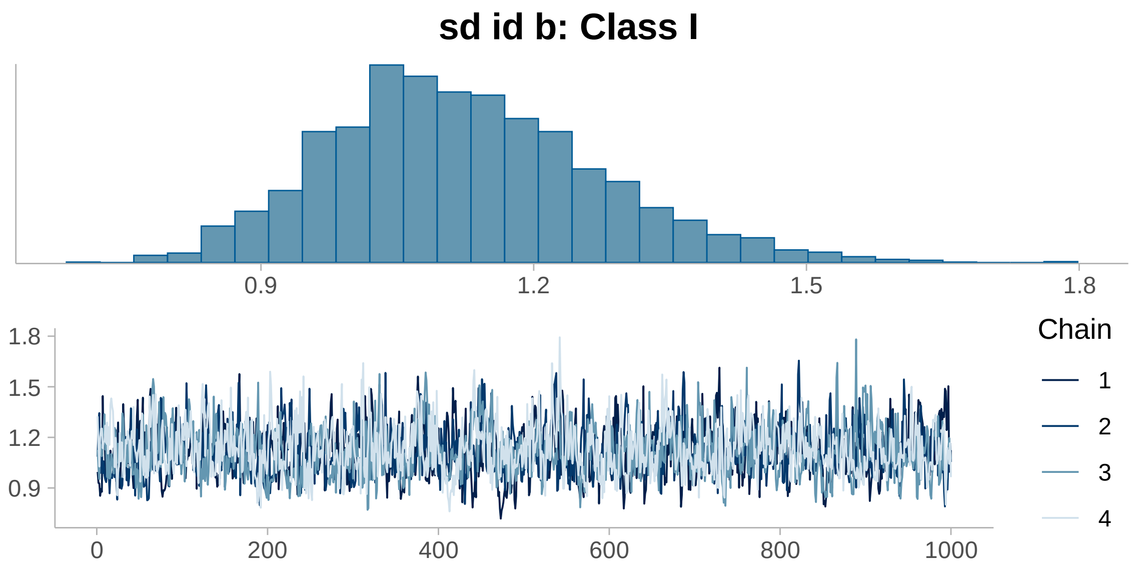
sd id b: Class I
0.9
1.2
1.5
1.8
1.8
1.5
1.2
0.9
0
200
400
600
800
1000
Chain
1
2
3
4

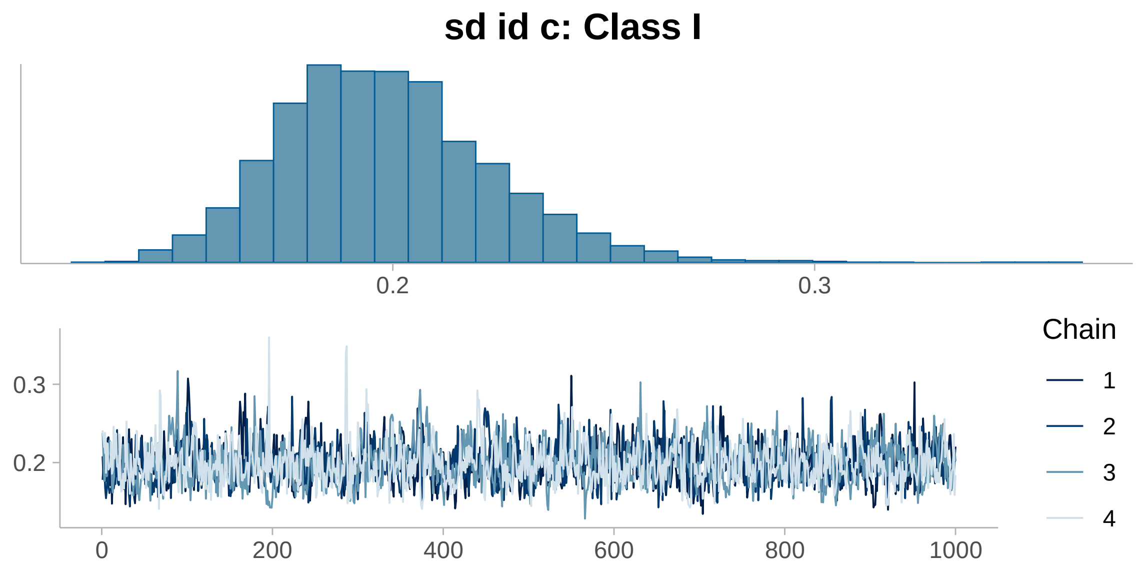
sd id c: Class I
0.2
0.3
Chain
1
2
3
4
0.3
0.2
0
200
400
600
800
1000

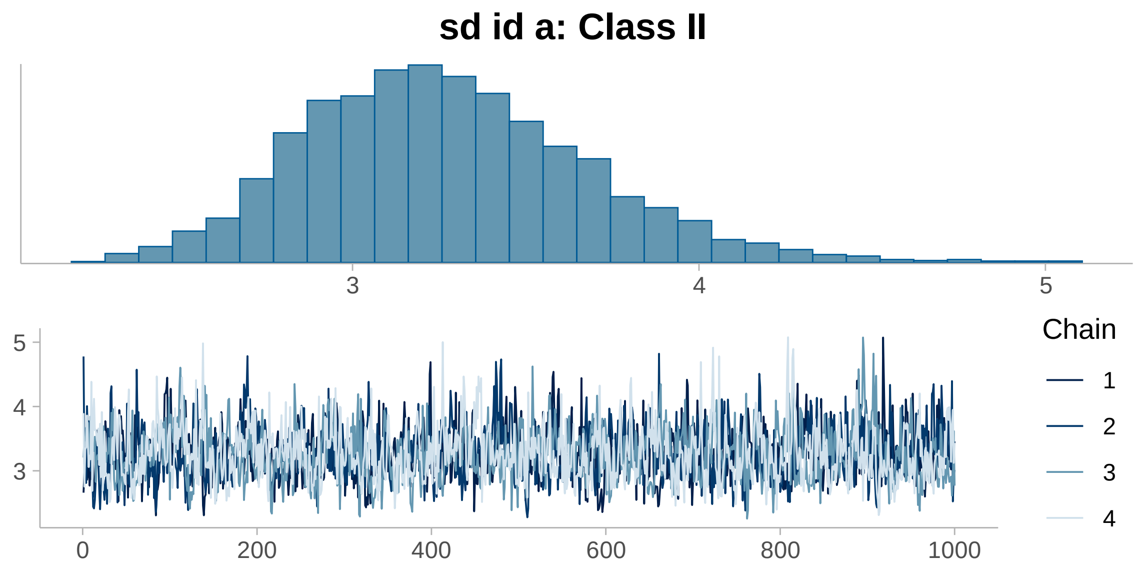
sd id a: Class II
3
4
5
5
4
3
0
200
400
600
800
1000
Chain
1
2
3
4

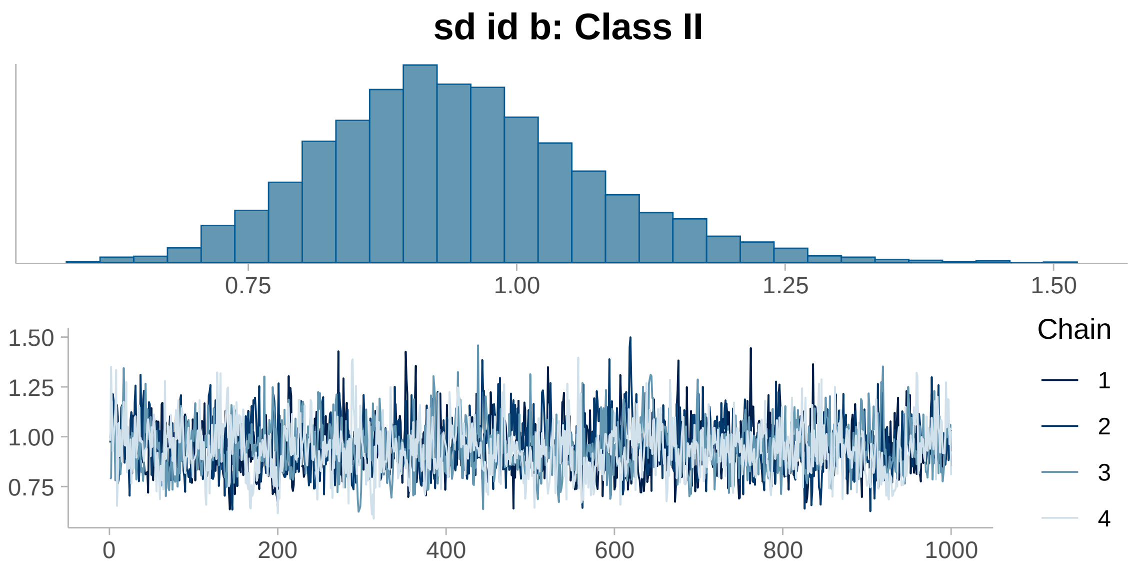
sd id b: Class II
0.75
1.00
1.25
1.50
Chain
1
2
3
4
0
200
400
600
800
1000

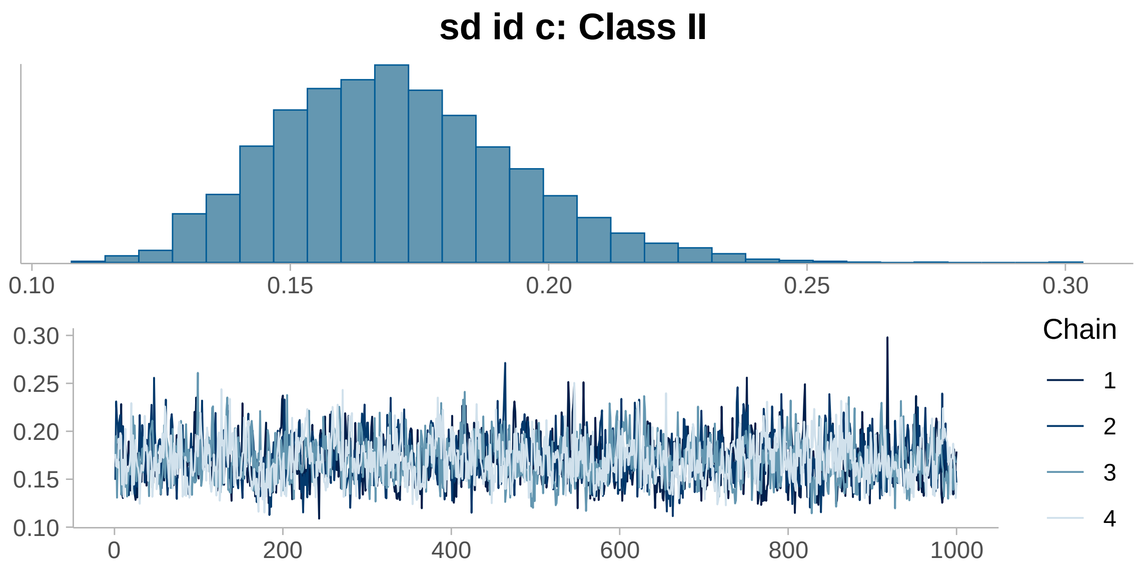

sd id c: Class II
0.10
0.15
0.20
0.25
0.30
Chain
1
2
3
4
0
200
400
600
800
1000

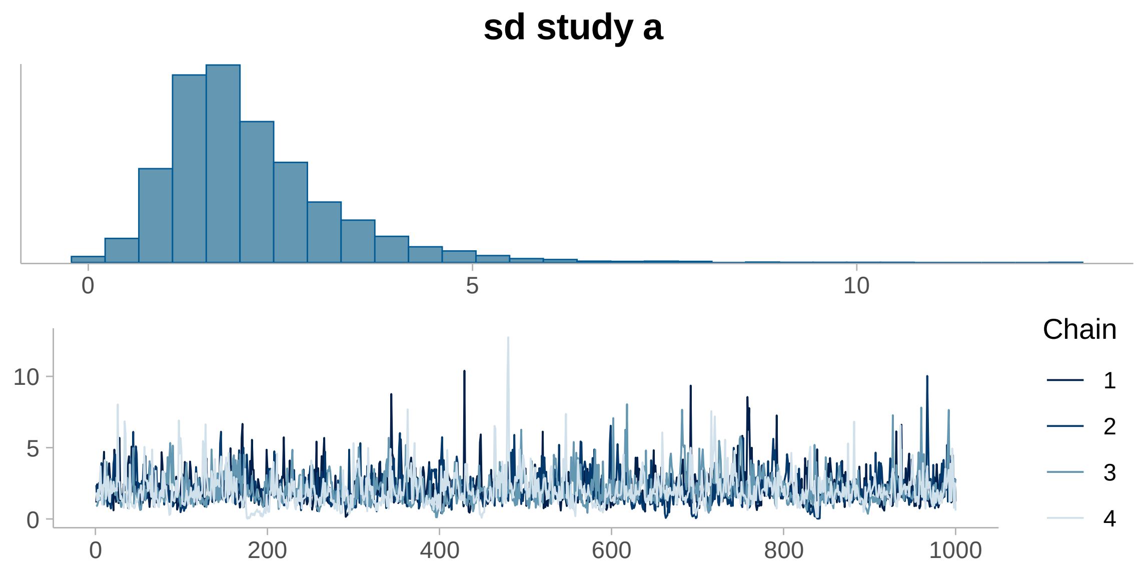

sd study a
0
5
10
Chain
1
2
3
4
10
5
0
0
200
400
600
800
1000

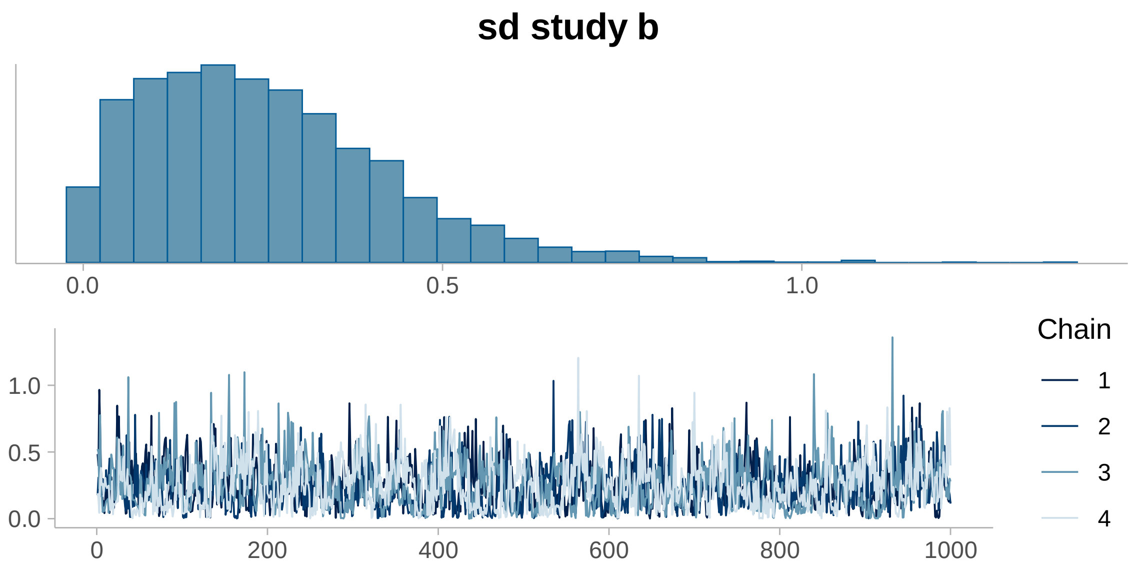
sd study b
0.0
0.5
1.0
Chain
1
2
3
4
1.0
0.5
0.0
0
200
400
600
800
1000

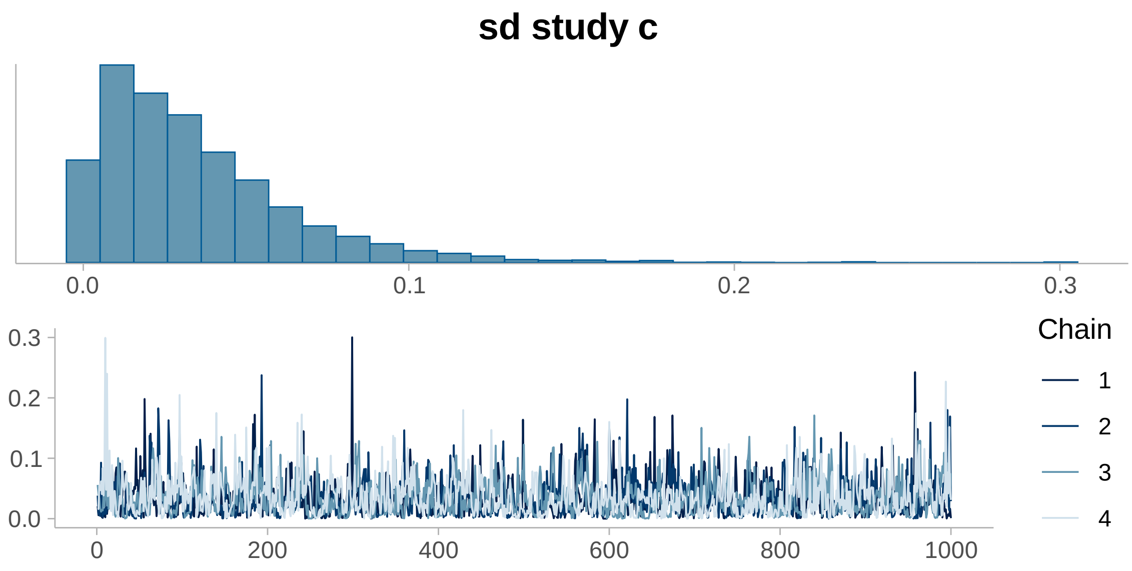

sd study c
0.0
0.1
0.2
0.3
0.3
0.2
0.1
0.0
0
200
400
600
800
1000
Chain
1
2
3
4

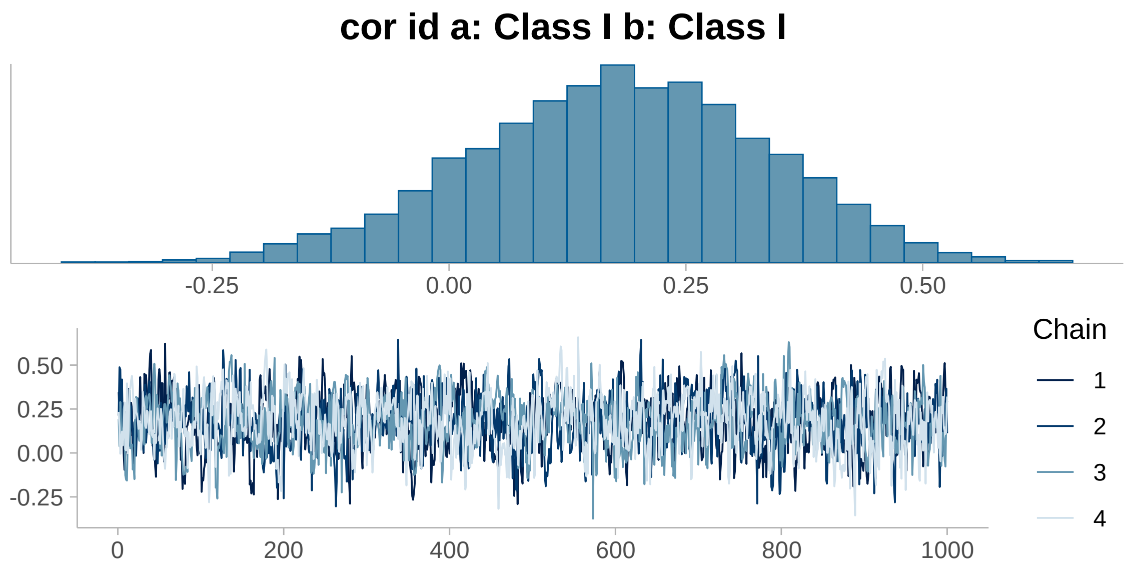
cor id a: Class I b: Class I
-0.25
0.00
0.25
0.50
0.50
0.25
0.00
-0.25
0
200
400
600
800
1000
Chain
1
2
3
4

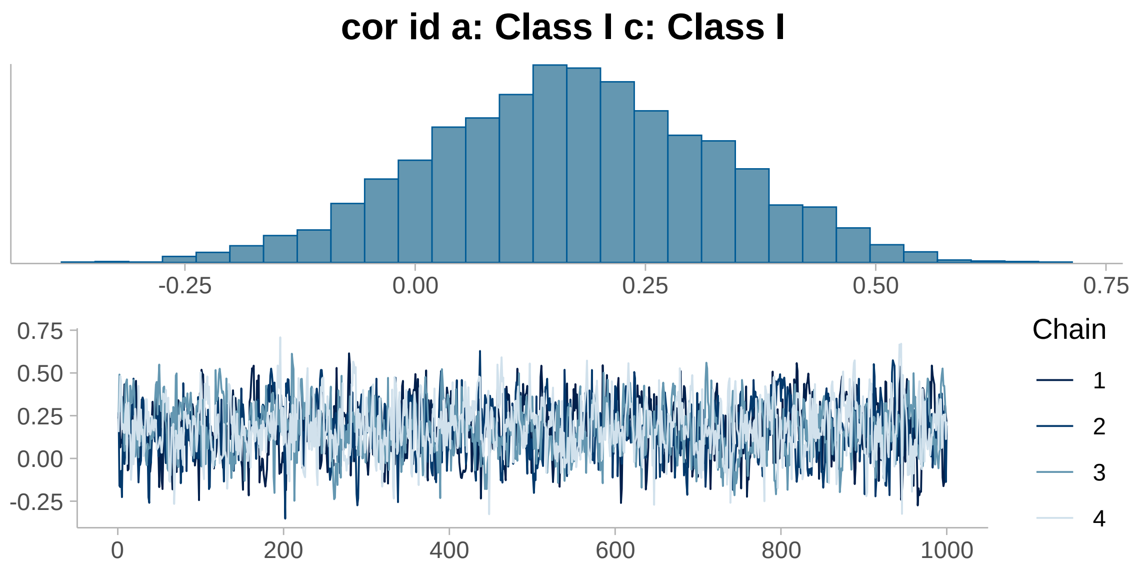

cor id a: Class I c: Class I
-0.25
0.00
0.25
0.50
0.75
0.75
0.50
0.25
0.00
-0.25
0
200
400
600
800
1000
Chain
1
2
3
4

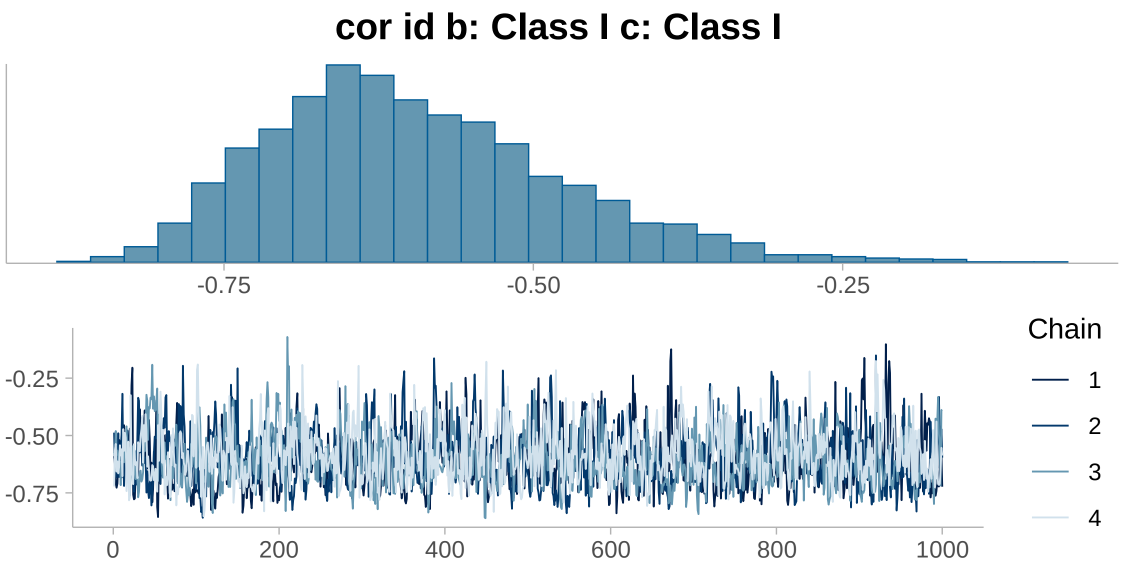
cor id b: Class I c: Class I
-0.75
-0.50
-0.25
-0.25
-0.50
-0.75
0
200
400
600
800
1000
Chain
1
2
3
4

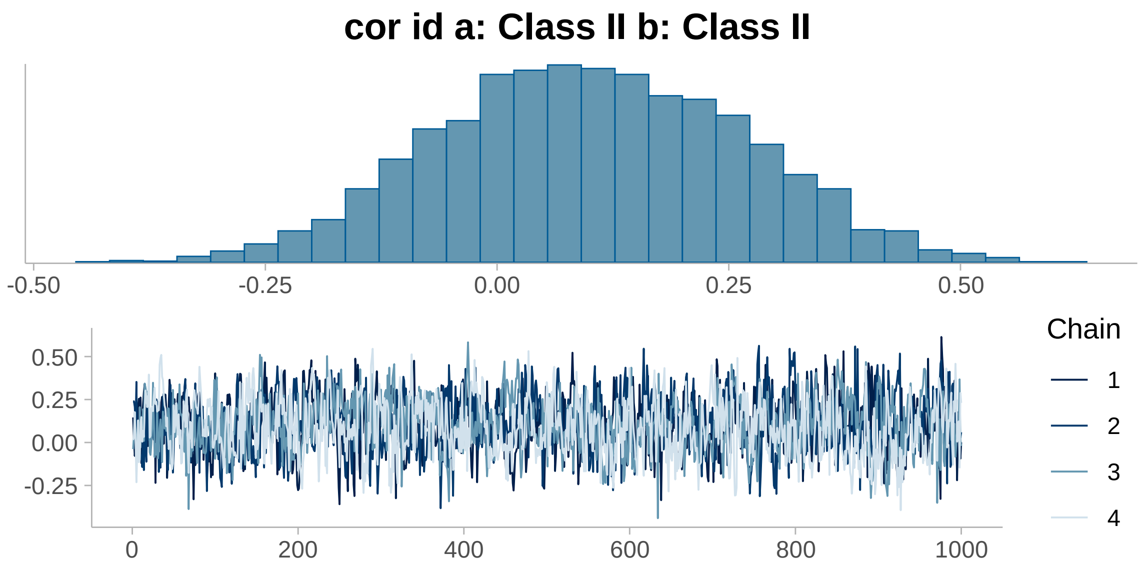
cor id a: Class II b: Class II
-0.50
-0.25
0.00
0.25
0.50
0.50
0.25
0.00
-0.25
0
200
400
600
800
1000
Chain
1
2
3
4

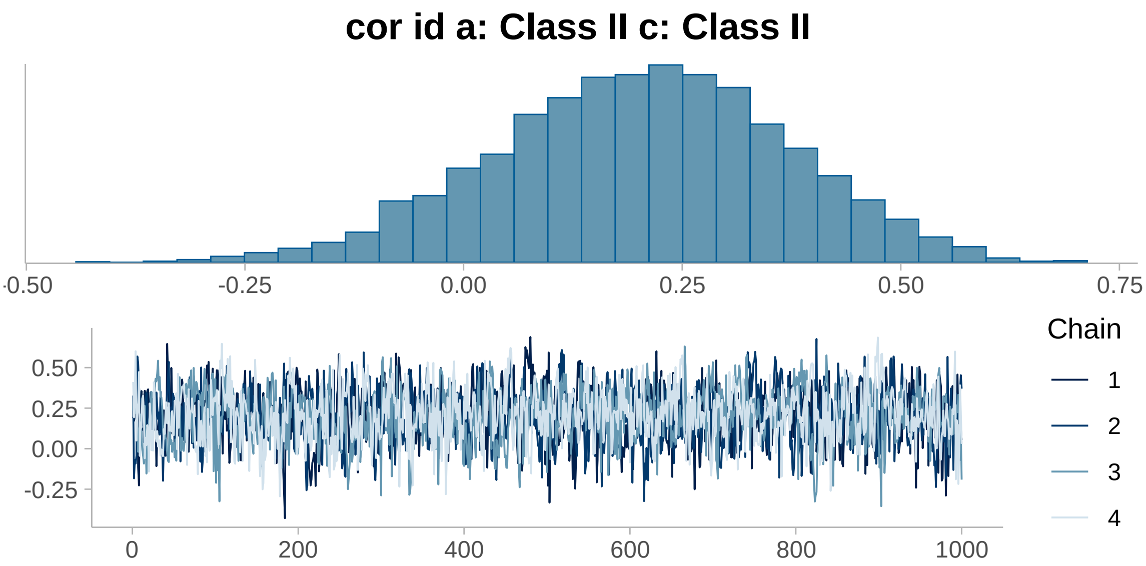
cor id a: Class II c: Class II
-0.50
-0.25
0.00
0.25
0.50
0.75
0.50
0.25
0.00
-0.25
0
200
400
600
800
1000
Chain
1
2
3
4

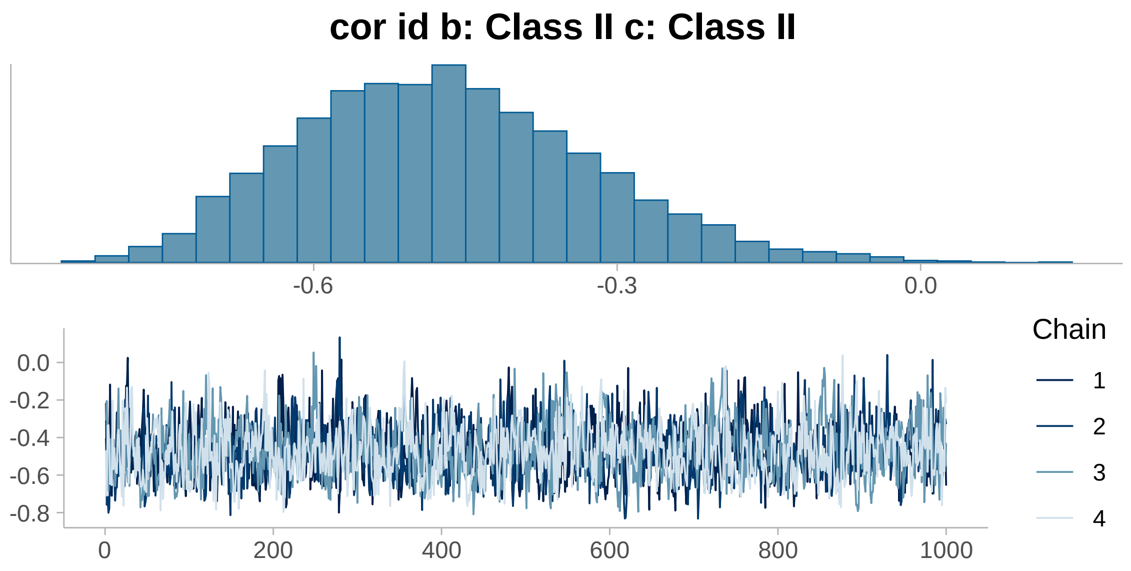
cor id b: Class II c: Class II
-0.6
-0.3
0.0
0.0
-0.2
-0.4
-0.6
-0.8
0
200
400
600
800
1000
Chain
1
2
3
4

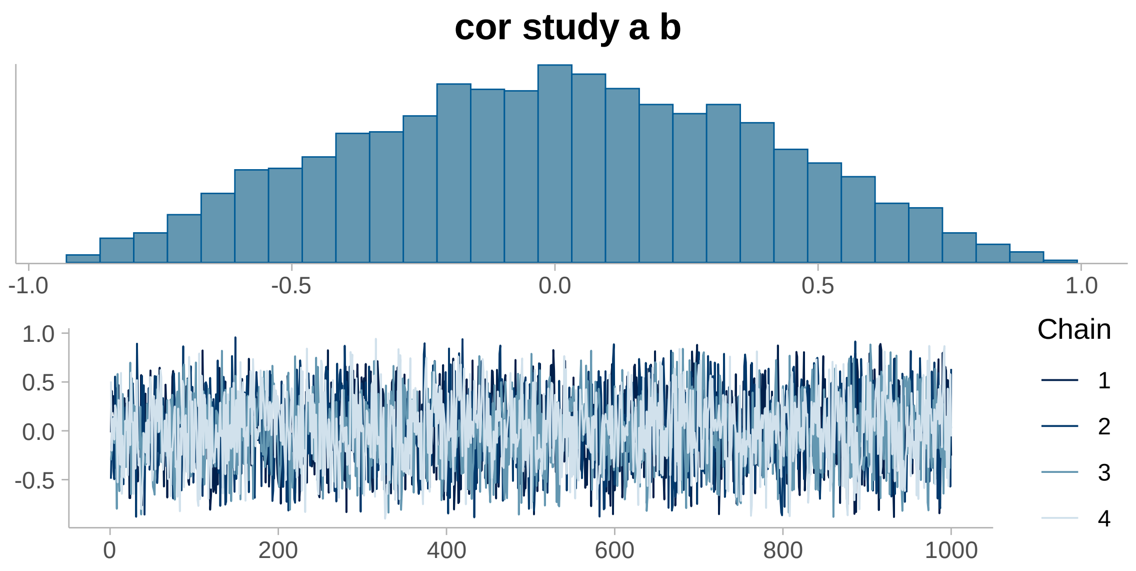
cor study a b
-1.0
-0.5
0.0
0.5
1.0
1.0
0.5
0.0
-0.5
0
200
400
600
800
1000
Chain
1
2
3
4

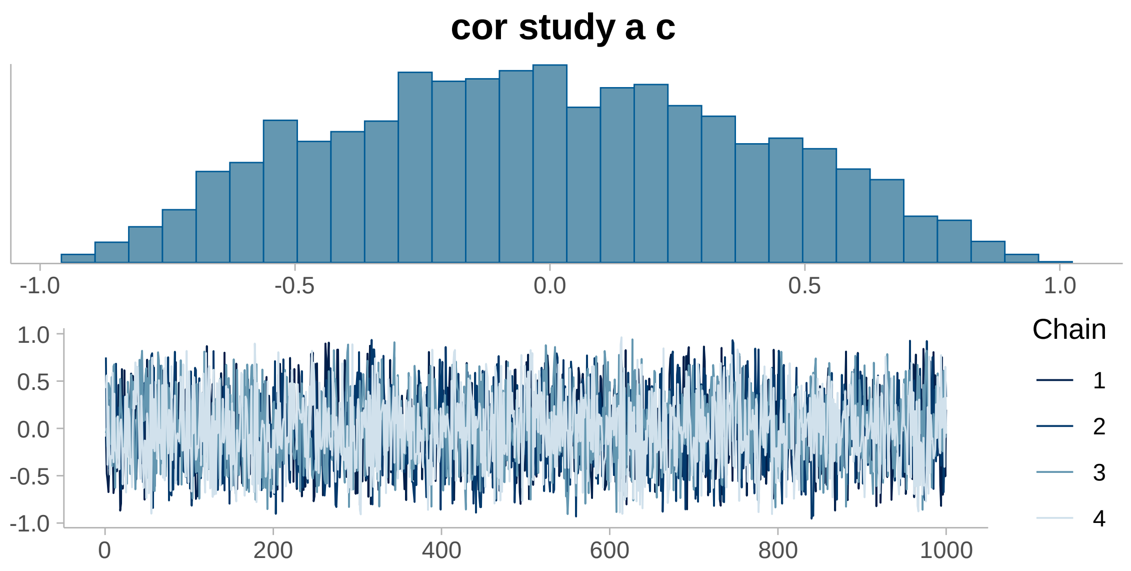
cor study a c
-1.0
-0.5
0.0
0.5
1.0
Chain
1
2
3
4
0
200
400
600
800
1000

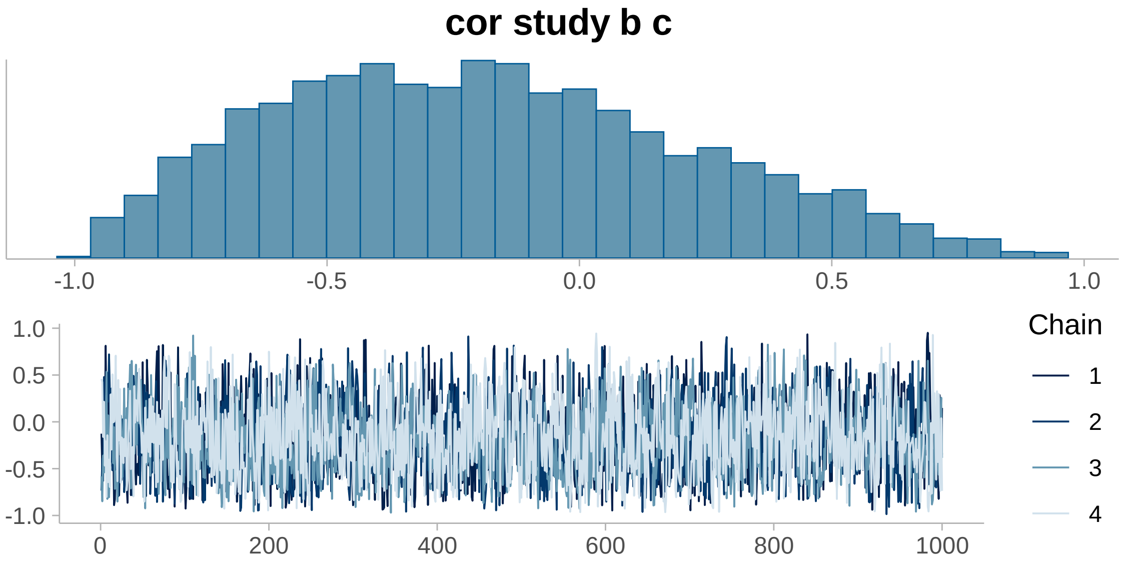

cor study b c
-1.0
-0.5
0.0
0.5
1.0
1.0
0.5
0.0
-0.5
-1.0
0
200
400
600
800
1000
Chain
1
2
3
4

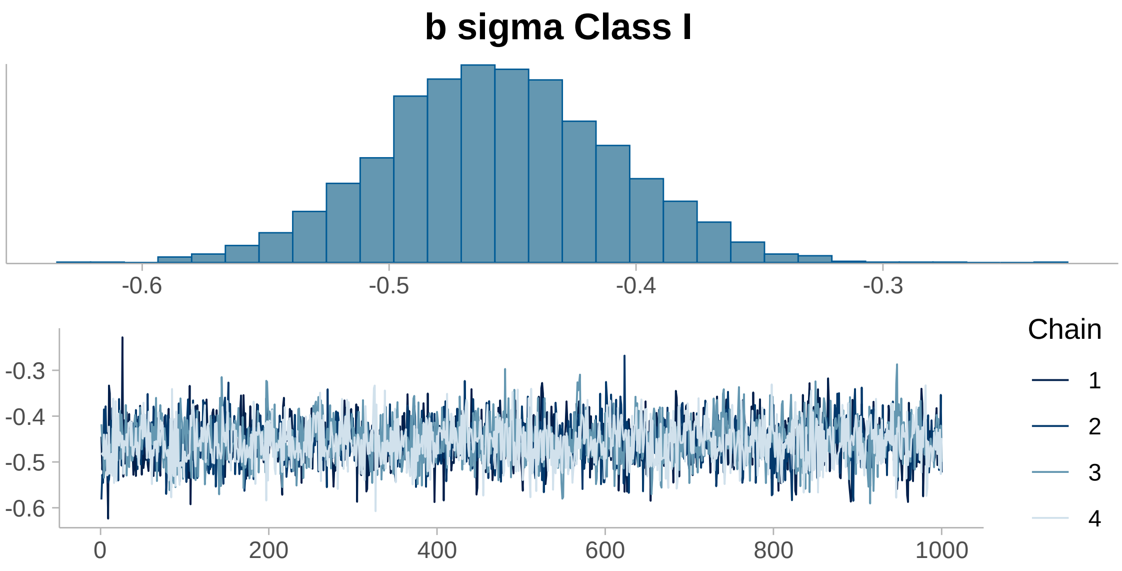
b sigma Class I
-0.6
-0.5
-0.4
-0.3
-0.3
-0.4
-0.5
-0.6
0
200
400
600
800
1000
Chain
1
2
3
4

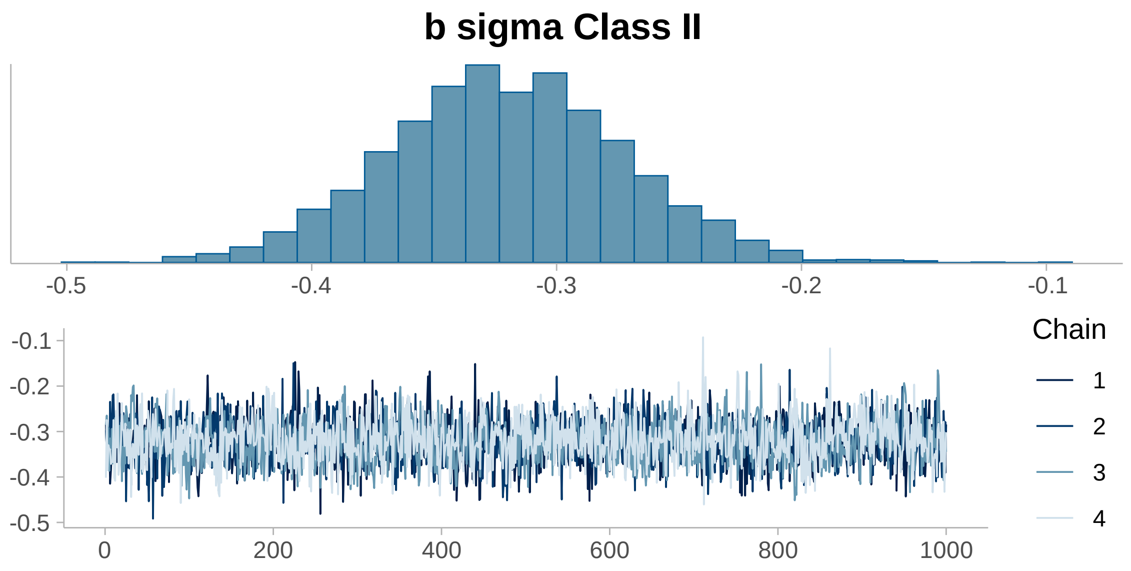
b sigma Class II
-0.5
-0.4
-0.3
-0.2
-0.1
Chain
1
2
3
4
0
200
400
600
800
1000

MCMC diagnostic plots for females

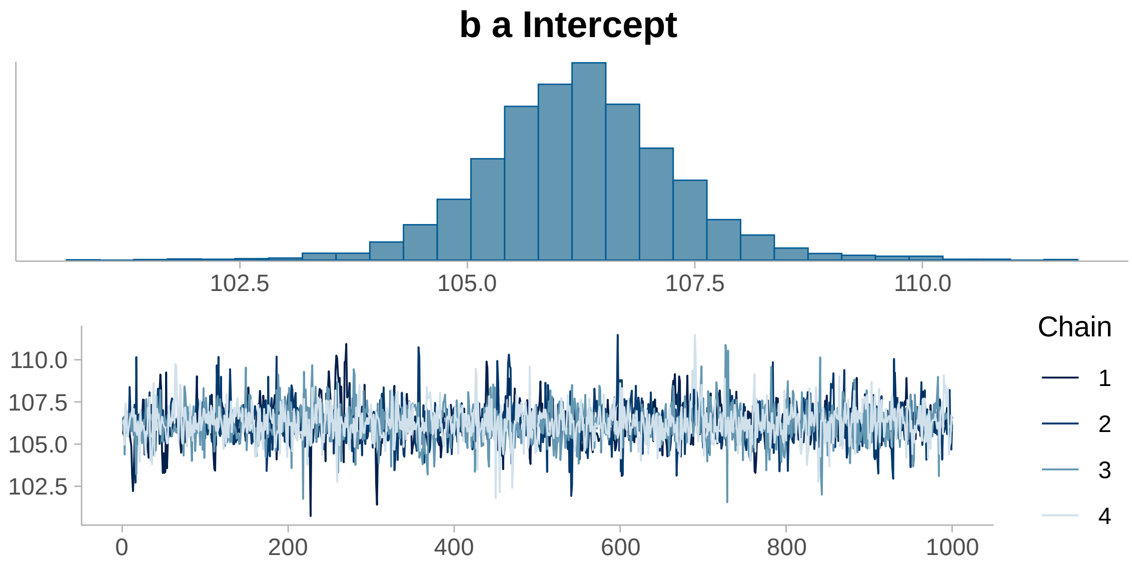

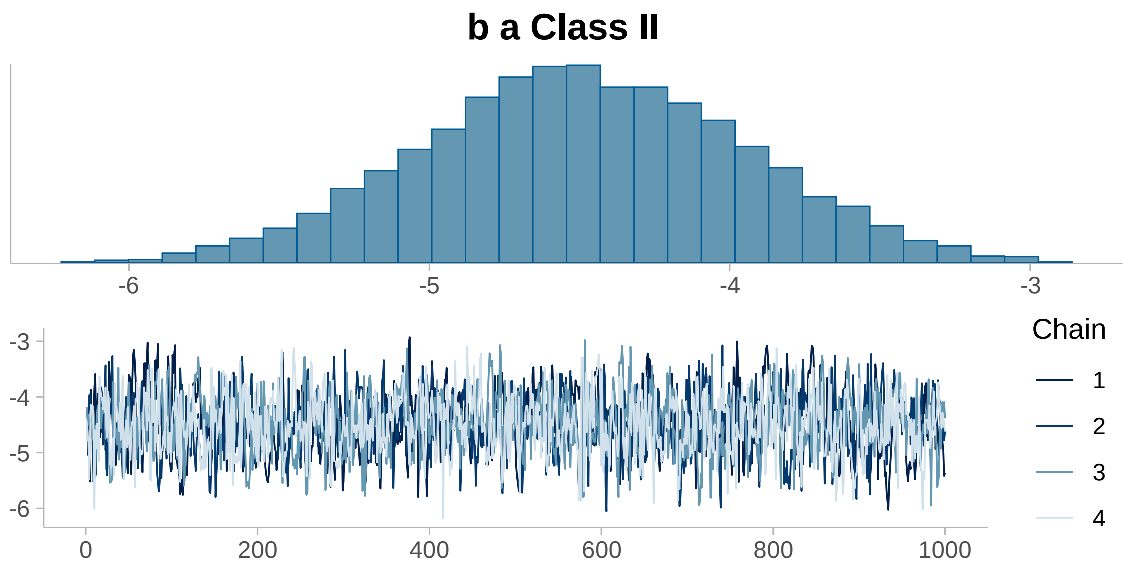
b a Class II
-6
-5
-4
-3
Chain
1
2
3
4
0
200
400
600
800
1000

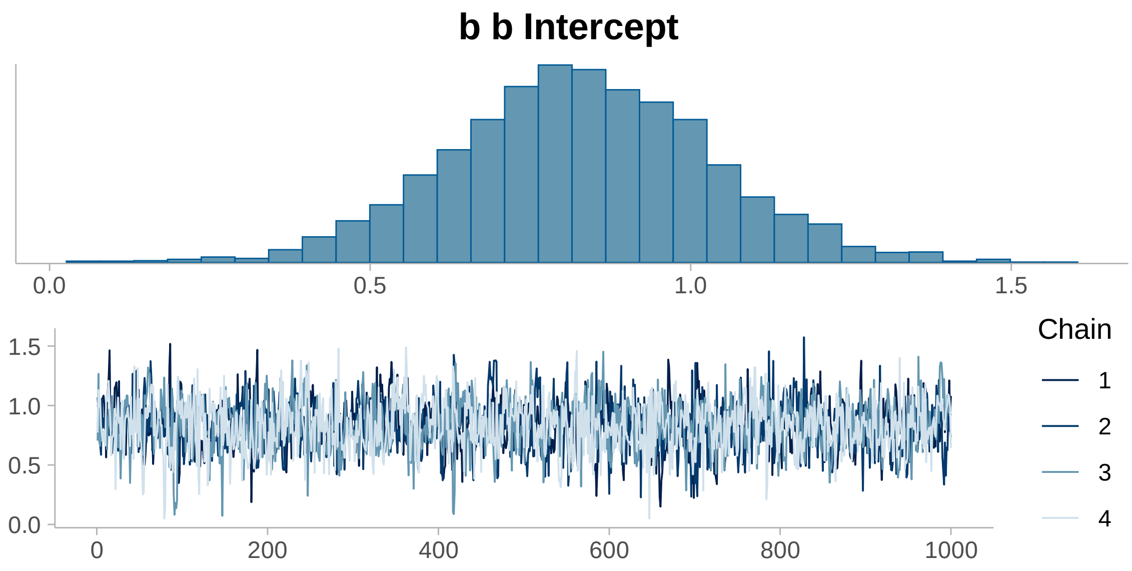

b b Intercept
0.0
0.5
1.0
1.5
1.5
1.0
0.5
0.0
0
200
400
600
800
1000
Chain
1
2
3
4

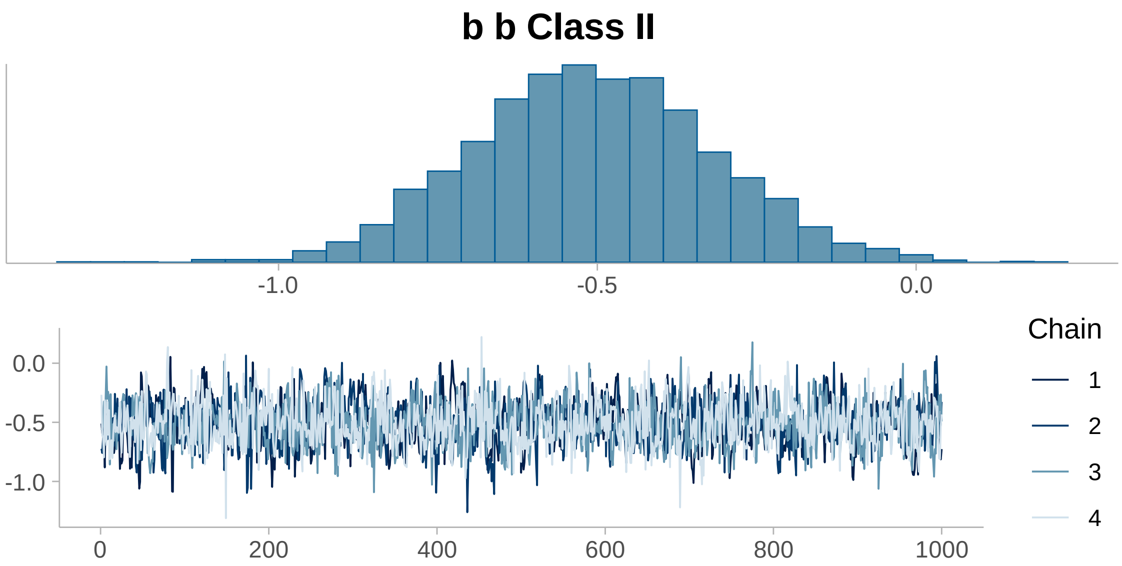
b b Class II
-1.0
-0.5
0.0
0.0
-0.5
-1.0
0
200
400
600
800
1000
Chain
1
2
3
4

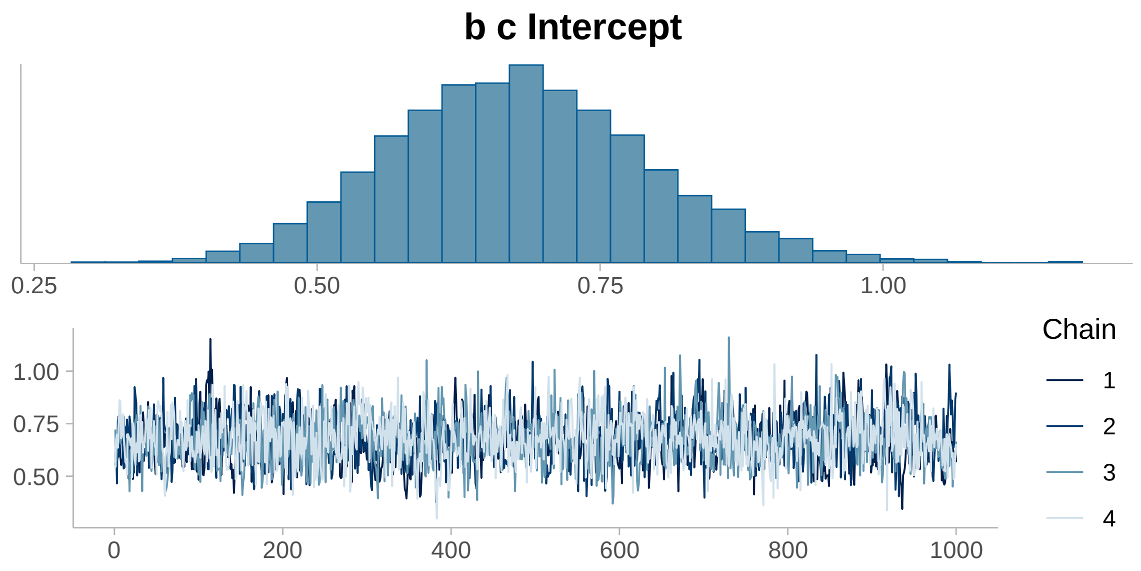

b c Intercept
0.25
0.50
0.75
1.00
1.00
0.75
0.50
0
200
400
600
800
1000
Chain
1
2
3
4

b c Class II

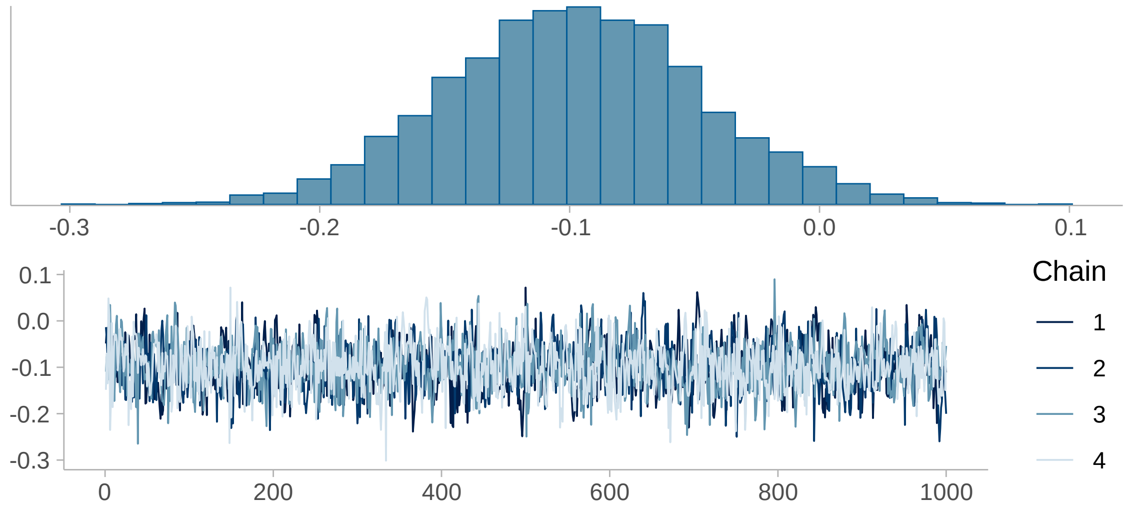

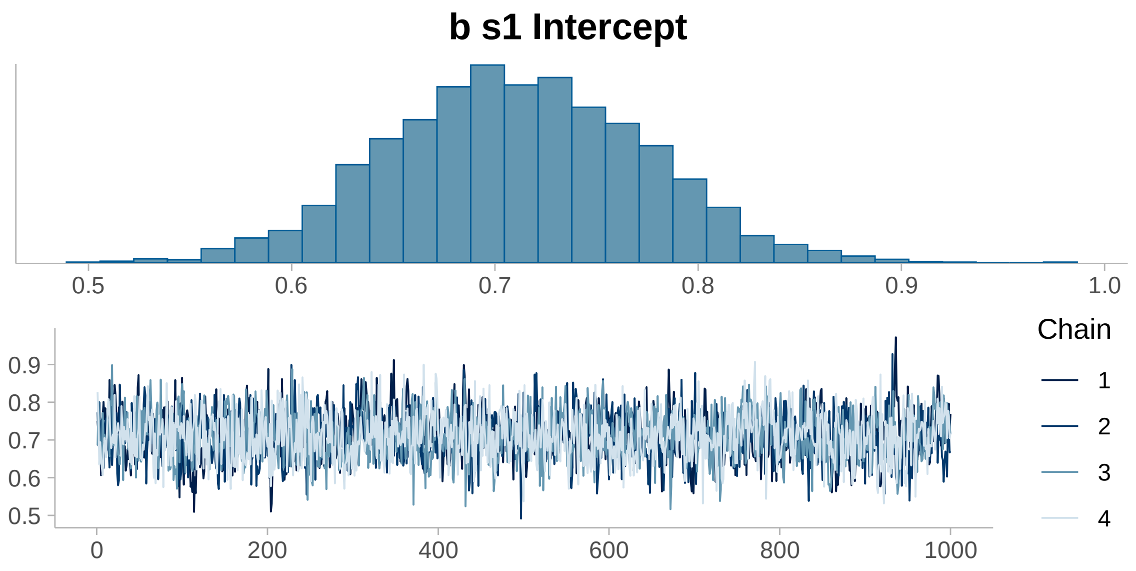

b s1 Intercept
0.5
0.6
0.7
0.8
0.9
1.0
0.9
0.8
0.7
0.6
0.5
0
200
400
600
800
1000
Chain
1
2
3
4

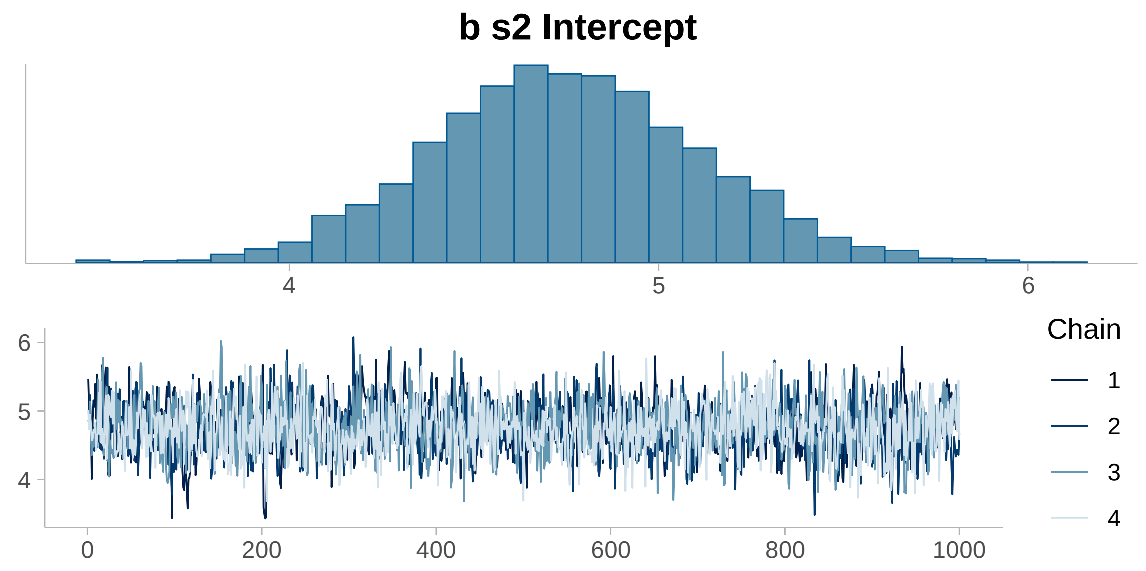
b s2 Intercept
4
5
6
6
5
4
0
200
400
600
800
1000
Chain
1
2
3
4

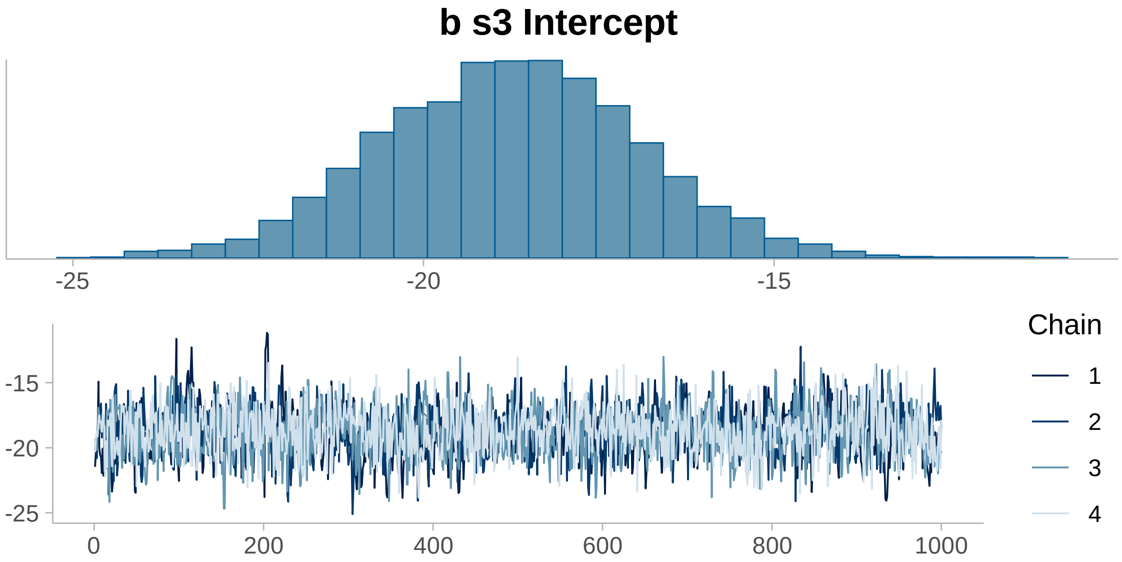
b s3 Intercept
-25
-20
-15
Chain
1
2
3
4
-15
-20
-25
0
200
400
600
800
1000

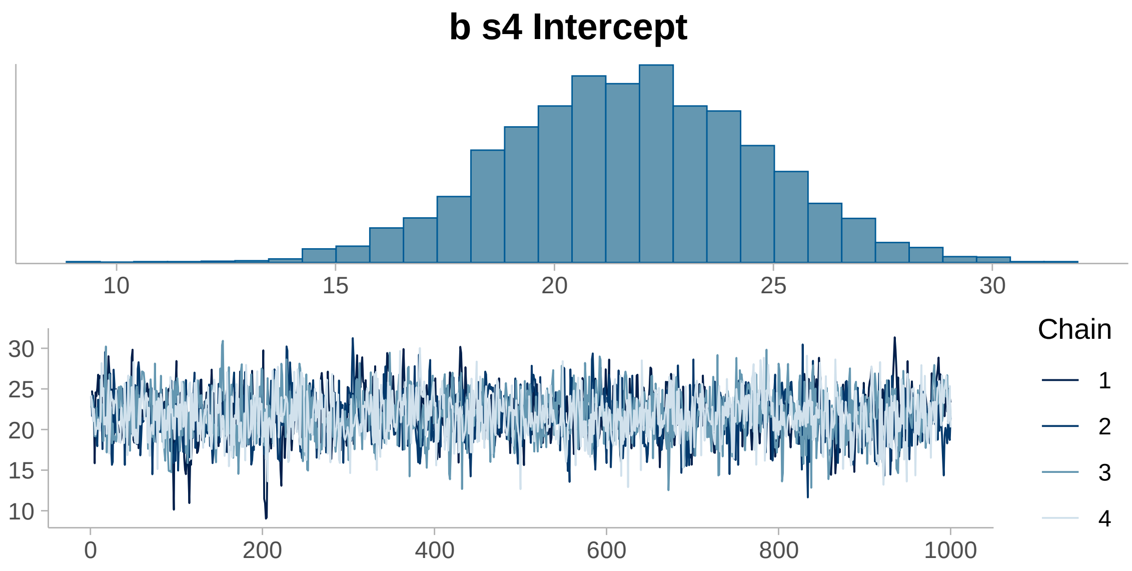
b s4 Intercept
10
15
20
25
30
30
25
20
15
10
0
200
400
600
800
1000
Chain
1
2
3
4

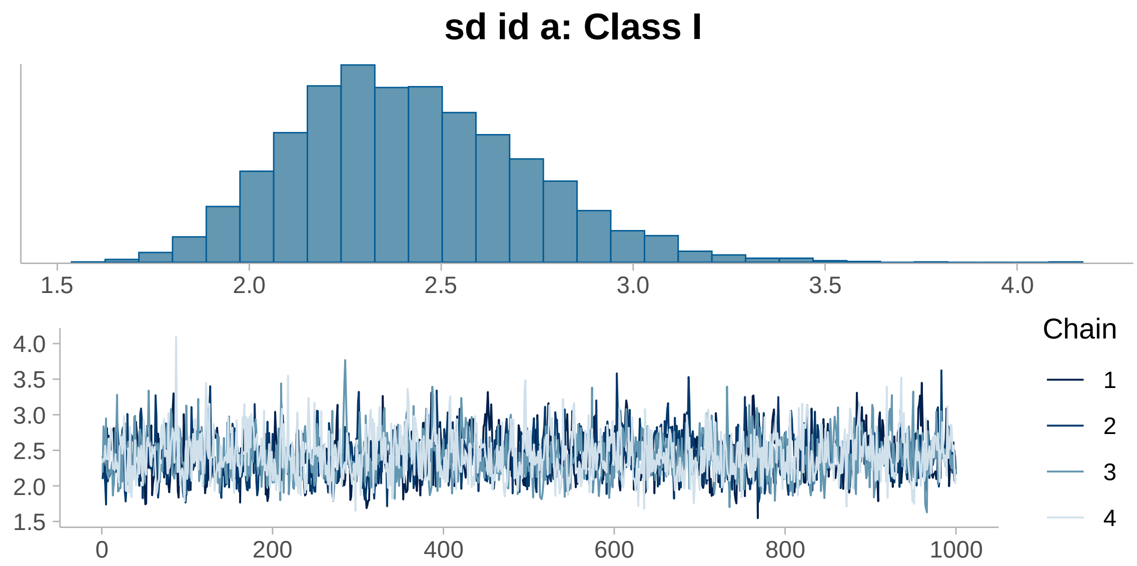

sd id a: Class I
1.5
2.0
2.5
3.0
3.5
4.0
4.0
3.5
3.0
2.5
2.0
1.5
0
200
400
600
800
1000
Chain
1
2
3
4

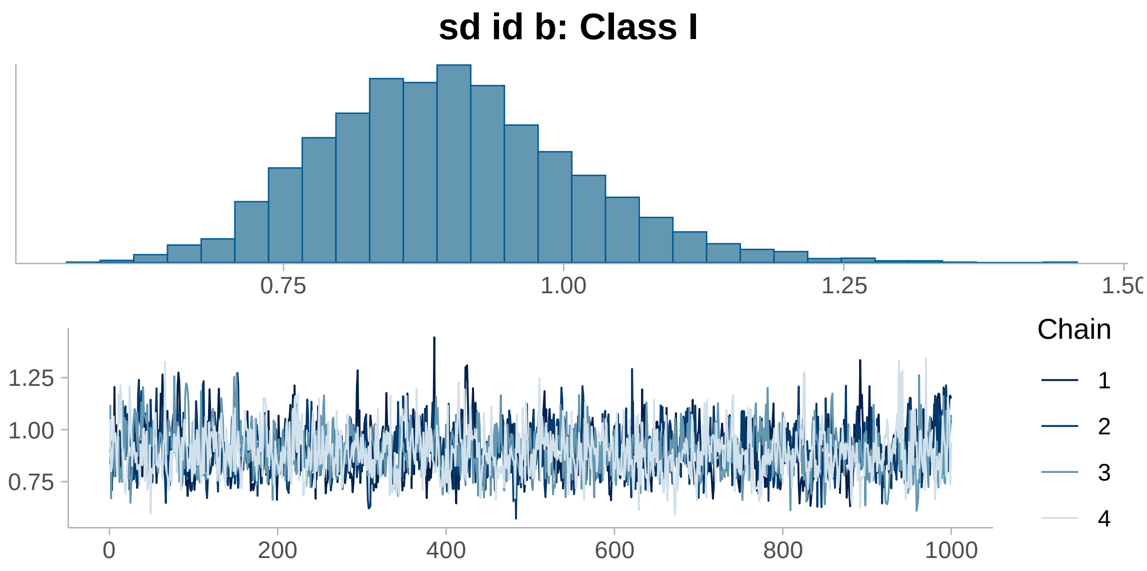
sd id b: Class I
0.75
1.00
1.25
1.50
1.25
1.00
0.75
0
200
400
600
800
1000
Chain
1
2
3
4

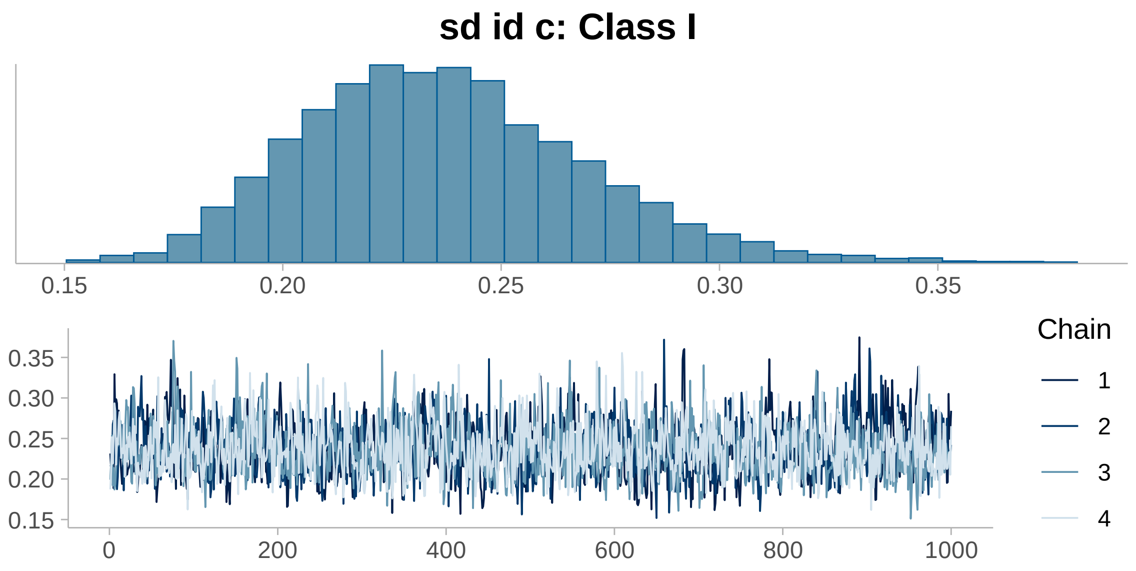
sd id c: Class I
0.15
0.20
0.25
0.30
0.35
Chain
1
2
3
4
0
200
400
600
800
1000

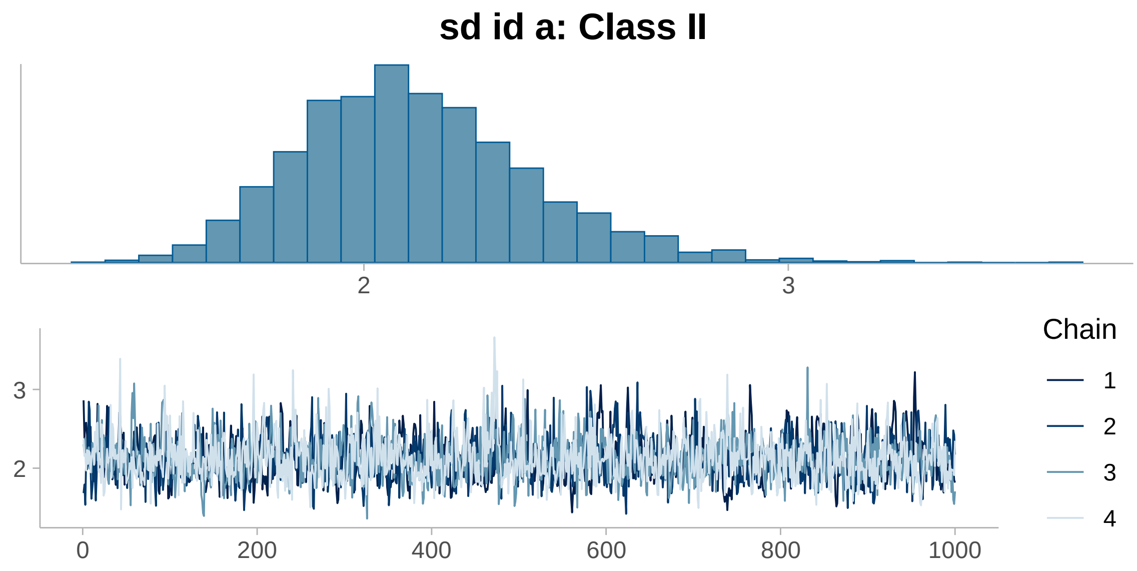

sd id a: Class II
2
3
Chain
1
2
3
4
3
2
0
200
400
600
800
1000

sd id b: Class II

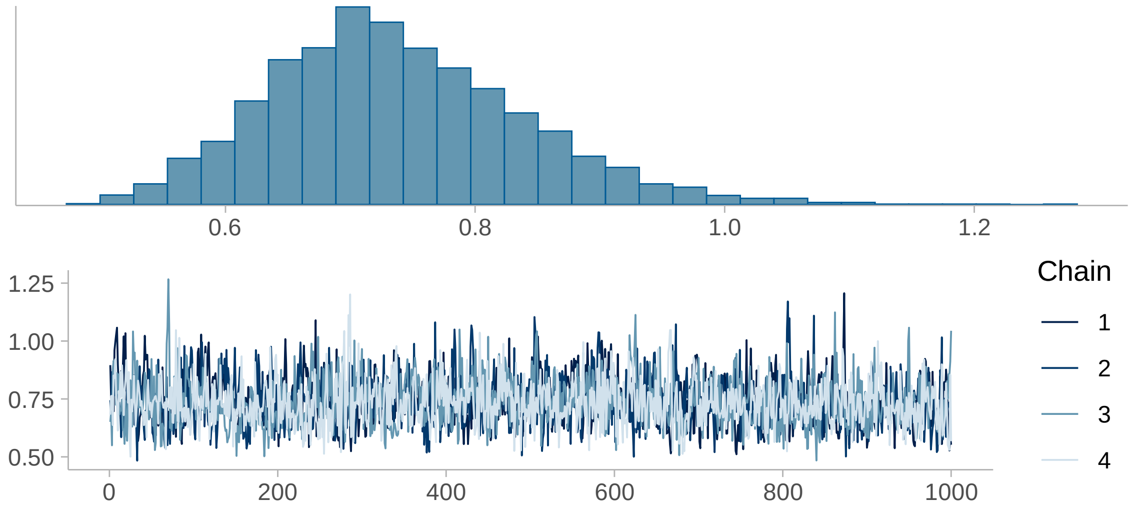

sd id c: Class II

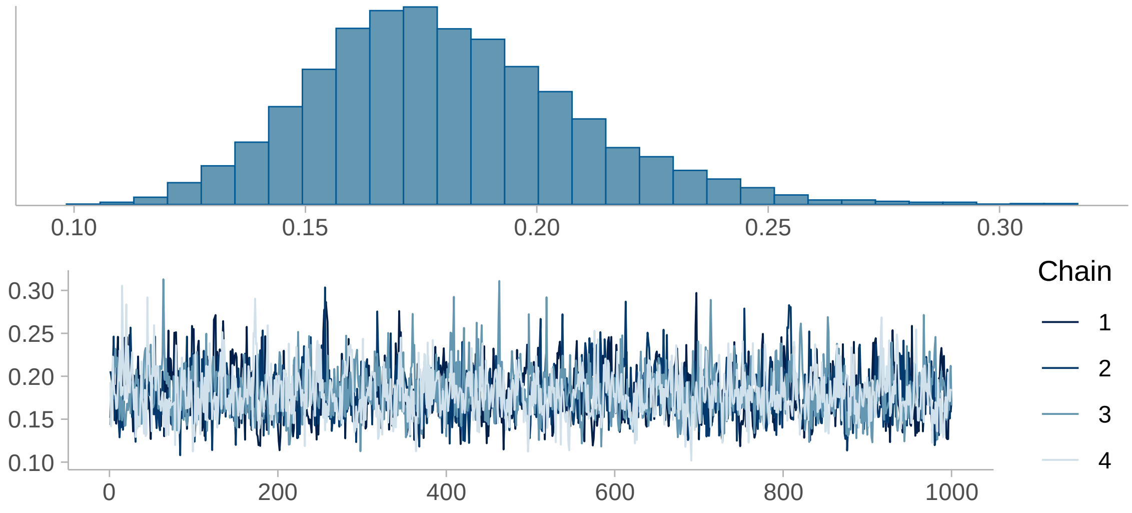

sd study a

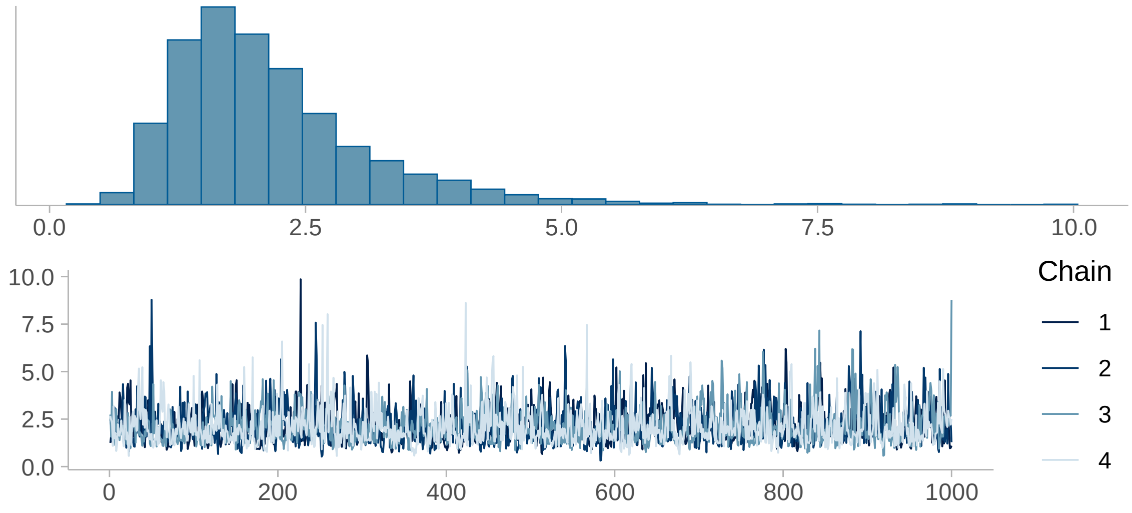

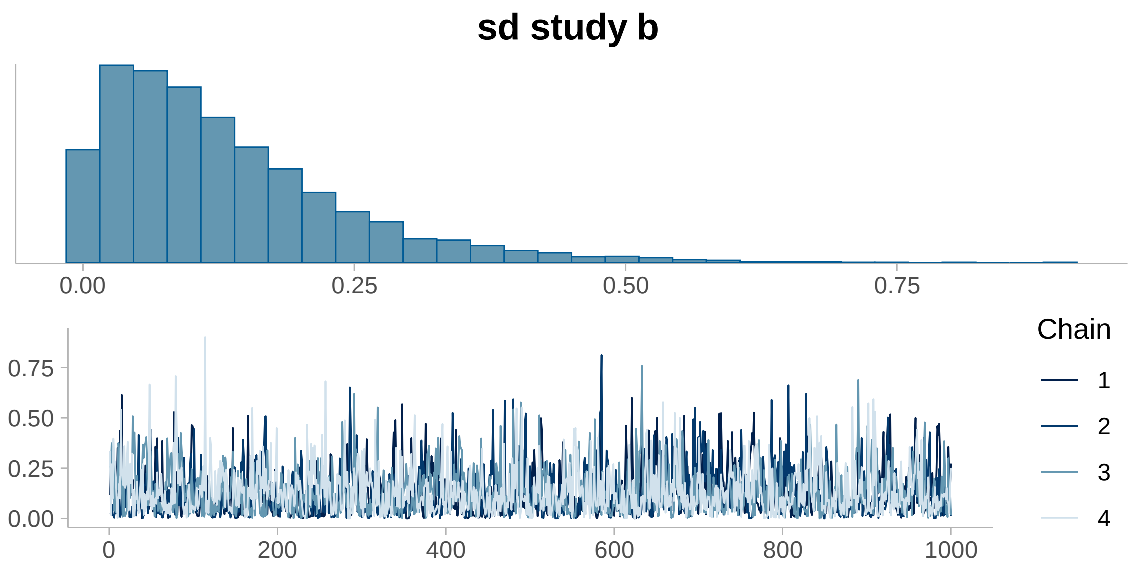

sd study b
0.00
0.25
0.50
0.75
0.75
0.50
0.25
0.00
0
200
400
600
800
1000
Chain
1
2
3
4

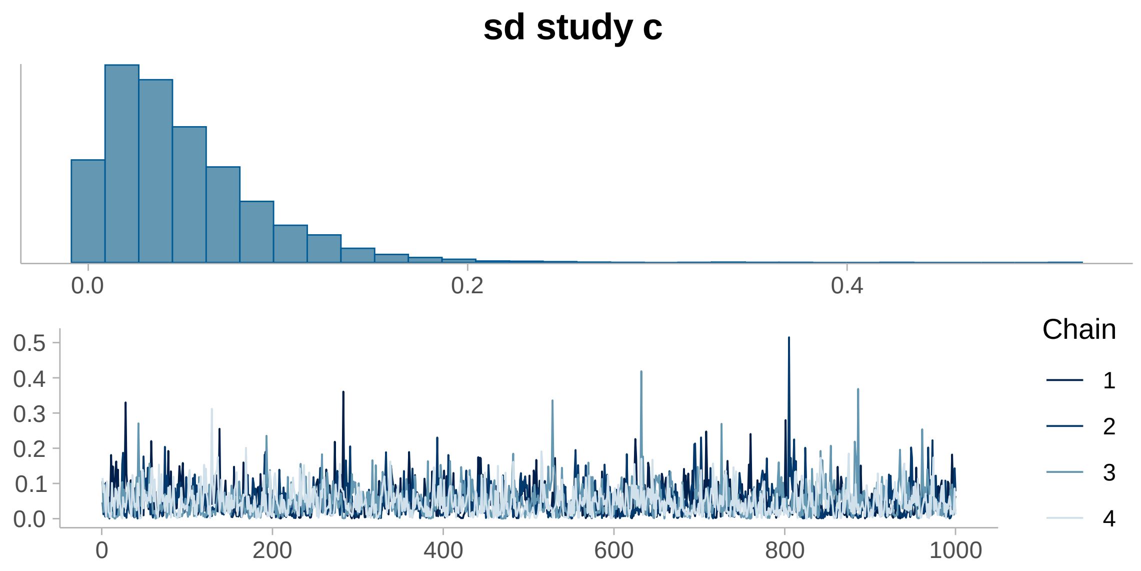

sd study c
0.0
0.2
0.4
0.5
0.4
0.3
0.2
0.1
0.0
0
200
400
600
800
1000
Chain
1
2
3
4

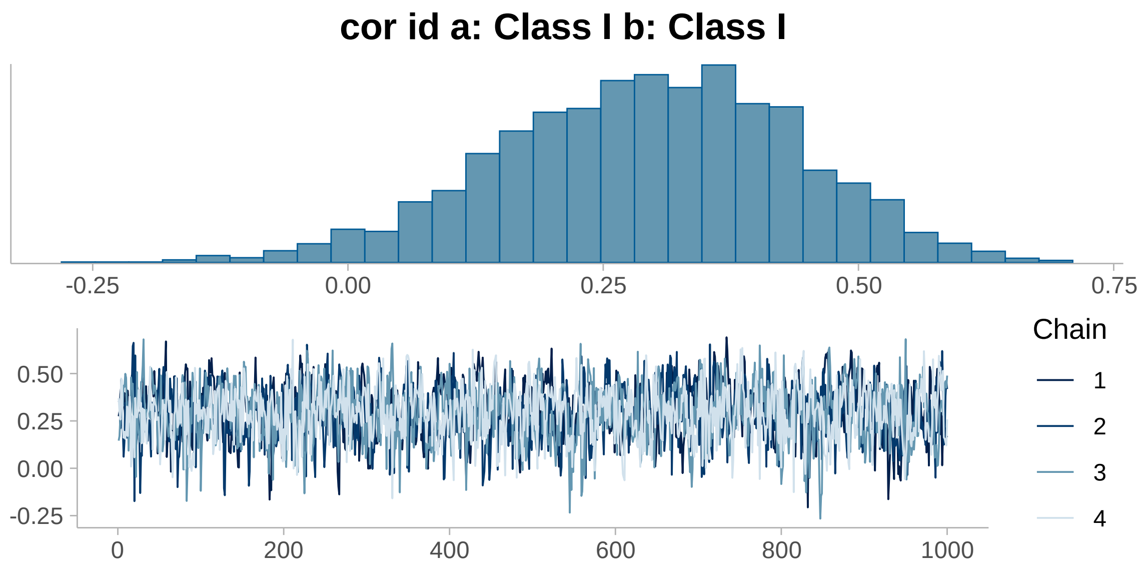
cor id a: Class I b: Class I
-0.25
0.00
0.25
0.50
0.75
Chain
1
2
3
4
0
200
400
600
800
1000

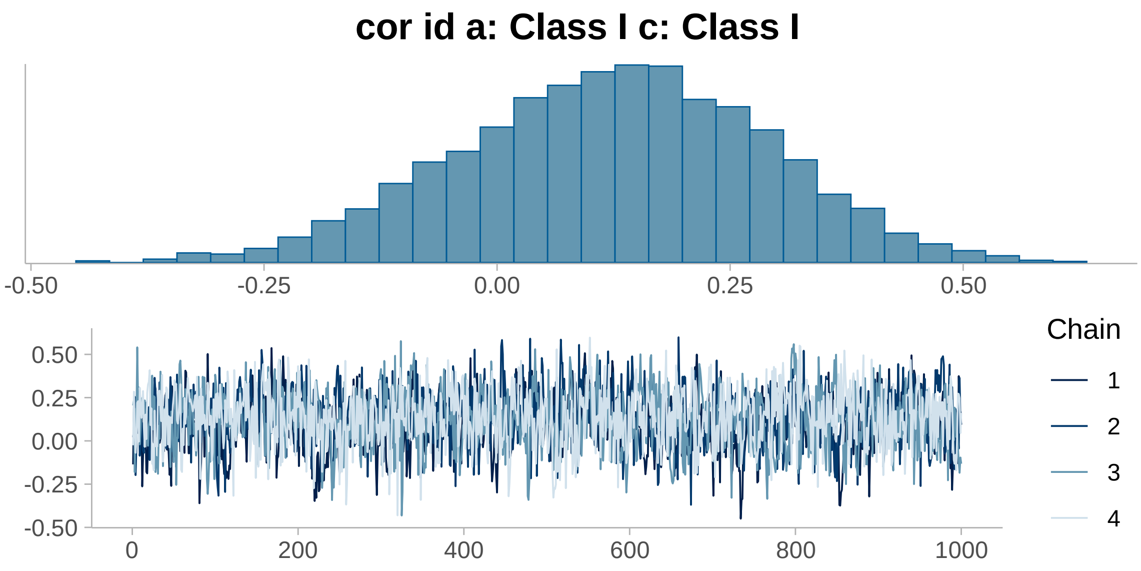

cor id a: Class I c: Class I
-0.50
-0.25
0.00
0.25
0.50
Chain
1
2
3
4
0.50
0.25
0.00
-0.25
-0.50
0
200
400
600
800
1000

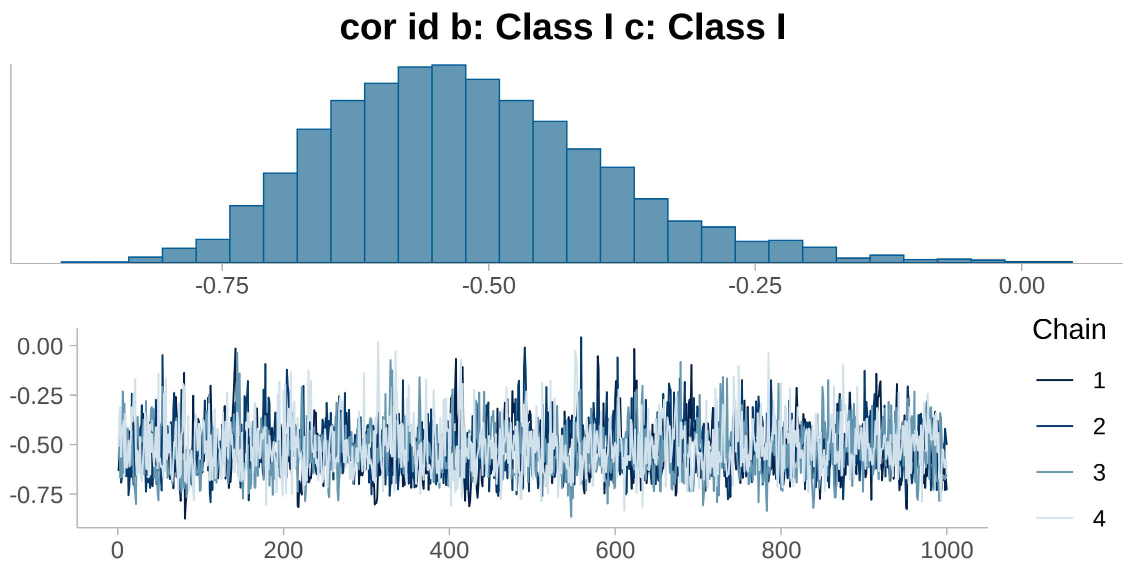
cor id b: Class I c: Class I
-0.75
-0.50
-0.25
0.00
Chain
1
2
3
4
0
200
400
600
800
1000

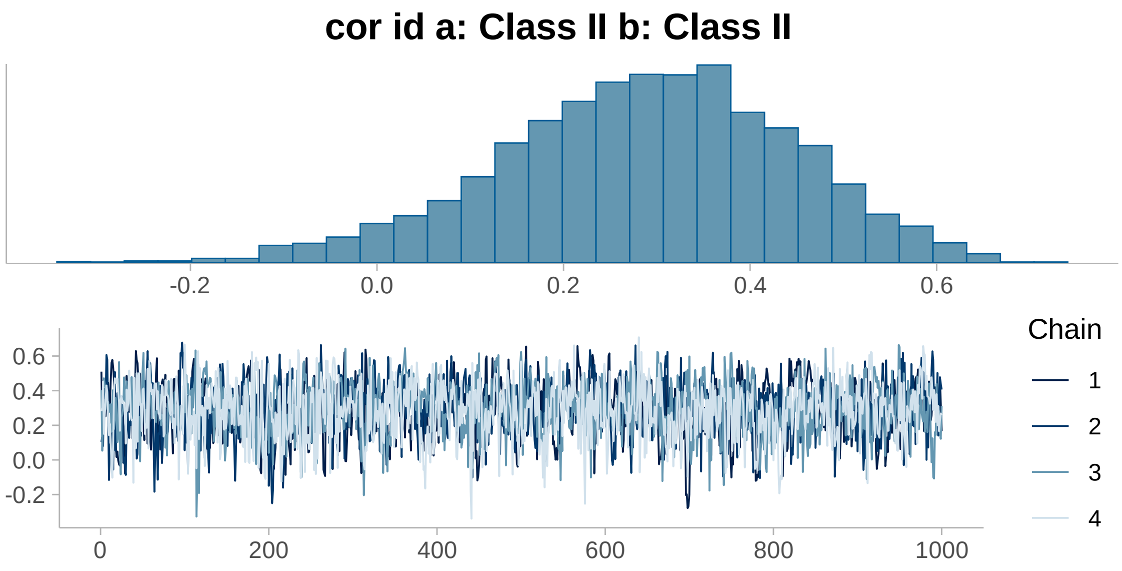
cor id a: Class II b: Class II
-0.2
0.0
0.2
0.4
0.6
Chain
1
2
3
4
0
200
400
600
800
1000

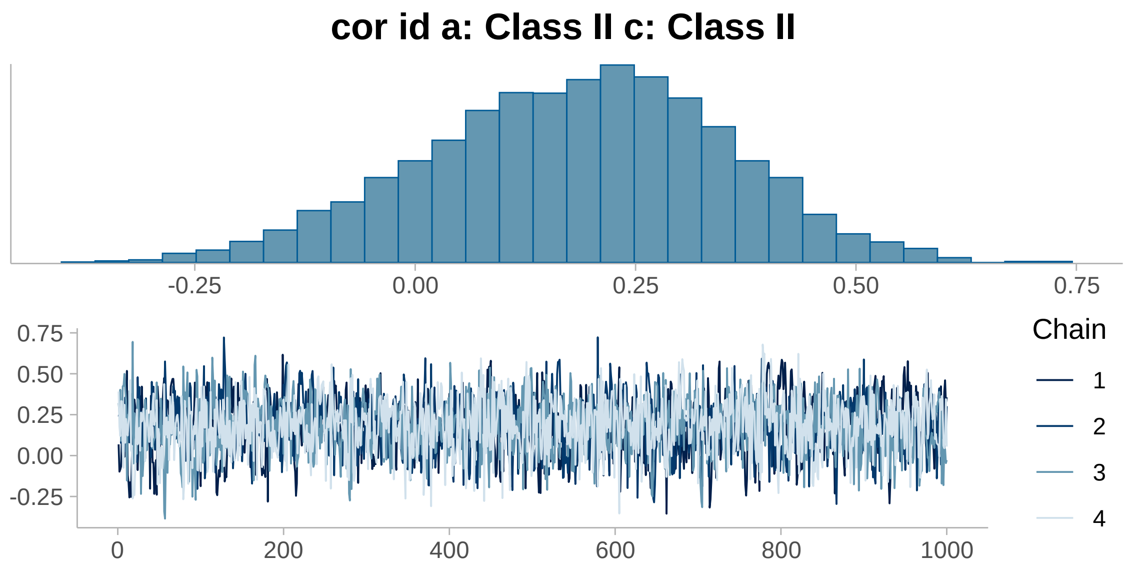
cor id a: Class II c: Class II
-0.25
0.00
0.25
0.50
0.75
0.75
0.50
0.25
0.00
-0.25
0
200
400
600
800
1000
Chain
1
2
3
4

cor id b: Class II c: Class II

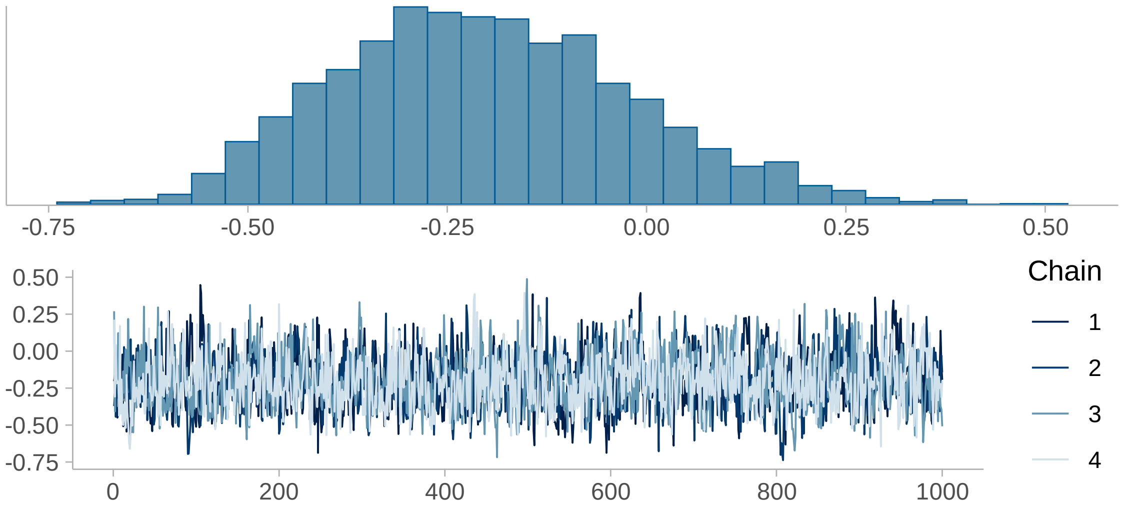

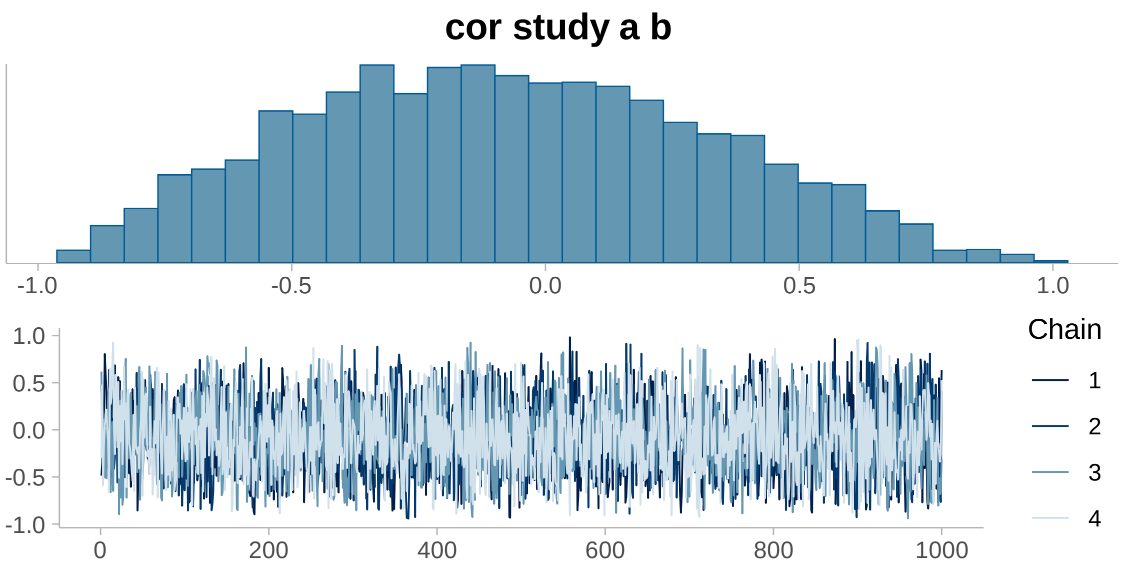
cor study a b
-1.0
-0.5
0.0
0.5
1.0
1.0
0.5
0.0
-0.5
-1.0
0
200
400
600
800
1000
Chain
1
2
3
4

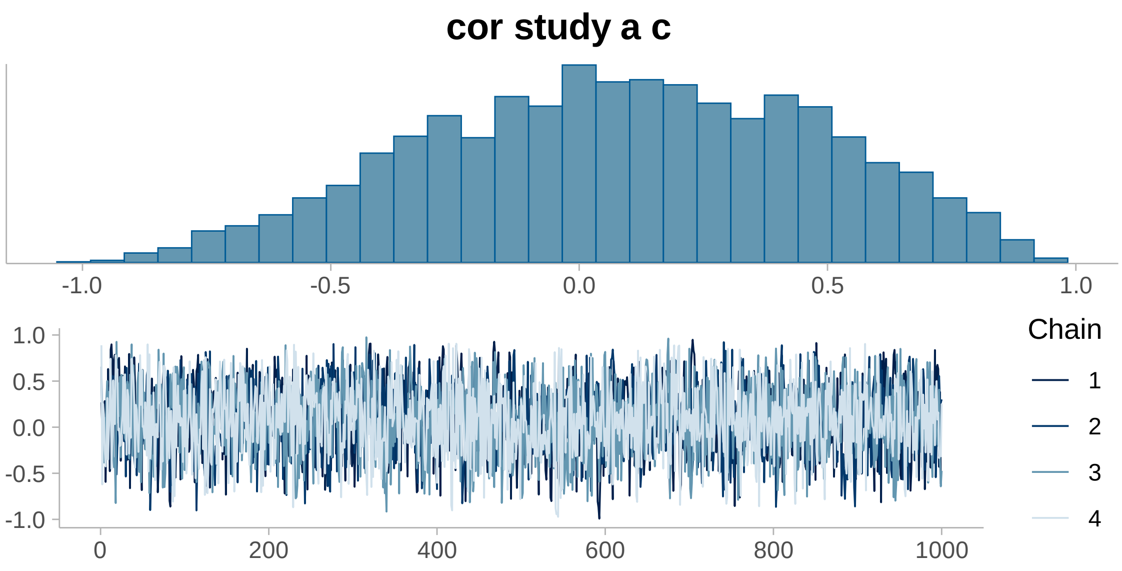
cor study a c
-1.0
-0.5
0.0
0.5
1.0
Chain
1
2
3
4
0
200
400
600
800
1000

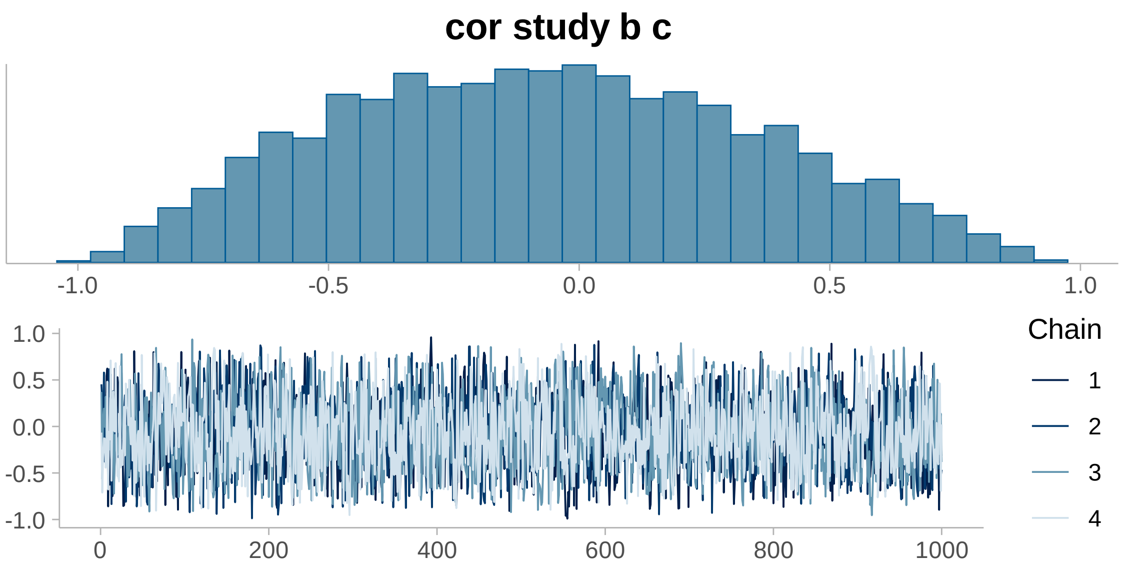

cor study b c
-1.0
-0.5
0.0
0.5
1.0
1.0
0.5
0.0
-0.5
-1.0
0
200
400
600
800
1000
Chain
1
2
3
4

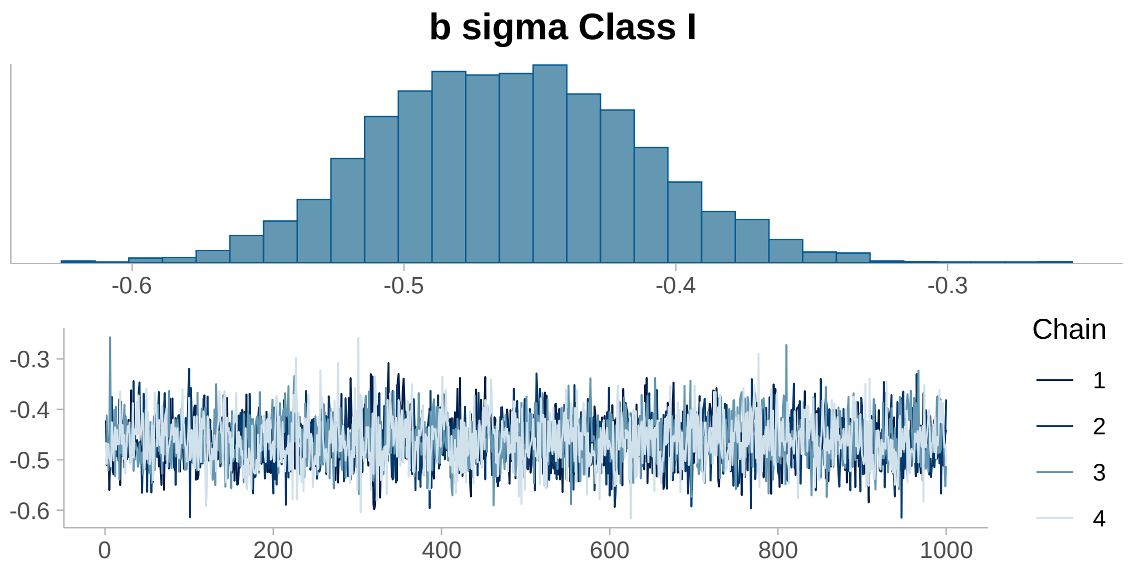
b sigma Class I
-0.6
-0.5
-0.4
-0.3
-0.3
-0.4
-0.5
-0.6
0
200
400
600
800
1000
Chain
1
2
3
4

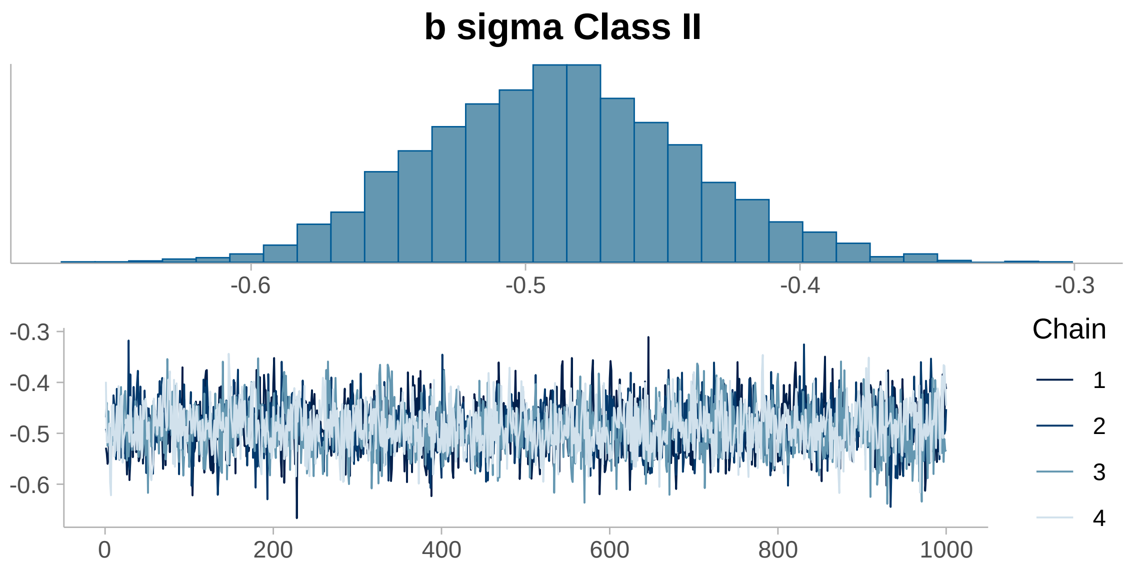
b sigma Class II
-0.6
-0.5
-0.4
-0.3
-0.3
-0.4
-0.5
-0.6
0
200
400
600
800
1000
Chain
1
2
3
4

References

1. Cole TJ, Donaldson MDC, Ben-Shlomo Y. SITAR—a useful instrument for growth curve analysis. Int J Epidemiol. 2010;39(6):1558-66.
2. Sandhu S. bsitar: Bayesian Super Imposition by Translation and Rotation Growth Curve Analysis. R package version 0.2.1.2024. Available from: https://CRAN.R-project.org/package=bsitar.
3. Gabry J, Goodrich B. rstanarm: Bayesian Applied Regression Modeling via Stan. R package version 232,1. 2024;2:0-3.
4. Buerkner PC. Advanced Bayesian Multilevel Modeling with the R

Package brms. The R Journal. 2018;10(1):395-411.
5. Stan Development Team. Stan Modeling Language Users Guide and Reference Manual, version 2.31. 2022.
